# An approach to analyse the specific impact of rapamycin on mRNA-ribosome association

**DOI:** 10.1186/1755-8794-1-33

**Published:** 2008-08-01

**Authors:** Raphael Genolet, Tanguy Araud, Laetitia Maillard, Pascale Jaquier-Gubler, Joseph Curran

**Affiliations:** 1 Department of Microbiology and Molecular Medicine, University of Geneva Medical School (CMU), 1 rue Michel Servet, CH-1211 Geneva, Switzerland

## Abstract

**Background:**

Recent work, using both cell culture model systems and tumour derived cell lines, suggests that the differential recruitment into polysomes of mRNA populations may be sufficient to initiate and maintain tumour formation. Consequently, a major effort is underway to use high density microarray profiles to establish molecular fingerprints for cells exposed to defined drug regimes. The aim of these pharmacogenomic approaches is to provide new information on how drugs can impact on the translational read-out within a defined cellular background.

**Methods:**

We describe an approach that permits the analysis of de-novo mRNA-ribosome association in-vivo during short drug exposures. It combines hypertonic shock, polysome fractionation and high-throughput analysis to provide a molecular phenotype of translationally responsive transcripts. Compared to previous translational profiling studies, the procedure offers increased specificity due to the elimination of the drugs secondary effects (e.g. on the transcriptional read-out). For this pilot "proof-of-principle" assay we selected the drug rapamycin because of its extensively studied impact on translation initiation.

**Results:**

High throughput analysis on both the light and heavy polysomal fractions has identified mRNAs whose re-recruitment onto free ribosomes responded to short exposure to the drug rapamycin. The results of the microarray have been confirmed using real-time RT-PCR. The selective down-regulation of TOP transcripts is also consistent with previous translational profiling studies using this drug.

**Conclusion:**

The technical advance outlined in this manuscript offers the possibility of new insights into mRNA features that impact on translation initiation and provides a molecular fingerprint for transcript-ribosome association in any cell type and in the presence of a range of drugs of interest. Such molecular phenotypes defined pre-clinically may ultimately impact on the evaluation of a particular drug in a living cell.

## Background

Dissecting the specific effect of a drug on a defined biological process is often complicated by the plethora of secondary effects that arise during extended exposure. This is highlighted by the anti-cancer drug rapamycin whose principle, although not exclusive, target is the rate limiting initiation step of protein translation [1]. Techniques designed to analyse the effect of the drug on mRNA-ribosome association are hampered by the long exposure times required to observe changes in the polysomal mRNA populations. Translation initiation is frequently regulated via the *e*ukaryotic *i*nitiation *f*actor 4E (eIF4E). It can be sequestered into an inactive complex by a family of 4E-binding proteins (4E-BP1/2/3). The affinity of these proteins for eIF4E is modulated by phosphorylation via the mTORC1 (mammalian target of rapamycin complex 1) kinase [2]. Rapamycin arrests many cells in G_1 _[3], and is a potent immunosuppressant [4]. It exerts its action by binding and inactivating the mTORC1 [5]. Previous studies have shown that extended exposure to rapamycin also alters transcription. Among the mRNAs regulated, a number impact directly on translation (e.g. eIF2α) [6]. These changes were minor after short drug exposure times (< 60 mins), but increased markedly after 2 hrs. Translational profiling studies (which examine the mRNAs associated with ribosomes) have also been reported [7-10]. One study, performed on the Jurkat T cell clone E6-1, revealed that after an extended exposure to rapamycin (minimum 4 hrs) almost all transcripts analysed were inhibited, and 136 of those (representing ~5% of the total) were strongly inhibited (at least 10 fold). This latter group included a number of mRNAs whose products impact directly on translation (e.g. eIF5A, eIF4A1, eEF1, eEFTu) [8]. This would predict that lengthy exposure to the drug will influence translation by modifying the levels of initiation and elongation factors. We therefore sought to develop a technique that would permit a specific analysis on how the drug alters transcript recruitment onto free ribosomes under conditions that eliminated the secondary effects associated with extended exposure. The approach has been coupled to a high-throughput microarray screen to examine how rapamycin exposure impacts on the re-seeding of the polysomal transcript populations. Results from the array have also been validated by quantitative RT-PCR.

## Methods

### Cell culture

MRC-5 cells (Coriell Cell Repository) were cultured in Minimal Essential Medium (Gibco) supplemented with 1 mM sodium pyruvate (Sigma), 0.1 mM non-essential amino acids, 10% foetal calf serum (Brunschwig), 1% penicillin/streptomycin, in a humidified atmosphere containing 5% CO_2_. For polysome analysis, cells in the growing phase (60% confluence) were hypertonically shocked by shifting to medium containing 300 mM NaCl for 50 min. They were then placed in normal isotonic medium for 30 min. When rapamycin was used, 100 nM rapamycin (LC laboratories) or 0.01% DMSO (the negative control) was added during the hypertonic shock, 20 min before the transfer back to isotonic conditions. Rapamycin and DMSO were kept on the cells throughout the 30 mins recovery period (total time of exposure to rapamycin was 50 mins). These conditions were based upon previously published work [11], although we have independently confirmed that they can be used on a range of cell lines including 293T [12], HeLa S3 and SK-NA5 (data not shown).

### Polysome gradient/RNA extraction

After treatment, cells were scraped into the culture medium and pelleted for 4 min at 100 g. The pellets, consisting of 5 × 10^6 ^cells, were lysed for 15 min on ice in 400 μL of 100 mM KCl, 50 mM Tris-Cl pH 7.4, 1.5 mM MgCl_2_, 1 mM DTT, 1 mg/mL heparin, 1.5 % NP40, 100 μM cycloheximide, 1% aprotinin, 1 mM AEBSF and 100 U/mL of RNasin. Nuclei were pelleted by centrifugation in a microfuge, 10 min at 12000 rpm. The supernatant was loaded onto a 20–60% sucrose gradient (in 100 mM KCL, 5 mM MgCl_2_, 20 mM HEPES pH 7.4 and 2 mM DTT). Extracts were fractionated for 3 h 30 min at 35,000 rpm at 4°C in a Beckman SW41 rotor, and the gradients were recovered in 3 fractions [monosome, light polysome (2 to 5 ribosomes) and heavy polysome (> 5 ribosomes)] using a Brandel gradient fractionator equipped with an ISCO UA-6 flow cell set to 254 nm. RNA was isolated from the light and heavy polysome fractions by adding an equal volume of TriZol (Invitrogen). Samples were mixed and incubate for 15 min. on ice, then 0.3 volumes of chloroform was added. After centrifugation, the upper phase was collected and the RNA precipitated with 0.7 volumes of isopropanol. The pellet of RNA was re-suspended in water. Prior to microarray analysis the pooled RNA fractions were further purified using the Qiagen RNeasy kit. The total yield of RNA in each pooled fraction was ~2 μg. RNA quality was checked on an Agilent 2100 bioanalyser.

### Microarray

Total RNA (100 ng from each fraction) was first amplified using the two step amplification protocol of Affymetrix. cRNA (17.5 μg) was then used to probe the Gene Chip U133 Plus 2.0 with 54,675 probe sets, covering more than 47,000 transcripts. Three biological replicates were hybridised for each condition (light and heavy polysomes +/- rapamycin). Data were analysed using the GCOS normalisation of Affymetrix. After normalisation, we filtered out probe sets that were assayed "absent" in all the 12 arrays. For this, the expression levels in each control experiment (DMSO) were arbitrarily fixed to one, and the fold change of the corresponding probe in the treated samples was normalised to this. The variation was then tested using the Mann and Witney U statistical test. Using this approach 24,105 of the probe sets (44%) were flagged as absent. The data was further analysed using two approaches. Firstly, probe set intensity values below 100 were removed prior to GCOS normalisation, and fold changes ≥ 1.5 relative to the DMSO control were scored (the smallest score in the three independent experiments had to show at least a 20% change relative to the DMSO control i.e. a 1.2 fold increase or a 0.8 fold decrease). In a second approach, commencing with the entire data set, points were only scored if the mean of the fold difference was ≥ 2.5 (once again the smallest score in the three independent experiments had to show at least a 20% change relative to the DMSO control). The data from both screens were plotted onto biological networks using the GO onthology (Affymetrix) and Ingenuity Pathway Analysis Software packages .

The microarray data are available at ArrayExpress (Accession N° E-TABM-205).

### Real-Time PCR

One μg of total RNA from a fourth independent experiment was reverse transcribed using random hexamers (Gibco). A 1/10 dilution of the cDNA was used to perform the PCR with the SYBR Green Reagent (Roche).

Primers used were:

**QKI **AGCATCACAGTCAGAGGTCAGC, GCAGTGGCATATTAAACCAAAGC;

**RBM7 **GTTGGAAATTCAAGCCCTACCT, AATCCTGATTGATCCAGAGGTG;

**ORMDL1 **GTCTGGCAGAAACAACGTCTC, CAATGTGGTTGCTGTTCTGG;

**FAS **GATGGCGAATGAGGTTCAG, CAATCCCATATCTCCCATTAAC;

**RBM17 **GTCATCTCCGGTGATCCTTAAA, CAACCAGAGAGGCACACAGAT;

**PAPPA **GCATCAGTTTCTCTAGCTGCAA, TATCAAACAAGCACTCCCTGTC;

**Actin **CTGACGGCCAGGTCATCACCATTG, GCCGGACTCGTCATACTCCTGCTTG;

**L27 **GTGACAGCTGCCATGGGCAAG, TCAAACTTGACCTTGGCCTCCCG,

**Cyclin D1 **AAGCAGGACTTTGAGGCA AG, CCTCTGAGGTCCCTACTTTCAA.

Primer sets were designed to amplify regions within the 3' UTR of each transcript since this generally corresponded to the site of the probe sets used on the Affymetrix chip. The specificity of each primer set was confirmed by standard RT-PCR on total cell RNA, followed by analysis of the DNA products by agarose gel electrophoresis (data not shown).

### Western blot analysis

Cells were lysed in CSH buffer (50 mM Tris-Cl pH 7.5, 250 mM NaCl, 1 mM EDTA, 0.1% Triton X-100) and the nuclei were pelleted by centrifugation at 12,000 rpm for 5 mins. Twenty μgs of protein was resolved on a 15% polyacrylamide-SDS gel and electrotransfered to a PVDF membrane. Antibodies used in this study were the anti-4EBP1 (Cell Signalling), the anti-phosho4EBPI (Thr37/46) (Cell Signalling), the anti-p70 S6 kinase (Cell Signalling), the anti-phospho-p70 S6 kinase (Thr389) (Cell Signalling) and mouse anti-actin (Chemicon). Blots were developed using the Super Signal Substrate (Thermo Scientific).

## Results

### Rapamycin delays mRNA recruitment onto polysomes

**Figure 1:**
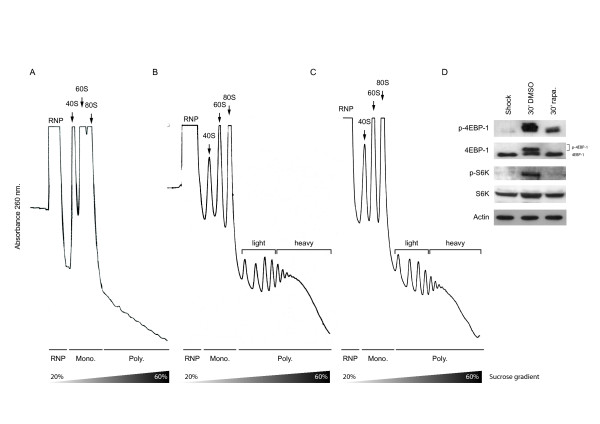
**Ribosomal re-recruitment in the presence of rapamycin**. (A). High salt provokes a rapid disaggregation of polysomes. (B). Upon restoration of isotonic conditions the polysomal fraction is reconstituted (C). Pre-treatment with rapamycin delays the re-recruitment of ribosomes. The position of the ribonucleoprotein (RNP), the monosomal (Mono) and polysomal (Poly) fractions are indicated. (D) Western blot analysis of phospho-4EBP-1, 4EBP-1, phospho-S6K, S6K, and actin was performed on extracts isolated under the different conditions depicted in panels (A); (B) and (C).

To directly examine the effect of rapamycin on mRNA recruitment we decided to exploit a novel approach, an approach that analyses the ability of cellular mRNAs to compete for free ribosomes in-vivo. Hypertonic shock provokes a rapid inhibition of protein synthesis, disaggregation of polysomes (Figure 1A), dephosphorylation of eIF4E, 4E-BP1, S6 and an increased association of eIF4E and 4E-BP1 [11,13]. Upon restoration of isotonic conditions the polysomal fraction is rapidly reconstituted (Figure 1B and [11]). Using this methodology it was possible to examine what effect rapamycin had on the recruitment of mRNA populations onto free ribosomes following very short drug exposure times. It was in substance an in-vivo competition assay performed under two defined physiological conditions. Drug treatment appeared to delay recruitment as evidenced by the reduction in the heavy polysome peak (≥ 6 ribosomes: compare Figure 1B and 1C), and this effect was correlated with a modification of the downstream signalling targets of mTOR, including 4E-BP1 and S6 kinase. (Figure 1D, compare the second and third lanes). This confirmed that despite the relatively short time of exposure to rapamycin, the recruitment assay monitored transcript:ribosome re-association under two conditions in which eIF4E availability was altered.

### A profiling screen identifies changes in the light and heavy polysomal mRNA populations

**Figure 2:**
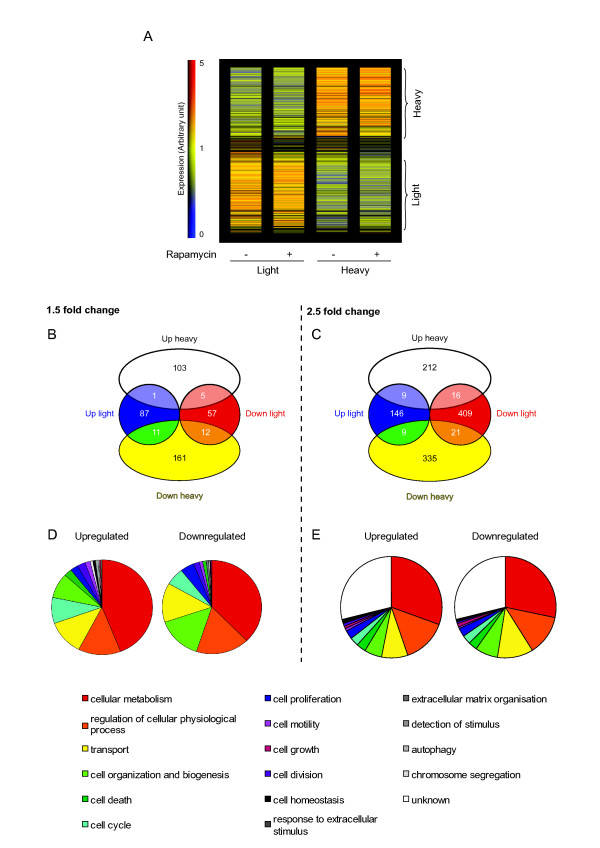
**Microarray analysis**. (A). Hierarchical clustering of relative expression. Each column represents the different conditions. On the right the vertical bar indicates probe set intensity values (indicated as Expression) in arbitrary units. The zero value indicates absence, with blue indicating a low level and red a high level of expression (the maximum value being fixed as 5). The vertical brackets on the right indicate that transcripts have been grouped into those over-represented in the light polysomes (lower) or in the heavy polysomes (upper). (B). After removal of probe set intensity values < 100, and the application of a ×1.5 fold cut-off, the regulated genes were classified into four groups. The values indicate the number of transcripts in each group. (C). In a second approach, a ×2.5 fold cut-off was applied. (D) and (E). Functional classification using Gene Ontology of the genes either up-regulated or down-regulated (as depicted in panel b and in Additional File 1, and panel c and in Additional File 2, respectively). The unknown fraction represents genes not annotated in the Gene Ontology database.

Equal amounts of RNA isolated from the light (2 to 5 ribosomes) and heavy (> 5 ribosomes) polysomal fractions were used to probe the Affymetrix Gene Chip U133 Plus 2. Triplicate independent gradients under each experimental condition were examined. After data analysis, two subpopulations of transcripts were clearly discriminated: those dominant in the light polysomal fraction and those dominant in the heavy polysomal fraction, an important criterion since it validated the initial experimental approach (Figure 2A). Despite the fact that the polysomal peaks were smaller in the presence of rapamycin, consistent with a global repression, the two sub-populations were essentially conserved (i.e. no major movement of mRNA populations between the two fractions as a consequence of the treatment was evident). To analyse the data we used two approaches. Firstly, we filtered out all transcripts giving low probe set intensity values on the chip using the default settings of the Agilent analysis software package (values < 100). We then scored for mRNAs whose polysomal occupancy was altered by greater than ×1.5 fold (listed in Additional File 1). This produced 437 transcripts within which was found the majority of the repressed TOP mRNAs (see below). Curiously, within this group of transcripts almost equal proportions were up and down-regulated (46% and 54%, respectively) (Figure 2B).

As a second approach to analyse the data we applied a ×2.5 fold change cut-off point to the entire data set. The rationale for this alternative analysis is based upon the fact that many of the genes that are regulated at the level of translation are frequently transcribed at low levels. This includes proto-oncogenes and other factors that regulate cell growth [14]. The majority of these transcripts are found within the lower intensity range and we therefore tested if meaningful information could be extracted from this region by applying a more stringent selection. We observed that 1160 mRNAs (3.8%) showed increased or decreased polysomal distribution in the presence of rapamycin, suggesting that in this small fraction of transcripts the affinity for the cap binding complex was changed (Figure 2C and Additional File 2). Over 2/3 of the mRNAs responding to the drug were down-regulated, whereas 1/3 showed increased polysomal occupancy relevant to the non-treated control. Only a few transcripts were regulated in both the heavy and the light polysomes (55 mRNAs) (Figure 2C). These results are not unlike those reported in a translational profiling study performed on the two tumoural cell lines, LAPC-4 (prostrate cancer) and U87 (glioblastoma). Applying the 2.5 fold cut-off point, ca. 6% of the 3,000 transcripts screened showed altered polysomal occupancy, and amongst these, 60% were down-regulated and 40% up-regulated [7].

Those mRNAs showing significant redistributions (both increased and decreased: as listed in Additional Files 1 and 2) were then plotted onto cellular networks using the GO onthology (Affymetrix) software package. Results revealed that transcripts up- or down-regulated affected more or less the same biological processes (Figure 2D and 2E). However, using the Ingenuity pathway analysis a number of features were immediately evident in the rapamycin treated cells: (a) A group of mRNAs involved in the inflammatory response were regulated, consistent with the immunosuppressive activity of rapamycin. Interestingly, we also observed down-regulation of several transcripts linked to phagocytosis. It has recently been reported that rapamycin down-regulates phagocytosis in a murine macrophage cell line (Table 1) [15]. (b) We observed a number of mRNAs involved in cell growth and proliferation. Among them, a number of anti-apoptotic mRNAs were up-regulated (e.g. relA and mdm2), whereas pro-apoptotic ones were down-regulated (e.g. fas, faslg and faf1) (Table 1). This is consistent with the observation that rapamycin did not induce apoptosis in MRC-5 cells even after extended exposure (unpublished observations), and corroborated recent studies showing the anti-apoptotic properties of rapamycin [16,17].

**Table 1:** List of transcripts regulated by rapamycin (Ingenuity classification)

**Inflammation**						
**Upregulated**		**Downregulated**				
	
apobec3f	lst1	adra1a	epha4	il1rn	ptpn22	tlr7
blnk	mdm2	adra2c	f9	irf2	ptprc	zeb1
cald1	oas1	alpp	fas	itgb3	ptprz1	
cmklr1	parvg	bcl11a	faslg	klf2	rbl2	
ctla4	pdgfc	bcl2l1	fcgr2a	lama3	rbm15	
cxcr4	rag2	cast	folr1	lilra2	rbpj	
cyp3a4	rela	cd28	fyb	mll	rel	
fyn	sat1	cd36	gal	mpo	satb1	
hla-g	tcf12	clec1b	gap43	pcgf2	siglec8	
ifih1	tfap4	cul4a	gnrh1	pla2g6	smpd1	
ifna2	thbs1	cxcl11	gnrhr	plcg1	spn	
il16	tlr7	cyp3a4	hal-dqb1	prdm16	syk	
il28b	unc119	ddl1	hck	pscdbp	thra	
itga4	vtcn1	dok2	ifne1	ptgs2	thrb	

**Cell growth and proliferation**						

**Upregulated**		**Downregulated**				
	
adam12	mdm2	adra1a	dok2	gnrh1	olig2	rffl
blnk	pappa	bcl2l1	f12	hck	p53aip1	sec14l2
cdc2l5	rag2	ccl27	fas	hmga2	pcgf2	syk
ctla4	rela	cd28	faslg	irf2	pdgfa	tfr2
cxcr4	s100b	cdca7	fbxo2	itgb3	piwil1	tgif1
erbb3	ss18	clca2	fcer2	klf2	pla2g6	thra
fyn	tcf12	cltc	fgf18	lzts1	ptgs2	thrb
hla-g	thbs1	csh2	folr1	mdm4	ptk2	tnfsf15
igf1r	unc119	cyp2c9	foxo1	mll	ptpn22	zfn10
il28b	vtcn1	dcc	gal	nab2	ptpra	
itga4		ddx17	gap43	nos1	ptprc	
lst1		dll1	glmn	nov	rbl2	

**Cell death**						

**Upregulated**		**Downregulated**				
	
blnk	itga2	abcd2	dll1	hmga2	p53aip1	rbm17
cdk6	mdm2	acvr1b	eraf	ifne1	pigt	rel
ctla4	rag2	adora2a	faf1	il1rn	piwil1	satb1
cul3	rela	atrx	fas	irf2	pla2g6	serpinb4
cxcr4	rnase1	atxn3	faslg	itgb3	pou4f1	siglec8
cyp2e1	sgpp1	bcl2l1	fcer2	klf2	ppp1r9b	smpd1
cyp3a4	tfap4	bircabp	foxo1	klra1	ppp2r1b	spn
erbb3	thbs1	cast	gal	lrp5	prdm2	stk4
erg	traf4	ccl27	gimap5	mdm4	ptgs2	syk
fyn	zmym2	cd28	gng2	mll	ptk2	thra
hla-g		cdk6	gria2	mpo	ptpn22	tnfsf15
ifih1		cyp2e1	grik2	nol3	ptprc	traf5
ifna2		cyp3a4	grm1	nos1	ptprz1	trps1
igf1r		dcc	hint1	nrtn	rbl2	znf10

**Phagocytosis**						

**Upregulated**		**Downregulated**				
		
		cd36	klf2			
		fas	mpo			
		fcgr2a	pla2g6			
		hck	syk			
		itgb3				

The 1160 mRNAs identified in the microarray screen (as listed in Additional File 2) were plotted onto biological networks using the Ingenuity Pathway Analysis Software package. Those whose protein products are involved in inflammation, phagocytosis, cell growth/proliferation and cell death are listed.

Rapamycin is known to have a marked effect on the expression of a subset of transcripts referred to as terminal oligopyrimidine (TOP) mRNAs [18]. This includes ribosomal protein mRNAs (estimated to be ~15% of the total cellular mRNA) and translation elongation factors. In previous translational profiling studies, a number of TOP mRNAs were clearly repressed. We also observed repression of ribosomal transcripts, although the effects were less extensive than in the earlier reports. This may reflect both the shorter drug exposure times and the experimental approach that was employed (i.e. mRNA re-recruitment onto free ribosomes). With the cut-off threshold at ×2.5 fold we observed down regulation of rpl14 and rpl21 in the light polysomes and rplp0 in the heavy polysomes (*r*ibosomal *p*rotein *l*arge). However, if the threshold was reduced to ×1.5 fold the number of hits significantly increased. Most significantly, ribosomal transcripts were only ever observed down-regulated (Table 2 and Additional File 1).

**Table 2:** List of TOP mRNAs detected in the array.

**LIGHT POLYSOME UP**	**LIGHT POLYSOME DOWN**	**HEAVY POLYSOME DOWN**
(NONE)	rpl5 (-1.5)	rplP0 (-2.7)
	rpl14 (-2.5)	rpl36 (-1.5)
	rpl21 (-5.1)	eef2 (-1.5)
	
**HEAVY POLYSOME UP**	rpl38 (-1.5)	(translation elongation factor)
	
(NONE)	rps11 (-2.1)	
	rps19 (-1.5)	
	rps21 (-2.1)	
	rps28 (-1.6)	
	eftuD1 (-5.5)	
	(translation elongation factor)	

### Independent confirmation of the array results by RT-PCR

**Figure 3:**
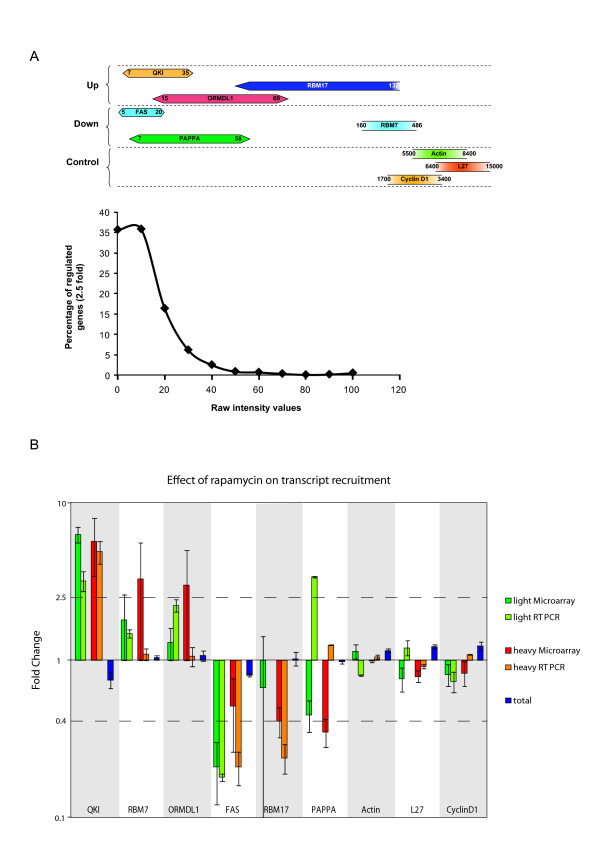
**Real-Time RT-PCR analysis**. (A). A schematic representation of the intensity values of transcripts selected for the RT-PCR validation. In the upper panel, the horizontal bars represented the value range for each mRNA on the array. The lower panel plots the distribution of regulated genes (×2.5 fold cut-off selection) relative to the probe set values. (B). RT-PCR values were normalised to those obtained from two housekeeping genes and the fold change indicates the difference in the DMSO and rapamycin values after normalisation. The DMSO value was arbitrarily set at 1. The results are compared with those obtained from the microarray. The variation in the total mRNA extracted is also represented. The 2.5 fold difference used for the screening is represented by the dotted lines (2.5 and 0.4). Each bar is representative of 2 independent RT-PCR assays performed in triplicate. Bars indicate the SEM.

With the aim of validating the microarray we performed real-time RT-PCR on selected transcripts across the spectrum of probe set intensities (Figure 3A). We were particularly interested in those transcripts that gave low probe set values. For the RT-PCR, RNA was extracted from a fourth independent experiment. Nine mRNAs were initially selected (Transcripts up-regulated = qki, rbm7, ormdl1. Transcripts down-regulated = fas, rbm17, pappa, Transcripts not regulated = β-actin, L27, cyclin D1: see Additional Files 1 and 2). Transcripts from the lower values of the data set (see Additional File 2 and Figure 3A) were selected because of the large fold difference between the rapamycin and DMSO control (> 2.5 fold). The results, with the exception of those obtained with the pappa transcript, largely confirmed the micro-array data (Figure 3B). This was particularly encouraging for transcripts such as those coding for QKI and FAS, indicating that application of the ×2.5 fold cut-off approach permitted the extraction of useful hits even in the lower end of the probe intensity set (Figure 3A). Note that the absence of a light RT-PCR value for RBM17 simply reflected its low levels in this fraction (Ct > 35 cycles). The microarray study also indicated that this transcript was regulated only within the heavy polysomal fraction, a result confirmed by the RT-PCR. In addition, these studies also demonstrated that no significant changes in total mRNA levels had occurred within the selected transcripts as a consequence of drug exposure (Figure 3B).

## Discussion

In this technical report, we have outlined a novel approach to translational profiling that follows the impact of a drug on the de-novo re-association of mRNAs and ribosomes in living cells in culture. An intracellular pool of free ribosomes was generated by a short hypertonic shock. Although this undoubtedly induced a stress response, previous work has demonstrated that the translation initiation machinery recovers very rapidly after the cells are transferred back to isotonic conditions as monitored by the reconstitution of the polysomal fraction [11,13]. The rapidity at which the polysomes are reformed has permitted us to examine the effect of a drug on this process using a short time window of exposure. This has the effect of limiting undesirable secondary effects that arise upon extended exposure, an effect highlighted by the drug selected for this study, namely rapamycin (see Introduction). The short exposure time warranted a drug concentration higher than that generally used in animal studies [19], however, some studies in cell culture systems have employed rapamycin concentrations as high as 15–20 μM before observing an impact on cell growth [20]. The selection was also dictated too by the fact that rapamycins' effect on translation initiation has been extensively studied. We have demonstrated that this approach can be coupled to a high-throughput analysis of the polysomal transcript populations.

Treatment with rapamycin limits the availability of eIF4E via its sequestration into an inactive complex with the hypophosphorylated 4E-BPs. Such a scenario represses global translation rates but with an effect more marked on those mRNAs containing structured 5' UTRs and those containing TOP elements. Indeed, when TOP containing transcripts were identified in our array they were always down-regulated (Table 2). Additionally, although the polysome profiles demonstrated an overall translational repression, the position of the vast majority of mRNAs on the gradient (i.e. the transcript populations in both the heavy and light polysomes) was largely unperturbed by the drug (i.e. there was little movement of transcripts from heavy to light polysomes, a somewhat unanticipated response), indicating that the affinity of re-recruitment of ribosomes onto these mRNAs (as reflected by the number of ribosomes per transcript) was largely unchanged.

A number of translational profiling studies examining the effect of rapamycin in mammalian cells have already been reported. In one of these, the effect of rapamycin on the polysomal distribution of mRNAs was demonstrated to be coupled to the activity of AKT [7], a result that demonstrates the extent to which a drugs effect can be modulated by the physiological status of the cell. This interpretation has become even more convoluted following the observation that prolonged rapamycin treatment may inhibit AKT signalling by interfering with the assembly of the second mTOR complex, mTORC2 [21]. However, with regards to the two transcripts characterised in this work as strongly up-regulated, namely cyclin D1 and c-myc, the former was down-regulated in our screen (×1.5 fold), and the latter gave values that were not considered statistically significant. Other studies have also failed to observe changes in the polysomal occupancy of the cyclin D1 mRNA in the presence of rapamycin [9,10,22]. These differences may reflect the cell lines and/or the experimental procedures employed. Both cyclin D1 and c-myc were proposed to carry IRESes within the 5' UTR, and IRES activity has been reported to show cell type specificity linked to the availability of ITAFs (*I*RES *T*rans-*A*cting *F*actors) [23]. Furthermore, the responsiveness of these IRESes to rapamycin was shown to be tightly coupled to the cellular activity of the AKT and RAF/MEK/ERK signalling cascades, features that may also show cell-type variation [24]. However, the slight reduction that we observed in the polysomal levels of the cyclin D1 mRNA would be consistent with other reports indicating that its expression was sensitive to the levels of eIF4E [25,26]. Finally, the earlier profiling study followed changes in the steady-state polysomal populations after extended exposure to the drug, whilst in the current work we have followed a competitive re-association. These processes may have altered initiation factor requirements, which could impact on the mRNA populations that respond. Indeed, it has been proposed that eIF4GII but not eIF4GI is required for re-initiation subsequent to a hypertonic shock [27]. Nonetheless, a listing of transcripts detected in both studies demonstrated that the majority behaved similarly (see Additional File 3).

## Conclusion

In summary, a major effort is underway to use high density microarray profiles to study how different drug regimes impact on the polysomal mRNA populations. These studies provide insights into how cellular gene expression is regulated at the level of translation initiation, the rate limiting step in protein expression. Changes in this read-out are a very rapid cellular response to physiological perturbations. The method that we have outlined permits a specific analysis of how a drug impacts on transcript-ribosome association. This early response almost certainly conditions subsequent cell behaviour during extended exposure. The choice of rapamycin for this "proof-of-principle" work was not arbitrary since the impact of this drug on translation initiation has been extensively studied. The technique offers the possibility of establishing molecular fingerprints for different tumour derived cell types and drug regimes [28]. In addition, it provides a very powerful technique to analyse the early events in translational control at the level of mRNA:ribosome association.

## Competing interests

The authors declare that they have no competing interests.

## Authors' contributions

RG and PJ-G prepared the polysomal RNA for the microarray analysis. RG and TA were involved in the analysis of the microarray data. RG and LM performed the RT-PCR control. JC prepared the manuscript. All authors have read and approved the manuscript.

## Pre-publication history

The pre-publication history for this paper can be accessed here:



## Supplementary Material

Additional File 1 List of transcripts from filtered data set. Transcripts with probe set values < 100 were removed from the data set. After GCOS normalisation a ×1.5 fold selection cut-off was applied to those that remained. Click here for file

Additional File 2 Complete list of polysomal transcripts regulated by rapamycin. After GCOS normalisation a ×2.5 fold selection cut-off was applied to all the regulated transcripts independent of probe set intensity values. Click here for file

Additional File 3 Comparison with the array of Gera and co-workers (7). Transcripts detected in both screens are listed with the fold change. Values are indicated in red when they differ between the two studies. Click here for file
