# Supplementary material for: An approach to analyse the specific impact of rapamycin on mRNA-ribosome association
Source: BMC Med Genomics. 2008 Aug 1;1:33. doi: 10.1186/1755-8794-1-33 (PMC2533349; doi:10.1186/1755-8794-1-33)
Supplement: Additional File 1 — List of transcripts from filtered data set. Transcripts with probe set values < 100 were removed from the data set. After GCOS normalisation a ×1.5 fold selection cut-off was applied to those that remained. [file 1755-8794-1-33-S1.doc]

| **Additional Table 1 : Polysomal transcripts regulated by rapamycin** | | | | | | |  |  |
| --- | --- | --- | --- | --- | --- | --- | --- | --- |
|  |  |  |  | **Fold change** | |  | | |
| **Affimetrix probe** | **Id** | **Gene symbol** | **Gene description** | **Light** | **Heavy** |  | | |
| 223182_s_at | None | *none* | 1-acylglycerol-3-phosphate O-acyltransferase 3 /// similar to 1-acylglycerol-3-phosphate O-acyltransferase 3 |  | -1.54 |  | | |
| 224812_at | NM_152740 | *hibadh* | 3-hydroxyisobutyrate dehydrogenase |  | 1.77 |  | | |
| 32029_at | NM_031268,NM_002613 | *pdpk1* | 3-phosphoinositide dependent protein kinase-1 |  | -1.50 |  | | |
| 218387_s_at | NM_012088 | *pgls* | 6-phosphogluconolactonase |  | -1.60 |  | | |
| 224742_at | None | *none* | abhydrolase domain containing 12 |  | -1.51 |  | | |
| 202003_s_at | NM_006111 | *acaa2* | acetyl-Coenzyme A acyltransferase 2 (mitochondrial 3-oxoacyl-Coenzyme A thiolase) | 1.63 |  |  | | |
| 201305_x_at | NM_006401 | *anp32b* | acidic (leucine-rich) nuclear phosphoprotein 32 family, member B | -1.56 |  |  | | |
| 200793_s_at | NM_001098 | *aco2* | aconitase 2, mitochondrial | 1.52 |  |  | | |
| 203952_at | NM_007348 | *atf6* | activating transcription factor 6 |  | 1.63 |  | | |
| 218405_at | NM_013375 | *abt1* | activator of basal transcription 1 | 1.54 |  |  | | |
| 235649_at | NM_007037 | *adamts8* | ADAM metallopeptidase with thrombospondin type 1 motif, 8 |  | -1.53 |  | | |
| 209635_at | NM_057089 | *ap1s1* | adaptor-related protein complex 1, sigma 1 subunit | -1.50 |  |  | | |
| 200615_s_at | NM_001282 | *ap2b1* | adaptor-related protein complex 2, beta 1 subunit | 1.55 |  |  | | |
| 208710_s_at | NM_003938 | *ap3d1* | adaptor-related protein complex 3, delta 1 subunit |  | -2.18 |  | | |
| 208967_s_at | NM_013411,NM_172199,NM_001625 | *ak2* | adenylate kinase 2 | 1.54 |  |  | | |
| 205609_at | NM_001146 | *angpt1* | angiopoietin 1 | -1.52 |  |  | | |
| 219437_s_at | NM_013275 | *ankrd11* | ankyrin repeat domain 11 |  | -2.03 |  | | |
| 223542_at | NM_032290 | *ankrd32* | ankyrin repeat domain 32 |  | 1.69 |  | | |
| 201715_s_at | NM_014977 | *acin1* | apoptotic chromatin condensation inducer 1 |  | 1.50 |  | | |
| 223223_at | NM_022786 | *arv1* | ARV1 homolog (S. cerevisiae) |  | 1.52 |  | | |
| 219918_s_at | NM_018136 | *aspm* | asp (abnormal spindle)-like, microcephaly associated (Drosophila) |  | 1.71 |  | | |
| 219374_s_at | NM_024740 | *alg9* | asparagine-linked glycosylation 9 homolog (S. cerevisiae, alpha- 1,2-mannosyltransferase) | 1.71 |  |  | | |
| 210896_s_at | NM_032466,NM_032468 | *asph* | aspartate beta-hydroxylase |  | 1.93 |  | | |
| 221492_s_at | NM_022488 | *apg3l* | ATG3 autophagy related 3 homolog (S. cerevisiae) |  | 1.58 |  | | |
| 218799_at | NM_018066 | *flj10349* | ATP binding domain 1 family, member B |  | 1.56 |  | | |
| 227237_x_at | NM_031921,NM_018188 | *atad3b|atad3a* | ATPase family, AAA domain containing 3B /// similar to AAA-ATPase TOB3 |  | -1.74 |  | | |
| 214594_x_at | None | *none* | ATPase, Class I, type 8B, member 1 |  | -1.80 |  | | |
| 202804_at | None | *none* | ATP-binding cassette, sub-family C (CFTR/MRP), member 1 | 1.63 |  |  | | |
| 222463_s_at | NM_138973,NM_138971,NM_138972,NM_012104 | *bace1* | beta-site APP-cleaving enzyme 1 |  | -1.78 |  | | |
| 212702_s_at | NM_015250,NM_001003800 | *bicd2* | bicaudal D homolog 2 (Drosophila) |  | -1.75 |  | | |
| 213905_x_at | NM_001711 | *bgn* | biglycan /// teashirt family zinc finger 1 |  | -1.69 |  | | |
| 212563_at | NM_015201 | *bop1* | block of proliferation 1 /// similar to block of proliferation 1 |  | -1.51 |  | | |
| 223134_at | NM_020235 | *bbx* | bobby sox homolog (Drosophila) | -1.76 | -1.89 |  | | |
| 204481_at | NM_004634,NM_001003694 | *brpf1* | bromodomain and PHD finger containing, 1 | -1.58 |  |  | | |
| 202102_s_at | NM_014299,NM_058243 | *brd4* | bromodomain containing 4 |  | -2.14 |  | | |
| 233041_x_at | None | *none* | BTB (POZ) domain containing 9 | -1.70 |  |  | | |
| 203538_at | NM_001745 | *camlg* | calcium modulating ligand | -1.59 | -1.52 |  | | |
| 214845_s_at | NM_001219 | *calu* | calumenin |  | 1.55 |  | | |
| 201949_x_at | NM_004930 | *capzb* | capping protein (actin filament) muscle Z-line, beta |  | -1.53 |  | | |
| 201941_at | NM_001304 | *cpd* | carboxypeptidase D | 1.60 |  |  | | |
| 209522_s_at | NM_144782,NM_004003,NM_000755 | *crat* | carnitine acetyltransferase |  | -1.69 |  | | |
| 1555889_a_at | NM_006371 | *crtap* | cartilage associated protein | -1.54 | -1.60 |  | | |
| 200766_at | NM_001909 | *ctsd* | cathepsin D (lysosomal aspartyl peptidase) |  | -1.64 |  | | |
| 224859_at | None | *none* | CD276 molecule |  | -1.52 |  | | |
| 214464_at | NM_014826,NM_003607 | *cdc42bpa* | CDC42 binding protein kinase alpha (DMPK-like) |  | -2.20 |  | | |
| 218157_x_at | NM_020239 | *cdc42se1* | CDC42 small effector 1 | -1.52 |  |  | | |
| 218740_s_at | NM_176096,NM_025197,NM_176095 | *cdk5rap3* | CDK5 regulatory subunit associated protein 3 | 1.68 |  |  | | |
| 203214_x_at | NM_033379,NM_001786 | *cdc2* | cell division cycle 2, G1 to S and G2 to M |  | 1.91 |  | | |
| 205046_at | NM_001813 | *cenpe* | centromere protein E, 312kDa |  | 1.73 |  | | |
| 239413_at | None | *none* | centrosomal protein 152kDa |  | 1.62 |  | | |
| 205250_s_at | NM_025114 | *cep290* | centrosomal protein 290kDa |  | 1.56 |  | | |
| 203491_s_at | NM_014679 | *pig8* | centrosomal protein 57kDa |  | -2.37 |  | | |
| 235117_at | NM_001008708 | *loc494143* | ChaC, cation transport regulator-like 2 (E. coli) |  | 1.75 |  | | |
| 200877_at | NM_006430 | *cct4* | chaperonin containing TCP1, subunit 4 (delta) | 1.53 |  |  | | |
| 208696_at | NM_012073 | *cct5* | chaperonin containing TCP1, subunit 5 (epsilon) | 1.51 |  |  | | |
| 38069_at | NM_001287 | *clcn7* | chloride channel 7 |  | -1.56 |  | | |
| 1555543_a_at | NM_015127 | *mclc* | chloride channel CLIC-like 1 |  | 1.74 |  | | |
| 222235_s_at | NM_018590,NM_001007535 | *galnact-2|dj341d10.1* | chondroitin sulfate GalNAcT-2 /// similar to chondroitin beta1,4 N-acetylgalactosaminyltransferase 2 |  | 1.66 |  | | |
| 209715_at | NM_012117 | *cbx5* | chromobox homolog 5 (HP1 alpha homolog, Drosophila) | -1.52 |  |  | | |
| 208835_s_at | NM_006107,NM_016424 | *crop* | cisplatin resistance-associated overexpressed protein |  | -1.76 |  | | |
| 225893_at | None | *none* | Clone TESTIS-724 mRNA sequence |  | 1.58 |  | | |
| 1556346_at | None | *none* | Coactosin-like 1 (Dictyostelium) | -1.64 |  |  | | |
| 208684_at | NM_004371 | *copa* | coatomer protein complex, subunit alpha | 1.56 |  |  | | |
| 228693_at | None | *none* | coiled-coil domain containing 50 | -1.60 |  |  | | |
| 221791_s_at | NM_015933 | *hspc016* | coiled-coil domain containing 72 | -1.61 |  |  | | |
| 225241_at | NM_199512,NM_199511 | *urb* | coiled-coil domain containing 80 |  | -2.38 |  | | |
| 211966_at | NM_001846 | *col4a2* | collagen, type IV, alpha 2 | 1.89 |  |  | | |
| 52255_s_at | NM_015719 | *col5a3* | collagen, type V, alpha 3 |  | -1.71 |  | | |
| 204320_at | NM_080630,NM_001854,NM_080629 | *col11a1* | collagen, type XI, alpha 1 |  | -1.63 |  | | |
| 218351_at | NM_017845 | *commd8* | COMM domain containing 8 |  | 1.59 |  | | |
| 64486_at | NM_020441 | *coro1b* | coronin, actin binding protein, 1B |  | -1.57 |  | | |
| 200884_at | NM_001823 | *ckb* | creatine kinase, brain |  | -1.65 |  | | |
| 217844_at | NM_021198 | *ctdsp1* | CTD (carboxy-terminal domain, RNA polymerase II, polypeptide A) small phosphatase 1 |  | -1.63 |  | | |
| 208711_s_at | NM_053056 | *ccnd1* | cyclin D1 |  | -1.75 |  | | |
| 224847_at | NM_001259 | *cdk6* | cyclin-dependent kinase 6 |  | 1.52 |  | | |
| 1555278_a_at | NM_014756,NM_001008938 | *ch-tog* | cytoskeleton associated protein 5 |  | 1.72 |  | | |
| 222101_s_at | NM_003737 | *dchs1* | dachsous 1 (Drosophila) |  | -1.67 |  | | |
| 204244_s_at | NM_006716 | *ask* | DBF4 homolog (S. cerevisiae) |  | 1.63 |  | | |
| 204355_at | NM_138615,NM_014966 | *dhx30* | DEAH (Asp-Glu-Ala-His) box polypeptide 30 | 1.53 |  |  | | |
| 1553768_a_at | NM_173674 | *dcbld1* | discoidin, CUB and LCCL domain containing 1 |  | -3.07 |  | | |
| 202843_at | NM_012328 | *dnajb9* | DnaJ (Hsp40) homolog, subfamily B, member 9 |  | 1.54 |  | | |
| 216835_s_at | NM_001381 | *dok1* | docking protein 1, 62kDa (downstream of tyrosine kinase 1) |  | -1.53 |  | | |
| 1554966_a_at | NM_014890,NM_182909 | *doc1* | downregulated in ovarian cancer 1 |  | 1.55 |  | | |
| 225633_at | None | *none* | dpy-19-like 3 (C. elegans) | 1.54 |  |  | | |
| 213391_at | NM_181787 | *loc286148* | dpy-19-like 4 (C. elegans) |  | 1.56 |  | | |
| 224129_s_at | NM_032574 | *loc84661* | dpy-30-like protein | 1.50 |  |  | | |
| 208892_s_at | NM_022652,NM_001946 | *dusp6* | dual specificity phosphatase 6 | -1.50 | -1.76 |  | | |
| 229115_at | NM_001376 | *dnch1* | dynein, cytoplasmic 1, heavy chain 1 |  | -1.61 |  | | |
| 216212_s_at | NM_001363 | *dkc1* | dyskeratosis congenita 1, dyskerin |  | -1.94 |  | | |
| 230629_s_at | NM_015409 | *ep400* | E1A binding protein p400 |  | -2.40 |  | | |
| 203692_s_at | NM_001949 | *e2f3* | E2F transcription factor 3 | -1.65 |  |  | | |
| 213895_at | None | *none* | epithelial membrane protein 1 |  | 1.54 |  | | |
| 223243_s_at | NM_025191 | *c1orf22* | ER degradation enhancer, mannosidase alpha-like 3 /// similar to chromosome 1 open reading frame 22 |  | 1.73 |  | | |
| 228162_at | None | *none* | Esterase D/formylglutathione hydrolase | -1.54 |  |  | | |
| 200094_s_at | NM_001961 | *eef2* | eukaryotic translation elongation factor 2 /// eukaryotic translation elongation factor 2 |  | -1.50 |  | | |
| 200005_at | NM_003753 | *eif3s7* | eukaryotic translation initiation factor 3, subunit 7 zeta, 66/67kDa /// eukaryotic translation initiation factor 3, subunit 7 zeta, 66/67kDa | 1.73 |  |  | | |
| 215230_x_at | NM_003752 | *eif3s8* | eukaryotic translation initiation factor 3, subunit 8, 110kDa /// similar to eukaryotic translation initiation factor 3, subunit 8 | 1.83 |  |  | | |
| 201435_s_at | NM_001968 | *eif4e* | eukaryotic translation initiation factor 4E |  | 1.58 |  | | |
| 213757_at | None | *none* | Eukaryotic translation initiation factor 5A | -1.72 |  |  | | |
| 201574_at | NM_004730 | *etf1* | eukaryotic translation termination factor 1 |  | 1.52 |  | | |
| 212630_at | NM_007277 | *sec6l1* | exocyst complex component 3 |  | -1.70 |  | | |
| 210933_s_at | NM_003088 | *fscn1* | fascin homolog 1, actin-bundling protein (Strongylocentrotus purpuratus) | -1.81 |  |  | | |
| 208963_x_at | NM_013402 | *fads1* | fatty acid desaturase 1 |  | -1.68 |  | | |
| 209455_at | NM_033645,NM_033644,NM_012300 | *fbxw11* | F-box and WD-40 domain protein 11 | 1.61 |  |  | | |
| 215600_x_at | NM_003677 | *denr* | F-box and WD-40 domain protein 12 |  | -1.60 |  | | |
| 209630_s_at | None | *none* | F-box and WD-40 domain protein 2 | 1.53 |  |  | | |
| 218432_at | NM_012175 | *fbxo3* | F-box protein 3 |  | 1.71 |  | | |
| 203184_at | NM_001999 | *fbn2* | fibrillin 2 (congenital contractural arachnodactyly) | 1.54 |  |  | | |
| 212464_s_at | NM_212474,NM_212476,NM_212478,NM_002026 | *fn1* | fibronectin 1 | 1.56 |  |  | | |
| 202304_at | NM_014923 | *fndc3a* | fibronectin type III domain containing 3A |  | 1.54 |  | | |
| 203886_s_at | NM_001998,NM_001004019 | *fbln2* | fibulin 2 |  | -1.50 |  | | |
| 203088_at | NM_006329 | *fbln5* | fibulin 5 | 1.54 |  |  | | |
| 213746_s_at | NM_001456 | *flna* | filamin A, alpha (actin binding protein 280) | 1.55 |  |  | | |
| 207876_s_at | NM_001458 | *flnc* | filamin C, gamma (actin binding protein 280) |  | -1.57 |  | | |
| 1553613_s_at | NM_001453 | *foxc1* | forkhead box C1 |  | -1.52 |  | | |
| 213940_s_at | NM_015033 | *fnbp1* | formin binding protein 1 |  | -1.81 |  | | |
| 218818_at | NM_004468 | *fhl3* | four and a half LIM domains 3 | -1.52 |  |  | | |
| 203987_at | NM_003506 | *fzd6* | frizzled homolog 6 (Drosophila) |  | 1.55 |  | | |
| 227405_s_at | NM_031866 | *fzd8* | frizzled homolog 8 (Drosophila) |  | -1.77 |  | | |
| 213979_s_at | None | *none* | Full-length cDNA clone CS0DF027YP13 of Fetal brain of Homo sapiens (human) | -1.67 |  |  | | |
| 218151_x_at | NM_024531 | *gpr172a* | G protein-coupled receptor 172A |  | -1.52 |  | | |
| 220642_x_at | NM_016334 | *gpr89* | G protein-coupled receptor 89A /// similar to G protein-coupled receptor 89 | 1.61 |  |  | | |
| 211040_x_at | NM_016426 | *gtse1* | G-2 and S-phase expressed 1 /// G-2 and S-phase expressed 1 |  | -1.52 |  | | |
| 202356_s_at | NM_002096 | *gtf2f1* | general transcription factor IIF, polypeptide 1, 74kDa |  | -1.66 |  | | |
| 207525_s_at | NM_202494,NM_202469,NM_202467,NM_202470 | *rgs19ip1* | GIPC PDZ domain containing family, member 1 | -1.66 |  |  | | |
| 204221_x_at | None | *none* | GLI pathogenesis-related 1 (glioma) |  | 1.56 |  | | |
| 227022_at | NM_138335 | *gnpda2* | glucosamine-6-phosphate deaminase 2 |  | 1.52 |  | | |
| 213552_at | NM_015554 | *glce* | glucuronyl C5-epimerase |  | 1.51 |  | | |
| 200842_s_at | NM_004446 | *eprs* | glutamyl-prolyl-tRNA synthetase |  | -2.23 |  | | |
| 206662_at | NM_002064 | *glrx* | glutaredoxin (thioltransferase) |  | 1.53 |  | | |
| 200723_s_at | NM_005898 | *m11s1* | GPI-anchored membrane protein 1 | 1.57 |  |  | | |
| 204472_at | NM_181702,NM_005261 | *gem* | GTP binding protein overexpressed in skeletal muscle |  | 1.51 |  | | |
| 222834_s_at | NM_018841 | *gng12* | guanine nucleotide binding protein (G protein), gamma 12 |  | -2.22 |  | | |
| 202615_at | None | *none* | Guanine nucleotide binding protein (G protein), q polypeptide |  | -1.60 |  | | |
| 214501_s_at | NM_004893,NM_138609,NM_138610 | *h2afy* | H2A histone family, member Y |  | -1.82 |  | | |
| 224602_at | NM_001001701 | *loc401152* | HCV F-transactivated protein 1 | 1.61 |  |  | | |
| 201841_s_at | NM_001540 | *hspb1* | heat shock 27kDa protein 1 |  | 1.55 |  | | |
| 208814_at | None | *none* | Heat shock 70kDa protein 4 |  | -1.63 |  | | |
| 210338_s_at | NM_153201,NM_006597 | *hspa8* | heat shock 70kDa protein 8 | 1.57 |  |  | | |
| 200598_s_at | NM_003299 | *tra1* | heat shock protein 90kDa beta (Grp94), member 1 |  | -1.64 |  | | |
| 218632_at | NM_024602 | *flj21156* | HECT domain containing 3 |  | -1.59 |  | | |
| 212822_at | None | *none* | HEG homolog 1 (zebrafish) |  | 1.70 |  | | |
| 210428_s_at | NM_004712 | *hgs* | hepatocyte growth factor-regulated tyrosine kinase substrate |  | -1.51 |  | | |
| 200896_x_at | NM_004494,NM_138574 | *hdgf|pwwp1* | hepatoma-derived growth factor (high-mobility group protein 1-like) |  | -1.55 |  | | |
| 1561633_at | None | *none* | high mobility group AT-hook 2 | -2.00 | -1.51 |  | | |
| 204504_s_at | NM_003609 | *hirip3* | HIRA interacting protein 3 |  | 1.64 |  | | |
| 203253_s_at | NM_015216 | *kiaa0433* | Histidine acid phosphatase domain containing 1 |  | 1.63 |  | | |
| 205967_at | NM_003542 | *hist1h4c* | histone 1, H4c | -1.54 | -3.05 |  | | |
| 211528_x_at | NM_002127,XR_000297,NM_005516,NR_001434 | *hla-g|hla-e|hla-a|hla-f* | HLA-G histocompatibility antigen, class I, G | 1.60 | 2.08 |  | | |
| 225115_at | None | *none* | Homeodomain interacting protein kinase 2 |  | -2.06 |  | | |
| 204818_at | NM_002153 | *hsd17b2* | hydroxysteroid (17-beta) dehydrogenase 2 | 1.59 |  |  | | |
| 225381_at | None | *none* | hypothetical gene supported by BX647608 |  | 1.63 |  | | |
| 226559_at | NM_203434 | *ier5l* | immediate early response 5-like |  | -1.57 |  | | |
| 221688_s_at | NM_018285 | *c15orf12* | IMP3, U3 small nucleolar ribonucleoprotein, homolog (yeast) | 1.69 |  |  | | |
| 210970_s_at | NM_015525 | *ibtk* | inhibitor of Bruton agammaglobulinemia tyrosine kinase |  | 1.56 |  | | |
| 217496_s_at | NM_004969 | *ide* | insulin-degrading enzyme |  | 1.57 |  | | |
| 218847_at | NM_001007225,NM_006548 | *imp-2* | insulin-like growth factor 2 mRNA binding protein 2 |  | -1.50 |  | | |
| 203819_s_at | NM_006547 | *imp-3* | insulin-like growth factor 2 mRNA binding protein 3 |  | -1.65 |  | | |
| 203424_s_at | NM_000599 | *igfbp5* | insulin-like growth factor binding protein 5 |  | -1.84 |  | | |
| 210213_s_at | NM_181467,NM_181466,NM_181469,NM_002212 | *itgb4bp* | integrin beta 4 binding protein | 1.56 |  |  | | |
| 205032_at | NM_002203 | *itga2* | integrin, alpha 2 (CD49B, alpha 2 subunit of VLA-2 receptor) |  | 1.71 |  | | |
| 1553678_a_at | NM_002211 | *itgb1* | integrin, beta 1 (fibronectin receptor, beta polypeptide, antigen CD29 includes MDF2, MSK12) |  | 1.70 |  | | |
| 202147_s_at | NM_001550,NM_001007245 | *ifrd1* | interferon-related developmental regulator 1 | 1.50 |  |  | | |
| 209297_at | NM_001001132 | *itsn1* | intersectin 1 (SH3 domain protein) | 1.96 |  |  | | |
| 210418_s_at | NM_174855,NM_006899,NM_174856 | *idh3b* | isocitrate dehydrogenase 3 (NAD+) beta | 1.92 |  |  | | |
| 222728_s_at | NM_024116 | *mgc5306* | Josephin domain containing 3 |  | -1.54 |  | | |
| 221763_at | NM_004241 | *jmjd1c* | jumonji domain containing 1C |  | 1.50 |  | | |
| 211762_s_at | NM_002266 | *kpna2* | karyopherin alpha 2 (RAG cohort 1, importin alpha 1) /// karyopherin alpha 2 (RAG cohort 1, importin alpha 1) /// similar to Importin alpha-2 subunit (Karyopherin alpha-2 subunit) (SRP1-alpha) (RAG cohort protein 1) /// similar to Importin alpha-2 subunit (Karyopherin alpha-2 subunit) (SRP1-alpha) (RAG cohort protein 1) | 1.52 |  |  | | |
| 206551_x_at | NM_017644 | *dre1* | kelch-like 24 (Drosophila) | -1.70 |  |  | | |
| 204372_s_at | NM_003685 | *khsrp* | KH-type splicing regulatory protein (FUSE binding protein 2) |  | -1.63 |  | | |
| 200914_x_at | NM_182926 | *ktn1* | kinectin 1 (kinesin receptor) | -1.52 | -1.51 |  | | |
| 201030_x_at | NM_002300 | *ldhb* | lactate dehydrogenase B | 1.50 |  |  | | |
| 216264_s_at | NM_002292 | *lamb2* | laminin, beta 2 (laminin S) | 1.59 |  |  | | |
| 223200_s_at | NM_018385 | *flj11301* | large subunit GTPase 1 homolog (S. cerevisiae) |  | -1.67 |  | | |
| 202728_s_at | NM_000627,NM_206943 | *ltbp1* | latent transforming growth factor beta binding protein 1 |  | -1.91 |  | | |
| 208949_s_at | NM_194327,NM_002306 | *lgals3* | lectin, galactoside-binding, soluble, 3 (galectin 3) /// galectin-3 internal gene |  | -1.59 |  | | |
| 217933_s_at | NM_015907 | *lap3* | leucine aminopeptidase 3 | 1.64 |  |  | | |
| 220750_s_at | NM_022356 | *lepre1* | leucine proline-enriched proteoglycan (leprecan) 1 | 1.58 |  |  | | |
| 1558173_a_at | NM_033631 | *luzp1* | leucine zipper protein 1 |  | -2.02 |  | | |
| 203412_at | NM_006767 | *lztr1* | leucine-zipper-like transcription regulator 1 |  | -1.50 |  | | |
| 228454_at | NM_032440 | *mlr2* | ligand-dependent corepressor |  | 1.73 |  | | |
| 222457_s_at | NM_016357 | *eplin* | LIM domain and actin binding 1 |  | -1.84 |  | | |
| 209204_at | NM_006769 | *lmo4* | LIM domain only 4 | -1.72 | -1.53 |  | | |
| 200785_s_at | NM_002332 | *lrp1* | low density lipoprotein-related protein 1 (alpha-2-macroglobulin receptor) |  | -1.61 |  | | |
| 220253_s_at | NM_013437 | *lrp12* | low density lipoprotein-related protein 12 |  | -1.69 |  | | |
| 224480_s_at | NM_032717 | *mgc11324* | lysophosphatidic acid acyltransferase theta /// lysophosphatidic acid acyltransferase theta |  | -1.62 |  | | |
| 200079_s_at | NM_005548 | *kars* | lysyl-tRNA synthetase /// lysyl-tRNA synthetase | 1.58 |  |  | | |
| 217456_x_at | NM_005516 | *hla-e* | major histocompatibility complex, class I, E |  | -1.50 |  | | |
| 202180_s_at | NM_005115,NM_017458 | *mvp* | major vault protein | 1.52 |  |  | | |
| 209166_s_at | NM_000528 | *man2b1* | mannosidase, alpha, class 2B, member 1 |  | -1.54 |  | | |
| 203778_at | NM_005908 | *manba* | mannosidase, beta A, lysosomal |  | 1.53 |  | | |
| 222805_at | NM_024641 | *manea* | mannosidase, endo-alpha | 1.57 |  |  | | |
| 213422_s_at | NM_032348 | *mgc3047* | matrix-remodelling associated 8 |  | -1.50 |  | | |
| 223570_at | NM_018518,NM_182751 | *mcm10* | MCM10 minichromosome maintenance deficient 10 (S. cerevisiae) | -1.50 | 1.54 |  | | |
| 210983_s_at | NM_005916,NM_182776 | *mcm7* | MCM7 minichromosome maintenance deficient 7 (S. cerevisiae) | 1.59 |  |  | | |
| 226958_s_at | NM_001001683 | *mgc88387* | mediator of RNA polymerase II transcription, subunit 11 homolog (S. cerevisiae) | 1.51 |  |  | | |
| 212305_s_at | None | *none* | melanoma inhibitory activity family, member 3 |  | 1.55 |  | | |
| 201736_s_at | NM_005885 | *march-vi* | membrane-associated ring finger (C3HC4) 6 | 1.64 |  |  | | |
| 226528_at | NM_001010891 | *mtx3* | metaxin 3 |  | 1.76 |  | | |
| 219451_at | NM_012228 | *msrb2* | methionine sulfoxide reductase B2 |  | -1.62 |  | | |
| 1554127_s_at | NM_198080 | *msrb3* | methionine sulfoxide reductase B3 |  | -1.56 |  | | |
| 209580_s_at | NM_003925 | *mbd4* | methyl-CpG binding domain protein 4 |  | 1.50 |  | | |
| 1565162_s_at | NM_145792,NM_145764,NM_020300,NM_145791 | *mgst1* | microsomal glutathione S-transferase 1 |  | 1.77 |  | | |
| 221559_s_at | NM_024039 | *mis12* | MIS12 homolog (yeast) |  | 1.51 |  | | |
| 214246_x_at | NM_015716,NM_170663,NM_153827 | *mink1* | misshapen-like kinase 1 (zebrafish) |  | -1.66 |  | | |
| 222555_s_at | NM_022915 | *mrpl44* | mitochondrial ribosomal protein L44 |  | 1.53 |  | | |
| 209708_at | NM_015529 | *moxd1* | monooxygenase, DBH-like 1 | 1.50 |  |  | | |
| 216863_s_at | NM_014941 | *zcwcc1* | MORC family CW-type zinc finger 2 |  | 1.60 |  | | |
| 212199_at | NM_152301,NM_203462 | *pp784* | Morf4 family associated protein 1-like 1 |  | 1.53 |  | | |
| 205235_s_at | NM_016195 | *mphosph1* | M-phase phosphoprotein 1 |  | 1.77 |  | | |
| 225041_at | NM_017520 | *hsmpp8* | M-phase phosphoprotein, mpp8 |  | -1.79 |  | | |
| 213543_at | None | *none* | MRNA from chromosome 5q31-33 region |  | 1.68 |  | | |
| 1566887_x_at | None | *none* | Multiple myeloma susceptibility mRNA sequence |  | -1.90 |  | | |
| 203640_at | NM_207304,NM_144778 | *mbnl2* | muscleblind-like 2 (Drosophila) |  | 1.67 |  | | |
| 201960_s_at | NM_015057 | *mycbp2* | MYC binding protein 2 |  | -1.57 |  | | |
| 224206_x_at | NM_018657 | *mynn* | myoneurin |  | 1.50 |  | | |
| 32811_at | NM_033375 | *myo1c* | myosin IC |  | -1.60 |  | | |
| 216222_s_at | NM_012334 | *myo10* | myosin X |  | -1.63 |  | | |
| 225232_at | NM_019061 | *pip3ap* | myotubularin related protein 12 | 1.55 |  |  | | |
| 223040_at | NM_181528,NM_181527,NM_016100 | *nat5* | N-acetyltransferase 5 | 1.80 |  |  | | |
| 217860_at | NM_004544 | *ndufa10* | NADH dehydrogenase (ubiquinone) 1 alpha subcomplex, 10, 42kDa | 1.50 |  |  | | |
| 223112_s_at | NM_004548 | *ndufb10* | NADH dehydrogenase (ubiquinone) 1 beta subcomplex, 10, 22kDa | 1.53 |  |  | | |
| 218697_at | NM_184231,NM_016453 | *nckipsd* | NCK interacting protein with SH3 domain |  | -1.50 |  | | |
| 1560116_a_at | None | *none* | neural precursor cell expressed, developmentally down-regulated 1 | -1.81 |  |  | | |
| 234762_x_at | None | *none* | Neurolysin (metallopeptidase M3 family) | -1.61 | -1.52 |  | | |
| 214075_at | NM_013349 | *scirp10* | neuron derived neurotrophic factor | -1.57 |  |  | | |
| 224771_at | NM_020443 | *nav1* | neuron navigator 1 |  | -2.14 |  | | |
| 202906_s_at | NM_002485 | *nbs1* | nibrin |  | 1.57 |  | | |
| 204114_at | NM_007361 | *nid2* | nidogen 2 (osteonidogen) | 1.80 |  |  | | |
| 221853_s_at | NM_014287,NM_001004060,NM_001004067,NM_173614 | *nomo1|nomo2|nomo3* | NODAL modulator 1 /// NODAL modulator 2 /// NODAL modulator 3 | 1.50 |  |  | | |
| 217802_s_at | None | *none* | nuclear casein kinase and cyclin-dependent kinase substrate 1 | -1.58 |  |  | | |
| 200056_s_at | NM_173177,NM_006333 | *c1d* | nuclear DNA-binding protein /// nuclear DNA-binding protein |  | 1.55 |  | | |
| 213298_at | NM_205843 | *nfic* | nuclear factor I/C (CCAAT-binding transcription factor) |  | -1.51 |  | | |
| 238346_s_at | NM_024831 | *ncoa6ip* | nuclear receptor coactivator 6 interacting protein |  | -1.73 |  | | |
| 209506_s_at | NM_005654 | *nr2f1* | nuclear receptor subfamily 2, group F, member 1 |  | -1.55 |  | | |
| 209119_x_at | None | *none* | nuclear receptor subfamily 2, group F, member 2 | -1.56 |  |  | | |
| 208922_s_at | NM_006362 | *nxf1* | nuclear RNA export factor 1 |  | 1.55 |  | | |
| 219978_s_at | NM_018454,NM_016359 | *nusap1* | nucleolar and spindle associated protein 1 |  | -2.35 |  | | |
| 218860_at | NM_024078 | *mgc3162* | nucleolar complex associated 4 homolog (S. cerevisiae) |  | -1.72 |  | | |
| 206323_x_at | NM_002547,NM_001011552,NM_032579,NM_020675 | *ophn1|slc9a4|retnlb|spc25(6)* | oligophrenin 1 |  | -1.71 |  | | |
| 200906_s_at | NM_016081 | *kiaa0992* | palladin, cytoskeletal associated protein |  | -1.79 |  | | |
| 221526_x_at | NM_019619 | *pard3* | par-3 partitioning defective 3 homolog (C. elegans) |  | -1.70 |  | | |
| 214175_x_at | NM_003687 | *pdlim4* | PDZ and LIM domain 4 |  | -1.62 |  | | |
| 203370_s_at | NM_203352,NM_005451,NM_203353 | *pdlim7* | PDZ and LIM domain 7 (enigma) |  | -1.54 |  | | |
| 219148_at | NM_018492 | *pbk* | PDZ binding kinase |  | 1.51 |  | | |
| 226472_at | NM_139126 | *ppil4* | peptidylprolyl isomerase (cyclophilin)-like 4 |  | 1.62 |  | | |
| 218025_s_at | NM_006117,NM_206836 | *peci* | peroxisomal D3,D2-enoyl-CoA isomerase | 1.57 |  |  | | |
| 204049_s_at | NM_014721 | *phactr2* | phosphatase and actin regulator 2 |  | 1.58 |  | | |
| 212518_at | NM_012398 | *pip5k1c* | phosphatidylinositol-4-phosphate 5-kinase, type I, gamma | -1.62 |  |  | | |
| 202430_s_at | NM_021105 | *plscr1* | phospholipid scramblase 1 |  | 1.92 |  | | |
| 218828_at | NM_020360 | *plscr3* | phospholipid scramblase 3 | -1.73 |  |  | | |
| 211668_s_at | NM_002658 | *plau* | plasminogen activator, urokinase /// plasminogen activator, urokinase | 1.52 |  |  | | |
| 202925_s_at | NM_002657 | *plagl2* | pleiomorphic adenoma gene-like 2 | 1.51 |  |  | | |
| 209466_x_at | NM_002825 | *ptn* | pleiotrophin (heparin binding growth factor 8, neurite growth-promoting factor 1) |  | -1.81 |  | | |
| 212188_at | NM_138444 | *kctd12* | potassium channel tetramerisation domain containing 12 /// potassium channel tetramerisation domain containing 12 | -1.81 | 1.63 |  | | |
| 225452_at | None | *none* | PPAR binding protein |  | -1.55 |  | | |
| 207132_x_at | NM_145896,NM_002624,NM_145897 | *pfdn5* | prefoldin subunit 5 |  | -1.79 |  | | |
| 1559400_s_at | NM_002581 | *pappa* | pregnancy-associated plasma protein A, pappalysin 1 | -2.14 | -2.66 |  | | |
| 202919_at | NM_199482,NM_015387 | *prei3* | preimplantation protein 3 |  | 1.56 |  | | |
| 226065_at | NM_153026 | *prickle1* | prickle-like 1 (Drosophila) |  | -1.64 |  | | |
| 231735_s_at | None | *none* | PRO1073 protein | -2.01 |  |  | | |
| 1564494_s_at | NM_000918 | *p4hb* | procollagen-proline, 2-oxoglutarate 4-dioxygenase (proline 4-hydroxylase), beta polypeptide | 1.50 |  |  | | |
| 200658_s_at | NM_002634 | *phb* | prohibitin | 1.54 |  |  | | |
| 200820_at | NM_002812 | *psmd8* | proteasome (prosome, macropain) 26S subunit, non-ATPase, 8 | 1.55 |  |  | | |
| 201400_at | NM_002795 | *psmb3* | proteasome (prosome, macropain) subunit, beta type, 3 | 1.62 |  |  | | |
| 228725_x_at | NM_001535,NM_206962 | *hrmt1l1* | protein arginine methyltransferase 2 | 1.56 |  |  | | |
| 208694_at | NM_006904 | *prkdc* | protein kinase, DNA-activated, catalytic polypeptide |  | 1.59 |  | | |
| 228733_at | NM_153339 | *pusl1* | pseudouridylate synthase-like 1 |  | -1.50 |  | | |
| 201251_at | NM_002654,NM_182471,NM_182470 | *pkm2* | pyruvate kinase, muscle | 1.60 |  |  | | |
| 202252_at | NM_002870 | *rab13* | RAB13, member RAS oncogene family | -1.59 |  |  | | |
| 221819_at | NM_006861 | *rab35* | RAB35, member RAS oncogene family |  | -1.52 |  | | |
| 201046_s_at | NM_005053 | *rad23a* | RAD23 homolog A (S. cerevisiae) |  | -1.63 |  | | |
| 202844_s_at | NM_006788 | *ralbp1* | ralA binding protein 1 |  | -1.76 |  | | |
| 236620_at | NM_018151 | *rif1* | RAP1 interacting factor homolog (yeast) |  | -2.00 |  | | |
| 212917_x_at | NM_002907 | *recql* | RecQ protein-like (DNA helicase Q1-like) | -1.66 |  |  | | |
| 1558924_s_at | NM_198240,NM_002956 | *rsn* | restin (Reed-Steinberg cell-expressed intermediate filament-associated protein) |  | -1.92 |  | | |
| 227852_at | NM_203288 | *rp9* | retinitis pigmentosa 9 (autosomal dominant) |  | -1.57 |  | | |
| 212331_at | NM_005611 | *rbl2* | retinoblastoma-like 2 (p130) |  | 1.57 |  | | |
| 217776_at | NM_016026 | *rdh11* | retinol dehydrogenase 11 (all-trans/9-cis/11-cis) | 1.69 |  |  | | |
| 225173_at | NM_033515 | *arhgap18* | Rho GTPase activating protein 18 |  | -1.66 |  | | |
| 223422_s_at | NM_031305 | *arhgap24* | Rho GTPase activating protein 24 |  | 1.62 |  | | |
| 204402_at | NM_012265 | *c22orf3* | rhomboid domain containing 3 | -1.52 |  |  | | |
| 212018_s_at | NM_015659 | *csig* | ribosomal L1 domain containing 1 | 1.54 |  |  | | |
| 229590_at | None | *none* | Ribosomal protein L13 | -1.85 |  |  | | |
| 219762_s_at | NM_033643,NM_015414 | *rpl36* | ribosomal protein L36 |  | -1.51 |  | | |
| 216505_x_at | NM_001014 | *rps10* | ribosomal protein S10 |  | -1.50 |  | | |
| 213350_at | None | *none* | Ribosomal protein S11 |  | -1.87 |  | | |
| 202649_x_at | NM_001022 | *rps19* | ribosomal protein S19 | -1.59 |  |  | | |
| 214097_at | NM_001024 | *rps21* | ribosomal protein S21 | -2.19 |  |  | | |
| 208904_s_at | NM_001031 | *rps28* | ribosomal protein S28 /// similar to 40S ribosomal protein S28 /// similar to 40S ribosomal protein S28 /// similar to 40S ribosomal protein S28 | -1.54 |  |  | | |
| 200858_s_at | NM_001012 | *rps8* | ribosomal protein S8 |  | -1.57 |  | | |
| 212047_s_at | NM_015528 | *rnf167* | ring finger protein 167 | -1.70 |  |  | | |
| 224395_s_at | NM_183237,NM_014245,NM_183063 | *rnf7* | ring finger protein 7 /// ring finger protein 7 | 1.64 |  |  | | |
| 212027_at | NM_021239 | *rbm25* | RNA binding motif protein 25 |  | -1.91 |  | | |
| 218379_at | NM_016090 | *rbm7* | RNA binding motif protein 7 | 1.53 | 2.21 |  | | |
| 207836_s_at | NM_001008710,NM_001008711,NM_006867 | *rbpms* | RNA binding protein with multiple splicing |  | -1.56 |  | | |
| 228602_at | NM_000337 | *sgcd* | sarcoglycan, delta (35kDa dystrophin-associated glycoprotein) | -1.56 |  |  | | |
| 202798_at | NM_006323 | *sec24b* | SEC24 related gene family, member B (S. cerevisiae) | 1.64 |  |  | | |
| 233168_s_at | NM_031454 | *selo* | selenoprotein O |  | -1.66 |  | | |
| 35666_at | NM_004186 | *sema3f* | sema domain, immunoglobulin domain (Ig), short basic domain, secreted, (semaphorin) 3F |  | -1.63 |  | | |
| 208610_s_at | NM_016333 | *srrm2* | serine/arginine repetitive matrix 2 |  | -2.48 |  | | |
| 207714_s_at | NM_001235 | *serpinh1* | serpin peptidase inhibitor, clade H (heat shock protein 47), member 1, (collagen binding protein 1) | 1.61 |  |  | | |
| 223195_s_at | NM_031459 | *sesn2* | sestrin 2 |  | -1.56 |  | | |
| 225850_at | NM_145169 | *c6orf83* | SFT2 domain containing 1 | 1.53 |  |  | | |
| 215499_at | None | *none* | similar to mitogen-activated protein kinase kinase 3 isoform A /// similar to mitogen-activated protein kinase kinase 3 isoform A | -1.53 |  |  | | |
| 222207_x_at | NM_017741,NM_133646 | *flj20280|zak* | similar to Williams Beuren syndrome chromosome region 19 /// similar to Williams Beuren syndrome chromosome region 19 /// region containing similar to Williams Beuren syndrome chromosome region 19; hypothetical LOC441257 /// region containing similar to Williams Beuren syndrome chromosome region 19; hypothetical LOC441257 /// region containing similar to Williams Beuren syndrome chromosome region 19; hypothetical LOC441257 /// region containing similar to Williams Beuren syndrome chromosome region 19; hypothetical LOC441257 | -1.57 |  |  | | |
| 201663_s_at | NM_001002799,NM_001002800,NM_005496 | *smc4l1* | SMC4 structural maintenance of chromosomes 4-like 1 (yeast) | 1.64 | 1.74 |  | | |
| 215424_s_at | NM_012245 | *skiip* | SNW domain containing 1 |  | 1.52 |  | | |
| 220371_s_at | NM_020246 | *slc12a9* | solute carrier family 12 (potassium/chloride transporters), member 9 |  | -1.50 |  | | |
| 203340_s_at | NM_003705 | *slc25a12* | solute carrier family 25 (mitochondrial carrier, Aralar), member 12 |  | 1.52 |  | | |
| 201085_s_at | NM_138925,NM_003103,NM_032195,NM_138927 | *son* | SON DNA binding protein |  | -1.53 |  | | |
| 224818_at | NM_002959 | *sort1* | sortilin 1 | 1.89 | -1.65 |  | | |
| 208781_x_at | NM_003795,NM_152827,NM_152828 | *snx3* | sorting nexin 3 |  | 1.89 |  | | |
| 223028_s_at | NM_016224 | *snx9* | sorting nexin 9 |  | -1.56 |  | | |
| 208611_s_at | NM_003127 | *sptan1* | spectrin, alpha, non-erythrocytic 1 (alpha-fodrin) |  | 1.66 |  | | |
| 212468_at | None | *none* | sperm associated antigen 9 |  | -2.15 |  | | |
| 201586_s_at | NM_005066 | *sfpq* | splicing factor proline/glutamine-rich (polypyrimidine tract binding protein associated) | 1.75 |  |  | | |
| 213850_s_at | NM_004719 | *sfrs2ip* | splicing factor, arginine/serine-rich 2, interacting protein |  | -1.61 |  | | |
| 214141_x_at | NM_006276 | *sfrs7* | splicing factor, arginine/serine-rich 7, 35kDa | 1.66 |  |  | | |
| 213577_at | None | *none* | squalene epoxidase |  | -1.66 |  | | |
| 224130_s_at | XR_000132 | *none* | steroid receptor RNA activator 1 |  | 1.50 |  | | |
| 202026_at | NM_003002 | *sdhd* | succinate dehydrogenase complex, subunit D, integral membrane protein |  | 1.53 |  | | |
| 214835_s_at | NM_003848 | *suclg2* | succinate-CoA ligase, GDP-forming, beta subunit | 1.62 |  |  | | |
| 217995_at | NM_021199 | *sqrdl* | sulfide quinone reductase-like (yeast) | 1.61 |  |  | | |
| 212520_s_at | NM_003072 | *smarca4* | SWI/SNF related, matrix associated, actin dependent regulator of chromatin, subfamily a, member 4 |  | -1.68 |  | | |
| 228397_at | None | *none* | taurine upregulated gene 1 |  | 1.78 |  | | |
| 224622_at | NM_020773 | *tbc1d14* | TBC1 domain family, member 14 |  | 1.50 |  | | |
| 204158_s_at | NM_006053,NM_006019 | *tcirg1* | T-cell, immune regulator 1, ATPase, H+ transporting, lysosomal V0 subunit A3 |  | -1.66 |  | | |
| 225387_at | None | *none* | tetraspanin 5 | 1.60 |  |  | | |
| 220607_x_at | NM_016397,NM_198976 | *th1l* | TH1-like (Drosophila) |  | 1.51 |  | | |
| 227611_at | NM_152334 | *tarsl2* | threonyl-tRNA synthetase-like 2 | 1.63 |  |  | | |
| 47608_at | NM_080604 | *tjp4* | tight junction associated protein 1 (peripheral) |  | -1.68 |  | | |
| 208900_s_at | NM_003286 | *top1* | topoisomerase (DNA) I |  | -2.05 |  | | |
| 229128_s_at | None | *none* | Transcribed locus |  | 1.55 |  | | |
| 202818_s_at | NM_003198 | *tceb3* | transcription elongation factor B (SIII), polypeptide 3 (110kDa, elongin A) |  | -1.97 |  | | |
| 213311_s_at | NM_014972 | *kiaa1049* | transcription factor 25 (basic helix-loop-helix) |  | -1.93 |  | | |
| 212758_s_at | NM_030751 | *tcf8* | transcription factor 8 (represses interleukin 2 expression) |  | -1.69 |  | | |
| 222633_at | NM_024665 | *tbl1xr1* | transducin (beta)-like 1X-linked receptor 1 |  | -2.14 |  | | |
| 208700_s_at | NM_001064 | *tkt* | transketolase (Wernicke-Korsakoff syndrome) | 1.57 |  |  | | |
| 226529_at | NM_018374 | *flj11273* | transmembrane protein 106B |  | 1.52 |  | | |
| 223113_at | NM_016464 | *hspc196* | transmembrane protein 138 | 1.71 |  |  | | |
| 212989_at | NM_147156 | *tmem23* | transmembrane protein 23 |  | 1.58 |  | | |
| 204808_s_at | NM_014254 | *tmem5* | transmembrane protein 5 |  | -1.51 |  | | |
| 229036_at | None | *none* | trinucleotide repeat containing 6B |  | -1.72 |  | | |
| 213027_at | None | *none* | TROVE domain family, member 2 |  | 1.63 |  | | |
| 218156_s_at | NM_018128 | *flj10534* | TSR1, 20S rRNA accumulation, homolog (S. cerevisiae) | 1.84 |  |  | | |
| 213476_x_at | NM_001069,NM_006086,NM_178012,NM_006087 | *tubb2|tubb3|mgc8685|tubb4(6)* | tubulin, beta 3 | 1.75 |  |  | | |
| 200638_s_at | NM_003406,NM_145690 | *ywhaz* | tyrosine 3-monooxygenase/tryptophan 5-monooxygenase activation protein, zeta polypeptide |  | 1.50 |  | | |
| 202858_at | NM_006758 | *u2af1* | U2 small nuclear RNA auxiliary factor 1 | 1.77 |  |  | | |
| 209088_s_at | NM_016936 | *ubn1* | ubinuclein 1 |  | -1.94 |  | | |
| 201903_at | NM_003365 | *uqcrc1* | ubiquinol-cytochrome c reductase core protein I | 1.55 |  |  | | |
| 200883_at | NM_003366 | *uqcrc2* | ubiquinol-cytochrome c reductase core protein II | 1.83 |  |  | | |
| 221839_s_at | NM_020867,NM_148171,NM_018449 | *ubap2* | ubiquitin associated protein 2 |  | -1.53 |  | | |
| 222616_s_at | NM_001001992,NM_006447 | *usp16* | ubiquitin specific peptidase 16 |  | -2.49 |  | | |
| 212388_at | None | *none* | ubiquitin specific peptidase 24 |  | 1.60 |  | | |
| 226176_s_at | None | *none* | ubiquitin specific peptidase 42 | -1.58 | -1.63 |  | | |
| 209115_at | NM_198197,NM_198195,NM_003968 | *ube1c* | ubiquitin-activating enzyme E1C (UBA3 homolog, yeast) |  | 1.54 |  | | |
| 223229_at | NM_014176 | *hspc150* | ubiquitin-conjugating enzyme E2T (putative) |  | 1.65 |  | | |
| 218801_at | NM_020121 | *ugcgl2* | UDP-glucose ceramide glucosyltransferase-like 2 |  | 1.56 |  | | |
| 1568618_a_at | NM_020474 | *galnt1* | UDP-N-acetyl-alpha-D-galactosamine:polypeptide N-acetylgalactosaminyltransferase 1 (GalNAc-T1) | -1.58 |  |  | | |
| 230306_at | NM_052875 | *mgc10485* | vacuolar protein sorting 26 homolog B (S. cerevisiae) | -1.59 |  |  | | |
| 217913_at | NM_013245 | *vps4a* | vacuolar protein sorting 4 homolog A (S. cerevisiae) |  | -1.61 |  | | |
| 202205_at | NM_001008736,NM_003370 | *vasp* | vasodilator-stimulated phosphoprotein |  | -1.84 |  | | |
| 217234_s_at | NM_003379 | *vil2* | villin 2 (ezrin) |  | -2.01 |  | | |
| 202431_s_at | NM_002467 | *myc* | v-myc myelocytomatosis viral oncogene homolog (avian) |  | 1.53 |  | | |
| 212038_s_at | NM_003374 | *vdac1* | voltage-dependent anion channel 1 | 1.56 |  |  | | |
| 224730_at | NM_005828 | *han11* | WD repeat domain 68 |  | -1.93 |  | | |
| 206621_s_at | NM_031992,NM_022170 | *wbscr1* | Williams-Beuren syndrome chromosome region 1 | 1.50 |  |  | | |
| 205809_s_at | NM_003941 | *wasl* | Wiskott-Aldrich syndrome-like |  | -1.71 |  | | |
| 203599_s_at | NM_007187 | *wbp4* | WW domain binding protein 4 (formin binding protein 21) |  | 1.56 |  | | |
| 200670_at | NM_005080 | *xbp1* | X-box binding protein 1 | 1.63 |  |  | | |
| 201901_s_at | NM_003403 | *yy1* | YY1 transcription factor |  | -1.92 |  | | |
| 233296_x_at | None | *none* | Zinc finger and BTB domain containing 20 |  | -1.63 |  | | |
| 219221_at | None | *none* | zinc finger and BTB domain containing 38 |  | 1.57 |  | | |
| 200829_x_at | NM_003457 | *znf207* | zinc finger protein 207 | -1.54 |  |  | | |
| 212368_at | None | *none* | zinc finger protein 292 |  | 1.50 |  | | |
| 215596_s_at | NM_015565 | *znf294* | zinc finger protein 294 |  | 1.51 |  | | |
| 200867_at | NM_018683 | *znf313* | zinc finger protein 313 | 1.51 |  |  | | |
| 233936_s_at | NM_024835 | *znf403* | zinc finger protein 403 |  | -1.58 |  | | |
| 219981_x_at | None | *none* | Zinc finger protein 587 | -1.50 |  |  | | |
| 234982_at | NM_172070 | *znf650* | zinc finger protein 650 |  | 1.76 |  | | |
