# Supplementary material for: An approach to analyse the specific impact of rapamycin on mRNA-ribosome association
Source: BMC Med Genomics. 2008 Aug 1;1:33. doi: 10.1186/1755-8794-1-33 (PMC2533349; doi:10.1186/1755-8794-1-33)
Supplement: Additional File 2 — Complete list of polysomal transcripts regulated by rapamycin. After GCOS normalisation a ×2.5 fold selection cut-off was applied to all the regulated transcripts independent of probe set intensity values. [file 1755-8794-1-33-S2.doc]

| **Additional Table 2 : Polysomal transcripts regulated by rapamycin** | | | |  |  |
| --- | --- | --- | --- | --- | --- |
|  |  |  |  |  |  |
|  |  |  |  | **Fold change** |  |
| **Affimetrix probe** | **Id** | **Gene symbol** | **Gene description** | **Light** | **Heavy** |
|  |  |  |  |  |  |
| 214826_at | U79276 | *2'-pde* | 2'-phosphodiesterase | -4.270 |  |
| 1553605_a_at | NM_152701 | *abca13* | ATP-binding cassette, sub-family A (ABC1), member 13 | 2.525 | 4.202 |
| 235578_at | BF247374 | *abcc9* | ATP-binding cassette, sub-family C (CFTR/MRP), member 9 | -4.697 |  |
| 207583_at | NM_005164 | *abcd2* | ATP-binding cassette, sub-family D (ALD), member 2 | -5.205 |  |
| 207593_at | NM_022169 | *abcg4* | ATP-binding cassette, sub-family G (WHITE), member 4 |  | -3.611 |
| 227631_at | BF058849 | *abi2* | Abl interactor 2 |  | -3.537 |
| 1559077_at | AL833204 | *abi3bp* | ABI gene family, member 3 (NESH) binding protein | -5.438 |  |
| 200965_s_at | NM_006720 | *ablim1* | actin binding LIM protein 1 | 3.460 |  |
| 214763_at | AK023937 | *acot11* | acyl-CoA thioesterase 11 | 2.710 |  |
| 206776_x_at | NM_001612 | *acrv1* | acrosomal vesicle protein 1 | -2.818 |  |
| 234483_at | AK024424 | *acss1* | acyl-CoA synthetase short-chain family member 1 | -2.611 |  |
| 215787_at | AK025094 | *acta2* | Actin, alpha 2, smooth muscle, aorta | -3.881 |  |
| 203864_s_at | NM_001103 | *actn2* | actinin, alpha 2 |  | -2.752 |
| 208223_s_at | NM_020327 | *acvr1b* | activin A receptor, type IB | -3.431 |  |
| 234131_at | AF090948 | *adam10* | ADAM metallopeptidase domain 10 | -3.895 |  |
| 1562137_at | AF147388 | *adam10* | ADAM metallopeptidase domain 10 |  | -3.883 |
| 204943_at | NM_021641 | *adam12* | ADAM metallopeptidase domain 12 (meltrin alpha) |  | 5.236 |
| 1555024_at | BC036029 | *adam22* | ADAM metallopeptidase domain 22 | -4.785 |  |
| 232570_s_at | AL356755 | *adam33* | ADAM metallopeptidase domain 33 |  | -3.290 |
| 1555326_a_at | AF495383 | *adam9* | ADAM metallopeptidase domain 9 (meltrin gamma) |  | 2.646 |
| 221421_s_at | NM_030955 | *adamts12* | ADAM metallopeptidase with thrombospondin type 1 motif, 12 |  | -4.093 |
| 220717_at | NM_025003 | *adamts20* | ADAM metallopeptidase with thrombospondin type 1 motif, 20 | -4.230 |  |
| 1558636_s_at | BI254089 | *adamts5* | ADAM metallopeptidase with thrombospondin type 1 motif, 5 (aggrecanase-2) | -2.702 |  |
| 228911_at | AW007289 | *adamts7* | ADAM metallopeptidase with thrombospondin type 1 motif, 7 |  | -3.291 |
| 217687_at | AA224446 | *adcy2* | adenylate cyclase 2 (brain) |  | -2.955 |
| 207544_s_at | NM_000672 | *adh6* | alcohol dehydrogenase 6 (class V) |  | -3.630 |
| 238702_at | BG287503 | *admp* | likely ortholog of androgen down regulated gene expressed in mouse prostate |  | -2.732 |
| 205013_s_at | NM_000675 | *adora2a* | adenosine A2a receptor |  | -3.349 |
| 211489_at | D32201 | *adra1a* | adrenergic, alpha-1A-, receptor | -3.959 |  |
| 206128_at | AI264306 | *adra2c* | adrenergic, alpha-2C-, receptor | -3.533 |  |
| 233556_at | BC004918 | *adrbk2* | adrenergic, beta, receptor kinase 2 |  | -2.585 |
| 1555435_at | BC025700 | *aff4* | AF4/FMR2 family, member 4/MCEF/AF5Q31 | 4.329 |  |
| 232865_at | N59653 | *aff4* | AF4/FMR2 family, member 4 /ALL1 fused |  | -5.055 |
| 219693_at | NM_020133 | *agpat4* | 1-acylglycerol-3-phosphate O-acyltransferase 4 (lysophosphatidic acid acyltransferase, delta) | -3.396 |  |
| 220842_at | NM_017651 | *ahi1* | Abelson helper integration site | -2.888 |  |
| 1556212_x_at | N62827 | *aig1* | Androgen-induced 1 | -6.089 | -3.565 |
| 206460_at | NM_018836 | *ajap1* | adherens junction associated protein 1 |  | -3.037 |
| 243450_at | T40707 | *akap13* | A kinase (PRKA) anchor protein 13 | -2.896 |  |
| 218064_s_at | NM_014371 | *akap8l* | A kinase (PRKA) anchor protein 8-like | 3.817 |  |
| 244393_x_at | AW152368 | *akr1cl2* | Aldo-keto reductase family 1, member C-like 2 | -2.855 |  |
| 1552585_s_at | NM_172196 | *alf* | TFIIA-alpha/beta-like factor | -3.818 |  |
| 235380_at | AA768919 | *alox5* | Arachidonate 5-lipoxygenase | -5.282 |  |
| 227438_at | AI760166 | *alpk1* | alpha-kinase 1 |  | 8.130 |
| 211619_s_at | M13077 | *alpp ; alppl2* | alkaline phosphatase, placental (Regan isozyme) ; alkaline phosphatase, placental-like 2 | -2.539 |  |
| 1556924_at | AB053312 | *als2cr10* | amyotrophic lateral sclerosis 2 (juvenile) chromosome region, candidate 10 | -3.370 |  |
| 1554831_x_at | BC030659 | *als2cr11* | amyotrophic lateral sclerosis 2 (juvenile) chromosome region, candidate 11 | -5.534 |  |
| 239192_at | BF434006 | *als2cr19* | amyotrophic lateral sclerosis 2 (juvenile) chromosome region, candidate 19 |  | 4.762 |
| 208220_x_at | NM_001143 | *amely* | amelogenin, Y-linked | -3.698 |  |
| 226718_at | AA001423 | *amigo1* | adhesion molecule with Ig-like domain 1 |  | -5.050 |
| 217630_at | AI188346 | *angel2* | angel homolog 2 (Drosophila) |  | -2.920 |
| 1552939_at | NM_139290 | *angpt1* | angiopoietin 1 |  | -4.769 |
| 206385_s_at | NM_020987 | *ank3* | ankyrin 3, node of Ranvier (ankyrin G) |  | 2.857 |
| 1560369_at | AL833238 | *ankh* | Ankylosis, progressive homolog (mouse) | -4.096 |  |
| 1560370_x_at | AL833238 | *ankh* | Ankylosis, progressive homolog (mouse) | -3.172 |  |
| 1556542_a_at | AF086077 | *ankmy2* | Ankyrin repeat and MYND domain containing 2 | -5.749 |  |
| 1569578_at | BC028913 | *ankrd11* | Ankyrin repeat domain 11 |  | 2.924 |
| 1556361_s_at | BC016937 | *ankrd13c* | ankyrin repeat domain 13C |  | 3.802 |
| 222329_x_at | AW974816 | *ankrd17* | Ankyrin repeat domain 17 | -2.956 |  |
| 241321_at | AI379751 | *ankrd23* | Ankyrin repeat domain 23 | 6.024 |  |
| 1558398_at | AB075861 | *ankrd24* | ankyrin repeat domain 24 |  | -3.666 |
| 1562294_x_at | AF269088 | *ankrd30b* | ankyrin repeat domain 30B |  | -4.459 |
| 229125_at | AA456955 | *ankrd38* | ankyrin repeat domain 38 | -7.790 |  |
| 235954_at | AA972597 | *anp32a* | Acidic (leucine-rich) nuclear phosphoprotein 32 family, member A |  | -2.518 |
| 241294_at | AI939422 | *ap1g1* | Adaptor-related protein complex 1, gamma 1 subunit | -3.549 |  |
| 1555421_at | BC009606 | *ap1s3* | adaptor-related protein complex 1, sigma 3 subunit | 2.933 |  |
| 230925_at | AI093231 | *apbb1ip* | amyloid beta (A4) precursor protein-binding, family B, member 1 interacting protein |  | -2.713 |
| *237327_at* | *BE220031* | *aph1a* | *anterior pharynx defective 1 homolog A (C. elegans)* | *2.100* |  |
| 237327_at | BE220031 | *aph1a* | anterior pharynx defective 1 homolog A (C. elegans) |  | 5.208 |
| 220023_at | NM_018690 | *apob48r* | apolipoprotein B48 receptor | -4.001 |  |
| 1553831_at | NM_152426 | *apobec3d* | apolipoprotein B mRNA editing enzyme, catalytic polypeptide-like 3D |  | -2.753 |
| 214995_s_at | BF508948 | *apobec3* | apolipoprotein B mRNA editing enzyme, catalytic polypeptide-like 3G ; apolipoprotein B mRNA editing enzyme, catalytic polypeptide-like 3F |  | 4.292 |
| 214910_s_at | AF161454 | *apom* | apolipoprotein M |  | 3.030 |
| 243314_at | W90446 | *app* | Amyloid beta (A4) precursor protein (peptidase nexin-II, Alzheimer disease) |  | -3.303 |
| 241701_at | BF369489 | *arhgap21* | Rho GTPase activating protein 21 | -3.466 |  |
| 1555076_at | BC039591 | *arhgap25* | Rho GTPase activating protein 25 |  | -3.176 |
| 227911_at | AI935647 | *arhgap28* | Rho GTPase activating protein 28 | 2.890 |  |
| 235412_at | ?AI040887 | *arhgef7* | Rho guanine nucleotide exchange factor (GEF) 7 | 5.376 |  |
| 1562270_at | AL831814 | *arhgef7* | Rho guanine nucleotide exchange factor (GEF) 7 |  | -6.309 |
| 1556818_at | BE671639 | *arid1b* | AT rich interactive domain 1B (SWI1-like) |  | 6.061 |
| 241837_at | AI289774 | *arid5b* | AT rich interactive domain 5B (MRF1-like) | 4.049 |  |
| 1559121_s_at | AI767566 | *arih2* | Ariadne homolog 2 (Drosophila) | -2.875 |  |
| 202091_at | BC003087 | *arl2bp* | ADP-ribosylation factor-like 2 binding protein | -2.631 |  |
| 213433_at | AF038193 | *arl3* | ADP-ribosylation factor-like 3 |  | -3.069 |
| 219094_at | NM_014154 | *armc8* | armadillo repeat containing 8 |  | 2.551 |
| 236966_at | BF942281 | *armc8* | armadillo repeat containing 8 |  | 4.292 |
| 1564399_a_at | AK096955 | *armcx4* | armadillo repeat containing, X-linked 4 | -4.378 |  |
| 1557726_at | BC036206 | *armcx4* | armadillo repeat containing, X-linked 4 | -2.589 |  |
| 1552722_at | NM_016300 | *arpp-21* | cyclic AMP-regulated phosphoprotein, 21 kD | -12.380 |  |
| 1557626_at | CA444630 | *arrb1* | arrestin, beta 1 |  | 2.809 |
| 231791_at | AL047141 | *asah2* | N-acylsphingosine amidohydrolase (non-lysosomal ceramidase) 2 | -2.979 |  |
| 231622_at | AI220527 | *asb17* | ankyrin repeat and SOCS box-containing 17 |  | -6.037 |
| 217114_at | Z70200 | *ascc3l1* | activating signal cointegrator 1 complex subunit 3-like 1 | 2.841 |  |
| 232931_at | AK021583 | *ascc3l1* | Activating signal cointegrator 1 complex subunit 3-like 1 |  | 3.817 |
| 231069_at | AI768895 | *ash2l* | ash2 (absent, small, or homeotic)-like (drosophila) | 7.463 |  |
| 216132_at | AK021992 | *astn2* | Astrotactin 2 |  | 5.848 |
| 240072_at | N75937 | *asxl2* | additional sex combs like 2 (Drosophila) |  | -2.556 |
| 208033_s_at | NM_006885 | *atbf1* | AT-binding transcription factor 1 | -5.355 |  |
| 243477_at | AI027990 | *atg10* | ATG10 autophagy related 10 homolog (S. cerevisiae) |  | 4.525 |
| 243585_at | AW444437 | *atp13a5* | ATPase type 13A5 |  | -4.197 |
| 215911_x_at | AW615612 | *atp2b3/pmca3* | ATPase, Ca++ transporting, plasma membrane 3 | -3.955 |  |
| 201442_s_at | AF248966 | *atp6ap2* | ATPase, H+ transporting, lysosomal accessory protein 2 | -4.542 |  |
| 215319_at | AC004755 | *atp8b3* | ATPase, Class I, type 8B, member 3 | -4.913 |  |
| 215319_at | AC004755 | *atp8b3* | ATPase, Class I, type 8B, member 3 |  | 4.902 |
| 236762_at | BE818251 | *atp9b* | ATPase, Class II, type 9B | 2.890 |  |
| 211022_s_at | BC002521 | *atrx* | alpha thalassemia/mental retardation syndrome X-linked (RAD54 homolog, S. cerevisiae) |  | -3.836 |
| 236404_at | AW197320 | *atxn1* | Ataxin 1 | 2.653 |  |
| 233182_x_at | AU146105 | *atxn3* | Ataxin 3 | -3.455 |  |
| 237330_at | AA603494 | *avo3* | TORC2-specific protein AVO3 | -4.368 |  |
| 209309_at | D90427 | *azgp1* | alpha-2-glycoprotein 1, zinc |  | 2.950 |
| 240231_at | AI742383 | *azin* | Antizyme inhibitor 1 |  | 2.710 |
| 1561714_a_at | BC016974 | *b3galnt2* | UDP-GalNAc:betaGlcNAc beta 1,3-galactosaminyltransferase, polypeptide 2 |  | 2.874 |
| 232337_at | AK000770 | *b3gnt7* | UDP-GlcNAc:betaGal beta-1,3-N-acetylglucosaminyltransferase 7 |  | -4.954 |
| 1566087_at | AL833356 | *b3gtl* | Beta 1,3-galactosyltransferase-like | 3.571 |  |
| 206233_at | AF097159 | *b4galt6* | UDP-Gal:betaGlcNAc beta 1,4- galactosyltransferase, polypeptide 6 |  | 6.250 |
| 1555603_at | AF527551 | *bage* | B melanoma antigen | 3.559 |  |
| *213336_at* | *AI826454* | *baz1b* | *Bromodomain adjacent to zinc finger domain, 1B* |  | *-2.200* |
| 241640_at | BG149769 | *bcap29* | B-cell receptor-associated protein 29 | -7.594 |  |
| 220488_s_at | NM_017679 | *bcas3* | breast carcinoma amplified sequence 3 |  | -3.071 |
| 219497_s_at | NM_022893 | *bcl11a* | B-cell CLL/lymphoma 11A (zinc finger protein) | -2.586 |  |
| 206665_s_at | NM_001191 | *bcl2l1* | BCL2-like 1 | -3.287 |  |
| 1560977_a_at | W74646 | *bcl2l13* | BCL2-like 13 (apoptosis facilitator) | -2.898 | -2.959 |
| 236143_at | BF433037 | *bdh* | 3-hydroxybutyrate dehydrogenase (heart, mitochondrial) | -3.817 |  |
| 1567361_at | AJ011597 | *bdnfos* | brain-derived neurotrophic factor opposite strand |  | -2.775 |
| 229437_at | BG231961 | *bic* | BIC transcript | 5.181 |  |
| 240461_at | R88483 | *bicd1* | Bicaudal D homolog 1 (Drosophila) | -6.260 |  |
| 209203_s_at | BC002327 | *bicd2* | bicaudal D homolog 2 (Drosophila) |  | -3.668 |
| 242234_at | AI859280 | *birc4bp* | XIAP associated factor-1 |  | -2.932 |
| 242068_at | AA608834 | *birc6* | Baculoviral IAP repeat-containing 6 (apollon) |  | -9.642 |
| 207655_s_at | NM_013314 | *blnk* | B-cell linker |  | 4.255 |
| 203771_s_at | AA740186 | *blvra* | biliverdin reductase A | 5.814 |  |
| 203772_at | U34877 | *blvra* | biliverdin reductase A | -3.367 |  |
| 239032_at | BF513944 | *blzf1* | Basic leucine zipper nuclear factor 1 (JEM-1) | -3.185 |  |
| 1555547_at | AB088847 | *bpa1* | BPA-1 mRNA for brain peptide A1 | -4.825 |  |
| 1568768_s_at | AW080339 | *bre* | brain and reproductive organ-expressed (TNFRSF1A modulator) |  | 2.646 |
| 231960_at | AA807344 | *brwd1* | bromodomain and WD repeat domain containing 1 | -2.797 |  |
| 243509_at | AI475680 | *btg1* | B-cell translocation gene 1, anti-proliferative |  | 3.521 |
| 238596_at | BG530058 | *c10orf4* | chromosome 10 open reading frame 4 | 4.975 |  |
| 238989_at | BF749723 | *c1galt1c1* | C1GALT1-specific chaperone 1 | -3.652 |  |
| 225400_at | BF111780 | *c1orf19* | chromosome 1 open reading frame 19 |  | -6.263 |
| 1560418_at | AK027189 | *c6orf182* | Chromosome 6 open reading frame 182 |  | -5.444 |
| 209821_at | AB024518 | *c9orf26* | chromosome 9 open reading frame 26 (NF-HEV) |  | -4.143 |
| 236518_at | BE208843 | *c9orf86* | Chromosome 9 open reading frame 86 | -2.657 |  |
| 1559419_at | AL162054 | *cacnb2* | calcium channel, voltage-dependent, beta 2 subunit | -5.559 |  |
| 1555098_a_at | AF465485 | *cacnb2* | calcium channel, voltage-dependent, beta 2 subunit | -4.872 |  |
| 1552602_at | NM_145811 | *cacng5* | calcium channel, voltage-dependent, gamma subunit 5 | -2.708 |  |
| 233950_at | AK000873 | *cadps* | Ca2+-dependent secretion activator | -3.328 |  |
| 215198_s_at | AU147402 | *cald1* | caldesmon 1 |  | 3.165 |
| 1555168_a_at | BC020200 | *caln1* | calneuron 1 | -3.241 |  |
| 240593_x_at | R98767 | *camk2d* | Calcium/calmodulin-dependent protein kinase (CaM kinase) II delta |  | 5.102 |
| 224370_s_at | AF251056 | *caps2* | calcyphosine 2 | -3.732 |  |
| 206339_at | NM_004291 | *cart* | cocaine- and amphetamine-regulated transcript | -3.750 |  |
| 1564010_at | AK026822 | *cast* | Calpastatin |  | -11.250 |
| 238363_at | AW015521 | *cat* | Catalase | -3.849 |  |
| 230981_at | AI219834 | *catsper3* | cation channel, sperm associated 3 | -2.842 |  |
| 1555920_at | BU683892 | *cbx3* | Chromobox homolog 3 (HP1 gamma homolog, Drosophila) | -2.668 |  |
| 236566_at | AI291189 | *ccar1* | Cell division cycle and apoptosis regulator 1 |  | -3.221 |
| 230327_at | AI203673 | *ccl27* | chemokine (C-C motif) ligand 27 |  | -3.605 |
| 241495_at | AI675298 | *ccnl1* | Cyclin L1 | -2.836 |  |
| 204645_at | NM_001241 | *ccnt2* | cyclin T2 |  | 3.175 |
| 220565_at | NM_016602 | *ccr10* | chemokine (C-C motif) receptor 10 |  | -3.561 |
| 244812_at | AA758116 | *ccrk* | Cell cycle related kinase | -4.005 |  |
| 1557810_at | BM352108 | *cct5* | Chaperonin containing TCP1, subunit 5 (epsilon) | -6.372 |  |
| 210436_at | BC005220 | *cct8* | chaperonin containing TCP1, subunit 8 (theta) | 3.155 | -3.574 |
| 206545_at | NM_006139 | *cd28* | CD28 antigen (Tp44) | -2.695 |  |
| 1555636_at | AF427619 | *cd300lg* | CD300 antigen like family member G |  | -2.631 |
| 228766_at | AW299226 | *cd36* | CD36 antigen (collagen type I receptor, thrombospondin receptor) |  | -3.584 |
| 203547_at | U47924 | *cd4* | CD4 antigen (p55) ; CD4 antigen (p55) | -3.317 |  |
| 1565868_at | W96225 | *cd44* | CD44 antigen (homing function and Indian blood group system) | -2.620 |  |
| 239044_at | BF696395 | *cda08* | T-cell immunomodulatory protein |  | -3.662 |
| 207319_s_at | NM_003718 | *cdc2l5* | cell division cycle 2-like 5 (cholinesterase-related cell division controller) |  | 4.065 |
| 1556931_at | AF086337 | *cdc42* | Cell division cycle 42 (GTP binding protein, 25kDa) | -7.891 |  |
| 214464_at | NM_003607 | *cdc42bpa* | CDC42 binding protein kinase alpha (DMPK-like) |  | -2.538 |
| 209055_s_at | AW268817 | *cdc5l* | CDC5 cell division cycle 5-like (S. pombe) | -3.963 |  |
| 230060_at | AI277642 | *cdca7* | cell division cycle associated 7 |  | -4.959 |
| 207143_at | NM_001259 | *cdk6* | cyclin-dependent kinase 6 | -4.409 | 3.333 |
| 236023_at | ?AI703465 | *cdk9* | cyclin-dependent kinase 9 (CDC2-related kinase) |  | -3.460 |
| 228861_at | BE046983 | *cds2* | CDP-diacylglycerol synthase (phosphatidate cytidylyltransferase) 2 | 2.639 |  |
| 233630_at | AK027196 | *cds2* | CDP-diacylglycerol synthase (phosphatidate cytidylyltransferase) 2 |  | -3.087 |
| 240594_at | W86659 | *cdyl* | Chromodomain protein, Y-like | -4.221 |  |
| 206198_s_at | L31792 | *ceacam7* | carcinoembryonic antigen-related cell adhesion molecule 7 | 3.311 |  |
| 206199_at | NM_006890 | *ceacam7* | carcinoembryonic antigen-related cell adhesion molecule 7 | -4.247 | *-2.400* |
| 207331_at | NM_016343 | *cenpf* | centromere protein F, 350/400ka (mitosin) | -5.277 | -3.524 |
| 219150_s_at | NM_006869 | *centa1* | centaurin, alpha 1 |  | -2.686 |
| 205642_at | NM_007018 | *cep1* | centrosomal protein 1 |  | 2.755 |
| 239797_at | AA503877 | *cept1* | Choline/ethanolamine phosphotransferase 1 |  | -5.541 |
| 244443_at | BE247450 | *chd2* | Chromodomain helicase DNA binding protein 2 | -4.563 |  |
| 230156_x_at | AI933049 | *chd2* | chromodomain helicase DNA binding protein 2 |  | 5.747 |
| 220586_at | NM_025134 | *chd9* | chromodomain helicase DNA binding protein 9 | -5.886 |  |
| 221355_at | NM_005199 | *chrng* | cholinergic receptor, nicotinic, gamma polypeptide |  | -2.791 |
| 221164_x_at | NM_012126 | *chst5* | carbohydrate (N-acetylglucosamine 6-O) sulfotransferase 5 |  | 4.405 |
| 206164_at | NM_006536 | *clca2* | chloride channel, calcium activated, family member 2 | -2.945 | -2.895 |
| 206165_s_at | NM_006536 | *clca2* | chloride channel, calcium activated, family member 2 |  | -2.764 |
| 1554749_s_at | BC020873 | *clcnkb* | chloride channel Kb | -3.143 | 3.378 |
| 214598_at | AL049977 | *cldn8* | claudin 8 |  | -9.861 |
| 220496_at | NM_016509 | *clec1b* | C-type lectin domain family 1, member B | -3.806 |  |
| 219341_at | NM_018941 | *cln8* | ceroid-lipofuscinosis, neuronal 8 (epilepsy, progressive with mental retardation) |  | -3.184 |
| 230657_at | AI423466 | *clock* | Clock homolog (mouse) |  | -2.596 |
| 216296_at | X81636 | *clta* | clathrin, light polypeptide (Lca) | -3.157 | -2.840 |
| 210498_at | AF130062 | *cltc* | clathrin, heavy polypeptide (Hc) |  | -3.383 |
| 1565657_at | BC042995 | *cmtm6* | CKLF-like MARVEL transmembrane domain containing 6 | -3.097 |  |
| 1569780_at | BC026117 | *cntnap2* | Contactin associated protein-like 2 | -7.452 | -2.514 |
| 219301_s_at | AU144598 | *cntnap2* | contactin associated protein-like 2 |  | -2.984 |
| 232388_at | AB051550 | *cntnap4* | contactin associated protein-like 4 | 2.703 |  |
| 214200_s_at | AI193744 | *col6a1* | Collagen, type VI, alpha 1 |  | -3.642 |
| 236131_at | AW452631 | *copg2* | Coatomer protein complex, subunit gamma 2 | -4.158 |  |
| 216799_at | AL442084 | *coro2b* | Coronin, actin binding protein, 2B |  | -4.711 |
| 239760_at | AI198212 | *cox11* | COX11 homolog, cytochrome c oxidase assembly protein (yeast) |  | 2.725 |
| 235053_at | AI077461 | *cox11* | COX11 homolog, cytochrome c oxidase assembly protein (yeast) |  | 9.091 |
| 232761_at | AL117381 | *cox4i2* | cytochrome c oxidase subunit IV isoform 2 (lung) |  | -3.758 |
| 206353_at | NM_005205 | *cox6a2* | cytochrome c oxidase subunit VIa polypeptide 2 | -2.534 | -4.440 |
| 240744_at | AW184014 | *cpa5* | carboxypeptidase A5 |  | -4.473 |
| 1555250_a_at | BC036444 | *cpeb3* | cytoplasmic polyadenylation element binding protein 3 |  | 3.115 |
| 235706_at | AW663908 | *cpm* | carboxypeptidase M | 4.310 |  |
| 237987_x_at | AV657741 | *cps1* | Carbamoyl-phosphate synthetase 1, mitochondrial | -4.372 | 4.651 |
| 217564_s_at | W80357 | *cps1* | carbamoyl-phosphate synthetase 1, mitochondrial |  | 3.497 |
| 232437_at | AA732590 | *cpsf3l* | cleavage and polyadenylation specific factor 3-like | 3.378 | -2.996 |
| 244097_at | AA815055 | *cr2* | Complement component (3d/Epstein Barr virus) receptor 2 | -3.189 |  |
| 1563897_at | AK096006 | *crabp1* | Cellular retinoic acid binding protein 1 | -2.897 |  |
| 236662_at | AI139639 | *cramp1l* | Crm, cramped-like (Drosophila) |  | -3.438 |
| 235452_at | AV708945 | *ckmt1a* | Creatine kinase, mitochondrial 1B |  | 3.413 |
| 216419_at | AK026910 | *crocc* | ciliary rootlet coiled-coil, rootletin | 3.968 |  |
| 229193_at | AA005430 | *crop* | Cisplatin resistance-associated overexpressed protein |  | -3.059 |
| 215167_at | BE567032 | *crsp2* | cofactor required for Sp1 transcriptional activation, subunit 2, 150kDa |  | -2.527 |
| 207770_x_at | NM_022644 | *csh2* | chorionic somatomammotropin hormone 2 | -5.540 |  |
| 229212_at | BE220341 | *csnk2a1* | Casein kinase 2, alpha 1 polypeptide | 3.155 |  |
| 229216_s_at | AI090987 | *csnk2a1* | Casein kinase 2, alpha 1 polypeptide | 3.788 |  |
| 206595_at | NM_001323 | *cst6* | cystatin E/M | 3.891 |  |
| 233315_at | AK024947 | *ctbp2* | C-terminal binding protein 2 |  | -3.317 |
| *1552368_at* | *NM_080618* | *ctcfl/boris* | *CCCTC-binding factor (zinc finger protein)-like* |  | *-1.980* |
| 206085_s_at | NM_001902 | *cth* | cystathionase (cystathionine gamma-lyase) | 3.058 |  |
| 234362_s_at | U90273 | *ctla4* | cytotoxic T-lymphocyte-associated protein 4 |  | 2.793 |
| 1556877_at | BC040662 | *ctnna3* | Catenin (cadherin-associated protein), alpha 3 |  | 4.525 |
| 202156_s_at | N36839 | *cugbp2* | CUG triplet repeat, RNA binding protein 2 | -6.748 |  |
| 242362_at | AI797788 | *cul3* | Cullin 3 | 3.876 | 5.464 |
| 201372_s_at | NM_003590 | *cul3* | cullin 3 | 4.808 |  |
| 237376_at | BF115815 | *cul3* | Cullin 3 |  | 10.256 |
| 232466_at | AU155661 | *cul4a* | Cullin 4A |  | -2.527 |
| 210163_at | AF030514 | *cxcl11* | chemokine (C-X-C motif) ligand 11 | -3.879 | -6.306 |
| 217028_at | AJ224869 | *cxcr4* | chemokine (C-X-C motif) receptor 4 | 2.994 |  |
| 214421_x_at | AV652420 | *cyp2c9* | cytochrome P450, family 2, subfamily C, polypeptide 9 |  | -3.729 |
| 209975_at | AF182276 | *cyp2e1* | cytochrome P450, family 2, subfamily E, polypeptide 1 | 2.817 |  |
| 1431_at | J02843 | *cyp2e1* | cytochrome P450, family 2, subfamily E, polypeptide 1 |  | -5.057 |
| 244757_at | AI692525 | *cyp2r1* | Cytochrome P450, family 2, subfamily R, polypeptide 1 |  | 4.425 |
| 205999_x_at | AF182273 | *cyp3a4* | cytochrome P450, family 3, subfamily A, polypeptide 4 | 4.386 |  |
| 205998_x_at | NM_017460 | *cyp3a4* | cytochrome P450, family 3, subfamily A, polypeptide 4 | -8.840 |  |
| 214235_at | X90579 | *cyp3a5* | cytochrome P450, family 3, subfamily A, polypeptide 5 |  | 2.907 |
| 1569259_at | BI825547 | *dab1* | CDNA clone IMAGE:5295305 ; Disabled homolog 1 (Drosophila) |  | -3.001 |
| 220263_at | NM_022001 | *dams* | SMAD in the antisense orientation | -2.539 |  |
| 1555640_at | AY170471 | *daoa* | D-amino acid oxidase activator | -3.026 |  |
| 244803_at | AI335191 | *dap3* | Death associated protein 3 | -3.798 |  |
| 1560257_at | BC039388 | *dapk2* | Death-associated protein kinase 2 | -5.498 |  |
| 222859_s_at | AA150186 | *dapp1* | dual adaptor of phosphotyrosine and 3-phosphoinositides |  | -5.353 |
| 209782_s_at | U79283 | *dbp* | D site of albumin promoter (albumin D-box) binding protein |  | 3.040 |
| 231919_at | AK024946 | *dbt* | dihydrolipoamide branched chain transacylase E2 | -3.727 |  |
| 1553768_a_at | NM_173674 | *dcbld1* | discoidin, CUB and LCCL domain containing 1 |  | -3.321 |
| 242653_at | AA609059 | *dcc* | deleted in colorectal carcinoma | -16.200 |  |
| 242988_at | AA292913 | *dcst2* | DC-STAMP domain containing 2 | -2.672 |  |
| 241217_x_at | H58209 | *ddost* | dolichyl-diphosphooligosaccharide-protein glycosyltransferase |  | 4.329 |
| 1556316_s_at | AI285192 | *ddt* | D-dopachrome tautomerase |  | -3.078 |
| 208151_x_at | NM_030881 | *ddx17* | DEAD (Asp-Glu-Ala-Asp) box polypeptide 17 | -2.863 |  |
| 230180_at | AA521056 | *ddx17* | DEAD (Asp-Glu-Ala-Asp) box polypeptide 17 |  | -3.036 |
| 201211_s_at | AF061337 | *ddx3x* | DEAD (Asp-Glu-Ala-Asp) box polypeptide 3, X-linked |  | 3.145 |
| 211255_x_at | AF064605 | *dedd* | death effector domain containing |  | 5.236 |
| 211272_s_at | AF064771 | *dgka* | diacylglycerol kinase, alpha 80kDa |  | 2.632 |
| 241995_at | BF511285 | *dguok* | deoxyguanosine kinase |  | 2.564 |
| 1569672_at | BC015609 | *dhrs4* | Dehydrogenase/reductase (SDR family) member 4 | -4.234 |  |
| 1559039_at | AK096808 | *dhx36* | DEAH (Asp-Glu-Ala-His) box polypeptide 36 |  | -3.295 |
| 215467_x_at | AK001442 | *dhx9* | DEAH (Asp-Glu-Ala-His) box polypeptide 9 |  | -4.082 |
| 232736_s_at | AL117485 | *dkfzp434p211* | POM121-like protein | -5.045 |  |
| 222384_at | AA417256 | *dkfzp564c196* | DKFZP564C196 protein |  | 6.803 |
| 233613_x_at | AU156209 | *dkfzp566e144* | REX2, RNA exonuclease 2 homolog (S. cerevisiae) |  | -3.087 |
| 224199_at | AB033941 | *dkk2* | dickkopf homolog 2 (Xenopus laevis) | -3.667 |  |
| 216856_s_at | AF264787 | *dleu2* | deleted in lymphocytic leukemia, 2 |  | -5.005 |
| 217208_s_at | AL121981 | *dlg1* | discs, large homolog 1 (Drosophila) |  | -2.514 |
| 224215_s_at | AF196571 | *dll1* | delta-like 1 (Drosophila) | -6.415 |  |
| 224163_s_at | AL136657 | *dmap1* | DNA methyltransferase 1 associated protein 1 | 2.976 |  |
| 234752_x_at | S71486 | *dmd* | Dystrophin (muscular dystrophy, Duchenne and Becker types) | -2.819 |  |
| 1560416_at | AK095018 | *dnah11* | dynein, axonemal, heavy polypeptide 11 |  | 4.566 |
| 235001_at | BG390661 | *dnaja5* | DnaJ homology subfamily A member 5 | -8.797 |  |
| 223722_at | AF176013 | *dnajc12* | DnaJ (Hsp40) homolog, subfamily C, member 12 | -5.629 |  |
| 217358_at | AL137715 | *dnajc16* | DnaJ (Hsp40) homolog, subfamily C, member 16 |  | 4.274 |
| 1569539_at | BC037935 | *doc1* | Downregulated in ovarian cancer 1 | -6.919 |  |
| 215238_s_at | AW450751 | *dock9* | dedicator of cytokinesis 9 | -4.318 |  |
| 214054_at | AI828929 | *dok2* | docking protein 2, 56kDa | -3.021 |  |
| 233778_at | AW626574 | *dpcd* | Deleted in a mouse model of primary ciliary dyskinesia |  | 6.098 |
| 228598_at | AL538781 | *dpp10* | dipeptidylpeptidase 10 |  | -2.505 |
| 1554536_at | BC008379 | *dpyd* | dihydropyrimidine dehydrogenase | 6.135 | -5.141 |
| 223625_at | AB030241 | *drctnnb1a* | down-regulated by Ctnnb1, a |  | 2.653 |
| 1553191_at | NM_020388 | *dst* | dystonin | -3.816 |  |
| 215810_x_at | AL049215 | *dst* | Dystonin | -2.853 |  |
| 244146_at | AW977964 | *dtwd1* | DTW domain containing 1 |  | -3.798 |
| 1552708_a_at | AB038770 | *dusp19* | dual specificity phosphatase 19 |  | 2.732 |
| 233838_at | AK021982 | *dym* | Dymeclin | -6.422 |  |
| 235273_at | AI674107 | *dyx1c1* | dyslexia susceptibility 1 candidate 1 |  | 14.641 |
| 240292_x_at | N50412 | *eb-1* | E2a-Pbx1-associated protein | -4.997 |  |
| 240292_x_at | N50412 | *eb-1/anks1b* | E2a-Pbx1-associated protein |  | 4.386 |
| 217497_at | AW613387 | *ecgf1* | endothelial cell growth factor 1 (platelet-derived) | -6.310 |  |
| 243303_at | AA811657 | *echdc1* | Enoyl Coenzyme A hydratase domain containing 1 | -2.582 |  |
| 220816_at | NM_012152 | *edg7* | endothelial differentiation, lysophosphatidic acid G-protein-coupled receptor, 7 |  | 4.367 |
| 230464_at | AI814092 | *edg8* | endothelial differentiation, sphingolipid G-protein-coupled receptor, 8 | 3.650 |  |
| 233875_at | AF143888 | *edil3* | EGF-like repeats and discoidin I-like domains 3 | -9.440 |  |
| 1563840_at | BC040569 | *eftud1* | elongation factor Tu GTP binding domain containing 1 | -5.515 |  |
| 242470_at | AI093963 | *eid-3* | EID-2-like inhibitor of differentiation-3 |  | 4.167 |
| 237145_at | AI953362 | *eif2ak4* | eukaryotic translation initiation factor 2 alpha kinase 4 |  | 4.950 |
| 1566892_at | AL832401 | *eif4e3* | Eukaryotic translation initiation factor 4E member 3 | 2.890 |  |
| 1554309_at | BC030578 | *eif4g3* | eukaryotic translation initiation factor 4 gamma, 3 |  | 5.181 |
| 243149_at | AI467945 | *eif4g3* | Eukaryotic translation initiation factor 4 gamma, 3 |  | -8.006 |
| 206446_s_at | NM_001971 | *ela1* | elastase 1 | -3.160 |  |
| 240437_at | AI571356 | *ela2b* | Elastase 2B | -3.188 |  |
| 205994_at | NM_001973 | *elk4* | ELK4, ETS-domain protein (SRF accessory protein 1) | -4.864 |  |
| 1567222_x_at | D17207 | *elovl5* | ELOVL family member 5, elongation of long chain fatty acids (FEN1/Elo2, SUR4/Elo3-like, yeast) |  | 4.425 |
| 239849_at | AI417595 | *emcn* | Endomucin |  | 2.639 |
| 228673_s_at | AI475647 | *eml4* | Echinoderm microtubule associated protein like 4 | -5.652 |  |
| 227803_at | AA609053 | *enpp5* | ectonucleotide pyrophosphatase/phosphodiesterase 5 (putative function) |  | -6.997 |
| *209473_at* | *AV717590* | *entpd1* | *ectonucleoside triphosphate diphosphohydrolase 1* | *-1.800* |  |
| 223253_at | BC000686 | *epdr1* | ependymin related protein 1 (zebrafish) |  | 3.115 |
| 206114_at | NM_004438 | *epha4* | EPH receptor A4 |  | -2.572 |
| 231239_at | AI002236 | *epha5* | EPH receptor A5 |  | 3.509 |
| 238533_at | AA651750 | *epha7* | EPH receptor A7 | -12.060 |  |
| 234278_at | AL110230 | *eps15* | epidermal growth factor receptor pathway substrate 15 | 5.128 |  |
| 219672_at | NM_016633 | *eraf* | erythroid associated factor | -2.754 |  |
| 215638_at | U88358 | *erbb3* | v-erb-b2 erythroblastic leukemia viral oncogene homolog 3 (avian) |  | 3.559 |
| 211626_x_at | M21535 | *erg* | v-ets erythroblastosis virus E26 oncogene like (avian) ; v-ets erythroblastosis virus E26 oncogene like (avian) |  | 3.891 |
| 233527_at | AK000493 | *esam* | Endothelial cell adhesion molecule | -3.584 |  |
| 235216_at | BG532121 | *esco1* | establishment of cohesion 1 homolog 1 (S. cerevisiae) |  | 6.061 |
| 217190_x_at | S67777 | *esr1* | estrogen receptor 1 | -2.661 |  |
| 242059_at | AW976631 | *etnk1* | Ethanolamine kinase 1 | 5.464 | 5.714 |
| 224453_s_at | BC006111 | *etnk1* | ethanolamine kinase 1 ; ethanolamine kinase 1 |  | -6.695 |
| 240477_at | R44780 | *etv1* | Ets variant gene 1 | 3.086 |  |
| 240498_at | AA704891 | *etv6* | Ets variant gene 6 (TEL oncogene) | 8.130 |  |
| 203780_at | AF275945 | *eva1* | epithelial V-like antigen 1 |  | -3.357 |
| 237157_at | AW117547 | *eve1/sh3d19* | SH3 domain protein D19 | 5.405 |  |
| 238370_x_at | AI252081 | *evi1* | Ecotropic viral integration site 1 | 6.494 | 3.610 |
| 226411_at | N32544 | *evi5l* | ecotropic viral integration site 5-like |  | -3.782 |
| 244375_at | AW873606 | *evl* | Enah/Vasp-like | -4.457 |  |
| 240698_s_at | BF591637 | *exosc2* | exosome component 2 |  | 2.770 |
| 234634_at | AK025101 | *ext1* | Exostoses (multiple) 1 | -2.768 |  |
| 242126_at | T53962 | *ext1* | Exostoses (multiple) 1 |  | 2.882 |
| 1561088_at | BC030588 | *eya4* | eyes absent homolog 4 (Drosophila) |  | -3.986 |
| 1569592_a_at | BC029374 | *f11* | Coagulation factor XI (plasma thromboplastin antecedent) | -2.784 |  |
| *205774_at* | *NM_000505* | *f12* | *coagulation factor XII (Hageman factor)* | *-2.160* |  |
| 207218_at | NM_000133 | *f9* | coagulation factor IX (plasma thromboplastic component, Christmas disease, hemophilia B) | -4.745 |  |
| 239749_at | AW205090 | *faf1* | Fas (TNFRSF6) associated factor 1 | -6.502 |  |
| 234945_at | AL138828 | *fam54a* | family with sequence similarity 54, member A |  | -3.835 |
| 225667_s_at | AI601101 | *fam84a* | family with sequence similarity 84, member A ; hypothetical LOC400944 |  | -5.537 |
| 243597_at | BE550133 | *fancb* | Fanconi anemia, complementation group B | 4.464 |  |
| 243588_at | N74058 | *farp1* | FERM, RhoGEF (ARHGEF) and pleckstrin domain protein 1 (chondrocyte-derived) |  | -3.706 |
| 237050_at | AW207725 | *farp2* | FERM, RhoGEF and pleckstrin domain protein 2 | -2.557 |  |
| 234758_at | AK026789 | *fars2* | Phenylalanine-tRNA synthetase 2 (mitochondrial) | 3.876 |  |
| 237522_at | AA058563 | *fas* | Fas (TNF receptor superfamily, member 6) | -5.465 |  |
| 210865_at | D38122 | *faslg* | Fas ligand (TNF superfamily, member 6) |  | -3.280 |
| 209696_at | D26054 | *fbp1* | fructose-1,6-bisphosphatase 1 |  | -3.084 |
| 1553682_at | NM_152441 | *fbxl14* | F-box and leucine-rich repeat protein 14 | -2.904 |  |
| 233087_at | AL133602 | *fbxl17* | F-box and leucine-rich repeat protein 17 |  | 3.759 |
| 243538_at | AA738314 | *fbxo42* | F-box protein 42 | -4.141 |  |
| 223517_at | AV722616 | *fbxo44* | F-box protein 44 | -3.388 |  |
| 222861_x_at | NM_012168 | *fbxo44* | F-box protein 44 |  | -3.398 |
| 205310_at | NM_012066 | *fbxo46* | F-box protein 46 |  | -2.701 |
| 231883_at | BF306374 | *fbxw8* | F-box and WD-40 domain protein 8 | 8.333 |  |
| 206759_at | NM_002002 | *fcer2* | Fc fragment of IgE, low affinity II, receptor for (CD23A) |  | -2.536 |
| 203561_at | NM_021642 | *fcgr2a* | Fc fragment of IgG, low affinity IIa, receptor (CD32) | -11.350 |  |
| 1555153_s_at | BC014311 | *fcho2* | FCH domain only 2 | -5.266 |  |
| 235401_s_at | AL560266 | *fcrlm1* | Fc receptor-like and mucin-like 1 | -27.730 | -3.483 |
| 214284_s_at | AA022949 | *fgf18* | Fibroblast growth factor 18 | -4.414 |  |
| 243619_at | R91766 | *fgfr1op2* | FGFR1 oncogene partner 2 | 2.558 |  |
| 236808_at | AA947475 | *fgfr1op2* | FGFR1 oncogene partner 2 |  | -2.603 |
| 220170_at | NM_020482 | *fhl5* | four and a half LIM domains 5 | 4.525 |  |
| 1555191_a_at | BC021723 | *fhl5* | four and a half LIM domains 5 |  | 6.173 |
| 208588_at | NM_021631 | *fksg2* | apoptosis inhibitor |  | -2.534 |
| 1564158_a_at | AK095020 | *flj10305* | Vac14 homolog (S. cerevisiae) | -5.400 |  |
| 231011_at | AI339785 | *flj10378* | La ribonucleoprotein domain family, member 2 | -2.696 |  |
| 235402_at | AI866146 | *flj32771* | IIIG9 protein |  | 3.300 |
| 243111_at | AF150317 | *flj34077* | Weakly similar to zinc finger protein 195 |  | -7.527 |
| 224526_at | AF315716 | *flj41238* | Family with sequence similarity 79, member B |  | -3.297 |
| 234106_s_at | BC001973 | *flywch1* | FLYWCH-type zinc finger 1 | -2.896 |  |
| 1563559_at | AL833157 | *fmn1* | formin 1 |  | 4.902 |
| 230946_at | BG149866 | *fmn2* | Formin 2 |  | -5.011 |
| 240422_at | AI935710 | *fmo5* | flavin containing monooxygenase 5 | 6.623 | -5.050 |
| 1569688_at | BC020872 | *fmo5* | flavin containing monooxygenase 5 |  | -6.691 |
| 230389_at | BE046511 | *fnbp1* | Formin binding protein 1 | -5.762 |  |
| *231343_at* | *AW300131* | *fndc3a* | *Fibronectin type III domain containing 3A* |  | *-1.920* |
| 204437_s_at | NM_016725 | *folr1* | folate receptor 1 (adult) | -6.173 |  |
| 202724_s_at | NM_002015 | *foxo1a* | forkhead box O1A (rhabdomyosarcoma) | -4.140 |  |
| 1569669_at | BM550294 | *foxr2* | forkhead box R2 |  | -2.535 |
| 1565717_s_at | BE930017 | *fus* | fusion (involved in t(12;16) in malignant liposarcoma) |  | 10.417 |
| 214088_s_at | AW080549 | *fut3* | fucosyltransferase 3 (galactoside 3(4)-L-fucosyltransferase, Lewis blood group included) | -2.695 |  |
| 211795_s_at | AF198052 | *fyb* | FYN binding protein (FYB-120/130) |  | -4.186 |
| 216033_s_at | S74774 | *fyn* | FYN oncogene related to SRC, FGR, YES |  | 3.906 |
| 1555612_s_at | BC020700 | *g6pc* | glucose-6-phosphatase, catalytic (glycogen storage disease type I, von Gierke disease) | -4.055 |  |
| 207014_at | NM_000807 | *gabra2* | gamma-aminobutyric acid (GABA) A receptor, alpha 2 | -18.940 |  |
| 234204_at | AK025297 | *gabrb1* | Gamma-aminobutyric acid (GABA) A receptor, beta 1 | -3.025 |  |
| 208155_x_at | NM_001476 | *gage* | G antigen 4 ; G antigen 5 ; G antigen 6 ; G antigen 7B | -5.494 |  |
| 207466_at | NM_015973 | *gal* | galanin |  | -4.917 |
| 232110_at | AK022198 | *galnt5* | UDP-N-acetyl-alpha-D-galactosamine:polypeptide N-acetylgalactosaminyltransferase 5 (GalNAc-T5) | -3.156 |  |
| 216757_at | AK024995 | *galnt7* | UDP-N-acetyl-alpha-D-galactosamine:polypeptide N-acetylgalactosaminyltransferase 7 (GalNAc-T7) | -7.314 |  |
| 230417_at | AI097463 | *galntl1* | UDP-N-acetyl-alpha-D-galactosamine:polypeptide N-acetylgalactosaminyltransferase-like 1 |  | 5.128 |
| 204471_at | NM_002045 | *gap43* | growth associated protein 43 | -3.631 |  |
| 244822_at | AA811244 | *gart* | Phosphoribosylglycinamide formyltransferase, phosphoribosylglycinamide synthetase, phosphoribosylaminoimidazole synthetase |  | 4.000 |
| 234488_s_at | AF198534 | *gcl ; gmcl1l* | germ cell-less homolog 1 (Drosophila) ; germ cell-less homolog 1 (Drosophila)-like |  | -2.645 |
| 215595_x_at | AK023918 | *gcnt2* | Glucosaminyl (N-acetyl) transferase 2, I-branching enzyme | -4.083 |  |
| 1554591_at | BC020934 | *gdep* | Gene differentially expressed in prostate | -3.218 |  |
| 232296_s_at | AK000780 | *gfm1* | G elongation factor, mitochondrial 1 | 6.757 |  |
| 1554670_at | BC029388 | *gga1* | golgi associated, gamma adaptin ear containing, ARF binding protein 1 | -3.116 |  |
| 215353_at | AL080068 | *gimap5* | GTPase, IMAP family member 5 |  | -2.838 |
| 215430_at | AA757089 | *gk2* | glycerol kinase 2 |  | 6.897 |
| 225714_s_at | AC006042 | *glcci1* | glucocorticoid induced transcript 1 |  | -2.840 |
| 230360_at | AW006648 | *gldn* | gliomedin |  | 5.988 |
| 208057_s_at | D14827 | *gli2* | GLI-Kruppel family member GLI2 |  | 2.725 |
| 237388_at | BF224204 | *glmn* | Glomulin, FKBP associated protein | -8.284 |  |
| 229332_at | AI653050 | *gloxd1* | glyoxalase domain containing 1 | -4.801 |  |
| 216021_s_at | AW298713 | *glra3* | glycine receptor, alpha 3 | -3.583 |  |
| 203157_s_at | AB020645 | *gls* | glutaminase | 4.065 |  |
| 244802_at | AA909218 | *glud1* | glutamate dehydrogenase 1 | -4.034 | 6.329 |
| 216590_at | AC004862 | *gnat3* | guanine nucleotide binding protein, alpha transducing 3 | -5.645 |  |
| 1555766_a_at | AF493870 | *gng2* | guanine nucleotide binding protein (G protein), gamma 2 | 3.922 |  |
| 1555766_a_at | AF493870 | *gng2* | guanine nucleotide binding protein (G protein), gamma 2 |  | -2.794 |
| 205184_at | NM_004485 | *gng4* | guanine nucleotide binding protein (G protein), gamma 4 |  | -4.508 |
| 234070_at | AK001151 | *gng7* | Guanine nucleotide binding protein (G protein), gamma 7 |  | -4.219 |
| 235540_at | AW296153 | *gnrh1* | gonadotropin-releasing hormone 1 (luteinizing-releasing hormone) | -3.145 |  |
| 216341_s_at | Z81148 | *gnrhr* | gonadotropin-releasing hormone receptor |  | -3.739 |
| 210424_s_at | AF163441 | *golga8* | golgi autoantigen, golgin subfamily a, 8A ; golgi autoantigen, golgin subfamily a, 8B | -4.263 |  |
| 236851_x_at | AI693378 | *golph3* | Golgi phosphoprotein 3 (coat-protein) | -4.728 |  |
| 215893_x_at | AF339787 | *gpc5* | Glypican 5 |  | -2.894 |
| 220907_at | NM_025048 | *gpr110* | G protein-coupled receptor 110 | -5.897 |  |
| 214558_at | NM_005288 | *gpr12* | G protein-coupled receptor 12 |  | -4.074 |
| 1569542_at | BC020926 | *gpr125* | G protein-coupled receptor 125 | 6.289 |  |
| 244509_at | AW449728 | *gpr155* | G protein-coupled receptor 155 |  | -3.591 |
| 220993_s_at | NM_030784 | *gpr63* | G protein-coupled receptor 63 ; G protein-coupled receptor 63 |  | 4.274 |
| 221149_at | NM_018485 | *gpr77* | G protein-coupled receptor 77 |  | -4.000 |
| 241172_at | AI939470 | *gria2* | glutamate receptor, ionotropic, AMPA 2 | -3.486 |  |
| 1560142_at | AJ301610 | *grik2* | glutamate receptor, ionotropic, kainate 2 | -3.276 |  |
| 210939_s_at | L76631 | *grm1* | glutamate receptor, metabotropic 1 |  | -3.927 |
| 1565389_s_at | S64316 | *grm5* | glutamate receptor, metabotropic 5 | -2.830 |  |
| 234975_at | BE544748 | *gspt1* | G1 to S phase transition 1 | -7.122 |  |
| 217595_at | AV701723 | *gspt1* | G1 to S phase transition 1 | -3.584 |  |
| 206062_at | L36861 | *guca1a* | guanylate cyclase activator 1A (retina) |  | -2.531 |
| 207884_at | NM_000180 | *gucy2d* | guanylate cyclase 2D, membrane (retina-specific) |  | 4.484 |
| 1562364_at | AL833700 | *gvin1* | GRPase, very large interferon inducible 1 |  | -3.399 |
| 231156_at | AW242782 | *hao2* | Hydroxyacid oxidase 2 (long chain) | -9.006 |  |
| 240602_at | AI801875 | *hbs1l* | HBS1-like (S. cerevisiae) |  | 4.695 |
| 208018_s_at | NM_002110 | *hck* | hemopoietic cell kinase |  | -7.824 |
| 207642_at | NM_001524 | *hcrt* | hypocretin (orexin) neuropeptide precursor | 2.890 |  |
| 1563091_at | BC030108 | *hdhd1a* | Haloacid dehalogenase-like hydrolase domain containing 1A |  | -3.198 |
| 1570251_at | BC016947 | *hectd1* | HECT domain containing 1 |  | -2.920 |
| 1565698_at | AI949651 | *hectd2* | HECT domain containing 2 | -2.749 |  |
| 243809_at | AI627810 | *hel308* | DNA helicase HEL308 |  | 2.915 |
| 242130_at | BE896267 | *herc1* | Hect (homologous to the E6-AP (UBE3A) carboxyl terminus) domain and RCC1 (CHC1)-like domain (RLD) 1 | -4.091 |  |
| 208054_at | NM_015601 | *herc4* | hect domain and RLD 4 |  | -2.649 |
| 237466_s_at | AW444502 | *hhip* | hedgehog interacting protein | -4.536 |  |
| 238869_at | AA913703 | *hif1a* | Hypoxia-inducible factor 1, alpha subunit (basic helix-loop-helix transcription factor) |  | 2.688 |
| 1555960_at | AK054976 | *hint1* | Histidine triad nucleotide binding protein 1 |  | -5.489 |
| 212291_at | AI393355 | *hipk1* | homeodomain interacting protein kinase 1 | 3.436 |  |
| 1552516_a_at | NM_152696 | *hipk1* | homeodomain interacting protein kinase 1 |  | 5.076 |
| 210148_at | AF305239 | *hipk3* | homeodomain interacting protein kinase 3 |  | 3.831 |
| 208515_at | NM_003521 | *hist1h2bm* | histone 1, H2bm |  | -3.388 |
| 232035_at | BE740761 | *hist1h4h* | histone 1, H4h |  | 8.000 |
| 230738_at | BE858063 | *hist2h4* | Histone 2, H4 | -2.646 |  |
| 205025_at | NM_005341 | *hkr3* | Krueppel-related zinc finger protein 3 | 3.185 |  |
| 211654_x_at | M17565 | *hla-dqb1* | major histocompatibility complex, class II, DQ beta 1 ; major histocompatibility complex, class II, DQ beta 1 | -2.582 |  |
| 210514_x_at | AF226990 | *hla-g* | HLA-G histocompatibility antigen, class I, G |  | 3.623 |
| 1567224_at | U29113 | *hmga2* | High mobility group AT-hook 2 | -4.957 |  |
| 234559_at | AL137630 | *hmgcll1* | 3-hydroxymethyl-3-methylglutaryl-Coenzyme A lyase-like 1 |  | 4.405 |
| 1557029_at | BC035392 | *hmmr* | Hyaluronan-mediated motility receptor (RHAMM) | 4.525 |  |
| 1560680_at | AL833513 | *hnrpa1* | heterogeneous neclear ribonucleoprotein A1 |  | 2.793 |
| 235603_at | N95466 | *hnrpu* | heterogeneous nuclear ribonucleoprotein U (scaffold attachment factor A) | -7.372 |  |
| 215489_x_at | AI871287 | *homer3* | homer homolog 3 (Drosophila) |  | -4.729 |
| 214457_at | NM_006735 | *hoxa2* | homeo box A2 |  | 4.831 |
| 242528_at | AI473887 | *hoxa3* | Homeo box A3 |  | 2.660 |
| 222320_at | AW970584 | *hrpt2 /cdc73* | Cell division cycle 73, Paf1/RNA polymerase II complex component, homolog (S. cerevisiae) | -3.254 |  |
| 242616_at | W80359 | *hsd17b12* | Hydroxysteroid (17-beta) dehydrogenase 12 |  | -7.486 |
| 213418_at | NM_002155 | *hspa6* | heat shock 70kDa protein 6 (HSP70B') | 4.808 |  |
| 230888_at | AW300278 | *hspc049* | HSPC049 protein | -3.701 |  |
| 1555464_at | BC046208 | *ifih1* | interferon induced with helicase C domain 1 |  | 3.021 |
| 211338_at | M54886 | *ifna2* | interferon, alpha 2 | 4.902 |  |
| 1553574_at | NM_176891 | *ifne1* | interferon epsilon 1 | -4.547 |  |
| 233532_x_at | N47376 | *ift52* | intraflagellar transport 53 homolog (chlamydomonas) | 3.378 |  |
| 242337_at | AI347128 | *igbp1* | Immunoglobulin (CD79A) binding protein 1 |  | 2.725 |
| 209541_at | AI972496 | *igf1* | insulin-like growth factor 1 (somatomedin C) | -2.919 |  |
| 208441_at | NM_015883 | *igf1r* | insulin-like growth factor 1 receptor | 2.933 |  |
| 1561310_at | BC041353 | *igkc* | Immunoglobulin kappa variable 1-5 | -2.816 | -4.787 |
| 209827_s_at | NM_004513 | *il16* | interleukin 16 (lymphocyte chemoattractant factor) | 3.953 |  |
| 212657_s_at | U65590 | *il1rn* | interleukin 1 receptor antagonist |  | -2.660 |
| 1552609_s_at | NM_172139 | *il28* | interleukin 28A (interferon, lambda 2) ; interleukin 28B (interferon, lambda 3) |  | 4.115 |
| 1563299_at | AF085885 | *impg1* | Interphotoreceptor matrix proteoglycan 1 | -5.672 | -3.047 |
| 207252_at | NM_003669 | *ine1* | inactivation escape 1 | -6.176 |  |
| 244817_at | AA947302 | *ing1* | Inhibitor of growth family, member 1 | -5.310 |  |
| 204552_at | AA355179 | *inpp4a* | Inositol polyphosphate-4-phosphatase, type I, 107kDa |  | 2.809 |
| 239878_at | BE695916 | *ipmk* | inositol polyphosphate multikinase | -4.421 |  |
| 1554739_at | BC032544 | *ipp* | intracisternal A particle-promoted polypeptide | 3.436 |  |
| 240415_at | N51093 | *irf2* | Interferon regulatory factor 2 | -4.222 |  |
| 203275_at | NM_002199 | *irf2* | interferon regulatory factor 2 | -2.926 |  |
| 205884_at | NM_000885 | *itga4* | integrin, alpha 4 (antigen CD49D, alpha 4 subunit of VLA-4 receptor) |  | 4.292 |
| 211579_at | U95204 | *itgb3* | integrin, beta 3 (platelet glycoprotein IIIa, antigen CD61) |  | -4.096 |
| 240941_at | BF222862 | *itsn2* | Intersectin 2 | -5.273 |  |
| 242413_at | AI814925 | *itsn2* | Intersectin 2 | -3.794 |  |
| 215616_s_at | AB020683 | *jmjd2b* | jumonji domain containing 2B |  | -3.504 |
| 205616_at | AW134812 | *kcna6* | Potassium voltage-gated channel, shaker-related subfamily, member 6 |  | -3.555 |
| 238428_at | BG542347 | *kcnj15* | potassium inwardly-rectifying channel, subfamily J, member 15 | 5.208 |  |
| 220727_at | NM_021161 | *kcnk10* | potassium channel, subfamily K, member 10 | -2.613 |  |
| 235108_at | BG105700 | *kcnk3* | Potassium channel, subfamily K, member 3 |  | -2.546 |
| 242976_at | AA281619 | *kcnq5* | potassium voltage-gated channel, KQT-like subfamily, member 5 |  | -24.660 |
| 238142_at | AW029203 | *kctd13* | Potassium channel tetramerisation domain containing 13 |  | 2.899 |
| 241299_at | AI651969 | *kiaa0590* | WD and tetratricopeptide repeats 2 |  | -2.825 |
| 1560755_at | BU852182 | *kiaa0974* | DnaJ (Hsp40) homolog, subfamily C, member 9 | -3.952 |  |
| 237379_at | N53456 | *kiaa1542* | CTD-binding SR-like protein rA9 |  | -3.951 |
| 233352_at | AK023753 | *kibra* | KIBRA protein |  | -5.474 |
| 231875_at | AF155117 | *kif21a* | kinesin family member 21A | -4.571 |  |
| 223933_at | AF063608 | *kif5a* | kinesin family member 5A |  | -2.795 |
| 218906_x_at | NM_022822 | *klc2* | kinesin light chain 2 |  | -2.732 |
| 210504_at | U65404 | *klf1* | Kruppel-like factor 1 (erythroid) | -2.613 |  |
| 1553137_s_at | AF028008 | *klf11* | Kruppel-like factor 11 |  | -5.467 |
| 226646_at | AI831932 | *klf2* | Kruppel-like factor 2 (lung) | -2.526 |  |
| 219657_s_at | NM_016531 | *klf3* | Kruppel-like factor 3 (basic) | 3.731 |  |
| 241520_x_at | AA701560 | *klhl15* | Kelch-like 15 (Drosophila) | -2.981 |  |
| 221986_s_at | AW006750 | *klhl24* | kelch-like 24 (Drosophila) | -11.690 |  |
| 221462_x_at | NM_017509 | *klk15* | kallikrein 15 |  | -5.127 |
| 207229_at | NM_006611 | *klra1* | killer cell lectin-like receptor subfamily A, member 1 |  | -3.948 |
| 207723_s_at | NM_002261 | *klrc3* | killer cell lectin-like receptor subfamily C, member 3 |  | -2.641 |
| 239793_at | AI082085 | *kns2* | Kinesin 2 | -2.847 |  |
| 206241_at | NM_002269 | *kpna5* | karyopherin alpha 5 (importin alpha 6) |  | -3.696 |
| 1560897_a_at | AF086314 | *krtap10-11* | keratin associated protein 10-11 | -3.802 |  |
| 1564921_at | AJ457066 | *krtap13-1* | keratin associated protein 13-1 |  | -4.508 |
| 241953_at | AL046589 | *krtap21-1* | keratin associated protein 21-1 |  | -4.934 |
| 233681_at | AJ406933 | *krtap3-3* | keratin associated protein 3-3 |  | 4.098 |
| 1564974_at | AJ457064 | *krtap8-1* | keratin associated protein 8-1 | -3.498 |  |
| 207670_at | NM_002283 | *krthb5* | keratin, hair, basic, 5 | -3.410 |  |
| 210306_at | U89358 | *l3mbtl* | l(3)mbt-like (Drosophila) | -5.025 |  |
| 207509_s_at | NM_002288 | *lair2* | leukocyte-associated Ig-like receptor 2 | -3.170 |  |
| 1563772_a_at | AK096422 | *lama3* | laminin, alpha 3 | -3.712 |  |
| 1554252_a_at | BC034500 | *lass3* | LAG1 longevity assurance homolog 3 (S. cerevisiae) | 5.319 |  |
| 206012_at | NM_003240 | *lefty2* | left-right determination factor 2 |  | -3.081 |
| 241266_at | AI962439 | *lgr5* | Leucine-rich repeat-containing G protein-coupled receptor 5 | -2.571 |  |
| 233367_at | AU144883 | *lifr* | Leukemia inhibitory factor receptor | -3.549 |  |
| 207857_at | NM_006866 | *lilra2* | leukocyte immunoglobulin-like receptor, subfamily A (with TM domain), member 2 | -4.344 |  |
| 1555643_s_at | AF499918 | *lilra5* | leukocyte immunoglobulin-like receptor, subfamily A (with TM domain), member 5 | -6.270 |  |
| 215838_at | AF212842 | *lilra5* | leukocyte immunoglobulin-like receptor, subfamily A (with TM domain), member 5 | -5.015 |  |
| 1570259_at | BC015843 | *lims1* | LIM and senescent cell antigen-like domains 1 |  | -3.030 |
| 231976_at | AL512693 | *lins1* | lines homolog 1 (Drosophila) |  | 3.846 |
| 220121_at | NM_018148 | *lins1* | lines homolog 1 (Drosophila) |  | -2.969 |
| 243596_at | AW182696 | *lkap* | Limkain b1 |  | 5.747 |
| 1554006_a_at | BC006503 | *llgl2* | lethal giant larvae homolog 2 (Drosophila) |  | -2.535 |
| 227317_at | AA045042 | *lmcd1* | LIM and cysteine-rich domains 1 | -7.413 |  |
| 237975_at | BF433161 | *lmx1b* | LIM homeobox transcription factor 1, beta | -3.456 |  |
| 230662_at | BG413584 | *loc149603* | Ring finger protein 187 |  | 3.077 |
| 238807_at | AW973964 | *loc157567* | Ankyrin repeat domain 46 |  | -3.188 |
| 220771_at | NM_016181 | *loc51152* | melanoma antigen | -3.530 |  |
| 221405_at | NM_016317 | *loc51190* | neutral sphingomyelinase | -2.991 |  |
| 219782_s_at | NM_016643 | *loc51333* | mesenchymal stem cell protein DSC43 |  | -8.896 |
| 228119_at | AI598213 | *lrch3* | leucine-rich repeats and calponin homology (CH) domain containing 3 | -6.223 |  |
| 232831_at | AA805239 | *lrfn5* | Leucine rich repeat and fibronectin type III domain containing 5 |  | 2.597 |
| 1562624_at | BC011360 | *lrp16* | LRP16 protein |  | 3.356 |
| 209468_at | AB017498 | *lrp5* | low density lipoprotein receptor-related protein 5 |  | -2.728 |
| 219949_at | NM_024512 | *lrrc2* | leucine rich repeat containing 2 | -2.881 |  |
| 206076_at | NM_006992 | *lrrc23* | Leucine rich repeat containing 23 | 3.968 |  |
| 1558483_at | AK098652 | *lrrc27* | leucine rich repeat containing 27 | 4.831 |  |
| 220219_s_at | NM_018001 | *lrrc37a* | leucine rich repeat containing 37A ; hypothetical protein FLJ10120 ; FLJ34306 protein ; c114 SLIT-like testicular protein |  | 2.688 |
| 1555831_s_at | BC004948 | *lrrc41* | leucine rich repeat containing 41 | 2.857 |  |
| 1553674_at | NM_145258 | *lrrc44* | leucine rich repeat containing 44 |  | -4.950 |
| 232704_s_at | AK025207 | *lrrfip2* | leucine rich repeat (in FLII) interacting protein 2 | -3.426 |  |
| 206408_at | NM_015564 | *lrrtm2* | leucine rich repeat transmembrane neuronal 2 | -3.007 |  |
| 241845_at | BE550501 | *lsm6* | LSM6 homolog, U6 small nuclear RNA associated (S. cerevisiae) |  | -4.717 |
| 210629_x_at | AF000425 | *lst1* | leukocyte specific transcript 1 |  | 5.747 |
| 231124_x_at | AI524095 | *ly9* | lymphocyte antigen 9 | 4.651 |  |
| 236409_at | AW207701 | *lyplal1* | Lysophospholipase-like 1 | -3.329 |  |
| 232283_at | AL122088 | *lysmd1* | LysM, putative peptidoglycan-binding, domain containing 1 |  | -6.213 |
| 222107_x_at | BE312985 | *lzts1* | leucine zipper, putative tumor suppressor 1 | -3.994 |  |
| 214603_at | U82671 | *magea2* | melanoma antigen family A, 2 ; melanoma antigen family A, 2B | -4.082 |  |
| 1556047_s_at | AI298393 | *magee1* | melanoma antigen family E, 1 | -3.603 |  |
| 219894_at | NM_019066 | *magel2* | MAGE-like 2 | -2.671 |  |
| 215372_x_at | AU146794 | *magi1* | Membrane associated guanylate kinase, WW and PDZ domain containing 1 | 5.405 |  |
| 1556170_at | BC040304 | *maml3* | mastermind-link 3 (Drosophila) |  | -13.030 |
| 203778_at | NM_005908 | *manba* | mannosidase, beta A, lysosomal | 3.012 |  |
| 1552631_a_at | NM_145319 | *map3k6* | mitogen-activated protein kinase kinase kinase 6 |  | 2.747 |
| 242106_at | AW972669 | *mapk1* | Mitogen-activated protein kinase 1 | -2.556 |  |
| 233017_x_at | AK022388 | *mapkbp1* | Mitogen activated protein kinase binding protein 1 |  | -2.877 |
| 233218_at | AK022239 | *mark4* | MAP/microtubule affinity-regulating kinase 4 |  | -2.675 |
| 239148_at | AI493046 | *marveld3* | MARVEL domain containing 3 |  | -6.459 |
| 238558_at | AI445833 | *mbnl1* | Muscleblind-like (Drosophila) |  | -2.817 |
| 233015_at | AA732240 | *mbnl1* | Muscleblind-like (Drosophila) |  | -2.553 |
| 230688_at | AI631029 | *mbnl2* | Muscleblind-like 2 (Drosophila) |  | -10.210 |
| 242703_at | AI298089 | *mccc1* | Methylcrotonoyl-Coenzyme A carboxylase 1 (alpha) |  | 2.793 |
| 233560_x_at | AA370141 | *mcm8* | MCM8 minichromosome maintenance deficient 8 (S. cerevisiae) | 3.390 |  |
| 1554730_at | BC030005 | *mctp1* | multiple C2-domains with two transmembrane regions 1 | -2.932 |  |
| 205375_at | NM_005586 | *mdfi* | MyoD family inhibitor | -2.876 |  |
| 205386_s_at | NM_002392 | *mdm2* | Mdm2, transformed 3T3 cell double minute 2, p53 binding protein (mouse) | 3.106 |  |
| 205655_at | NM_002393 | *mdm4* | Mdm4, transformed 3T3 cell double minute 4, p53 binding protein (mouse) | -3.335 |  |
| 241924_at | BE897518 | *mecp2* | methyl CpG binding protein 2 (Rett syndrome) | -4.477 | -3.023 |
| 221650_s_at | BC002694 | *med18* | mediator of RNA polymerase II transcription, subunit 18 homolog (yeast) |  | 2.551 |
| 219318_x_at | NM_016060 | *med31* | mediator of RNA polymerase II transcription, subunit 31 homolog (yeast) | -3.609 |  |
| 207078_at | NM_005466 | *med6* | mediator of RNA polymerase II transcription, subunit 6 homolog (yeast) | 5.650 |  |
| 231467_at | W72466 | *meg8* | Maternally expressed (in Callipyge) 8 |  | -3.149 |
| 232523_at | AU144892 | *megf10* | MEGF10 protein |  | -7.303 |
| 211913_s_at | L08961 | *mertk* | c-mer proto-oncogene tyrosine kinase |  | -4.692 |
| 219797_at | NM_012214 | *mgat4a* | mannosyl (alpha-1,3-)-glycoprotein beta-1,4-N-acetylglucosaminyltransferase, isoenzyme A | -4.444 |  |
| 1554716_s_at | BC031958 | *mgc42951* | MGC42951 gene | 4.219 |  |
| 235576_at | AW474542 | *mgc43690* | WD repeat domain 27 |  | -3.080 |
| 214972_at | AU144791 | *mgea5* | Meningioma expressed antigen 5 (hyaluronidase) | -2.544 |  |
| 221177_at | NM_025043 | *mia2* | melanoma inhibitory activity 2 |  | 4.444 |
| 234156_at | AK026905 | *mical2* | Microtubule associated monoxygenase, calponin and LIM domain containing 2 | -2.912 |  |
| 1559856_s_at | AF272379 | *mll* | myeloid/lymphoid or mixed-lineage leukemia (trithorax homolog, Drosophila) | -5.343 |  |
| 229775_s_at | AI480107 | *mllt4* | Myeloid/lymphoid or mixed-lineage leukemia (trithorax homolog, Drosophila); translocated to, 4 |  | 3.165 |
| 238421_at | N86386 | *mnab* | Membrane associated DNA binding protein | 5.128 |  |
| 203949_at | NM_000250 | *mpo* | myeloperoxidase | -5.807 |  |
| 1553504_at | NM_054032 | *mrgprx4* | MAS-related GPR, member X4 | -2.723 |  |
| 221997_s_at | AI560951 | *mrpl52* | Mitochondrial ribosomal protein L52 |  | 3.135 |
| 220103_s_at | NM_016067 | *mrps18c* | mitochondrial ribosomal protein S18C |  | -4.442 |
| 224873_s_at | AK024433 | *mrps25* | mitochondrial ribosomal protein S25 |  | -2.886 |
| 232725_s_at | BF338860 | *ms4a6a* | membrane-spanning 4-domains, subfamily A, member 6A | 5.128 |  |
| 209928_s_at | AF060154 | *msc* | musculin (activated B-cell factor-1) |  | -3.064 |
| 217292_at | AL117549 | *mtmr7* | myotubularin related protein 7 | -6.464 |  |
| 215793_at | AF073482 | *mtmr7* | myotubularin related protein 7 |  | -6.928 |
| 210359_at | AF116674 | *mtss1* | metastasis suppressor 1 |  | -3.240 |
| 212096_s_at | AL096842 | *mtus1* | mitochondrial tumor suppressor 1 |  | 3.759 |
| 233891_at | AF007192 | *muc3* | SIB 297 intestinal mucin (MUC3) | -3.527 |  |
| 1565666_s_at | AW864944 | *muc6* | mucin 6, gastric |  | 5.988 |
| 229160_at | AI967987 | *mum1l1* | melanoma associated antigen (mutated) 1-like 1 | -5.909 |  |
| 1555681_at | AF384996 | *musp1* | MUSP1 (MUSP1) |  | -2.752 |
| *220471_s_at* | *NM_025107* | *myct1* | *myc target 1* | *-2.110* | -5.213 |
| 217274_x_at | X52005 | *myl4* | myosin, light polypeptide 4, alkali; atrial, embryonic | -3.018 |  |
| 240006_at | AI522109 | *myo5b* | Myosin VB |  | -6.884 |
| 236022_at | AI650341 | *myohd1* | Myosin head domain containing 1 |  | -2.996 |
| 242480_at | AA868356 | *myst3* | MYST histone acetyltransferase (monocytic leukemia) 3 | -5.429 |  |
| 216017_s_at | AJ011081 | *nab2* | NGFI-A binding protein 2 (EGR1 binding protein 2) | -3.342 |  |
| 211824_x_at | AF229062 | *nac* | NACHT, leucine rich repeat and PYD (pyrin domain) containing 1 | 2.882 |  |
| 1562257_x_at | AL832400 | *nac* | NACHT, leucine rich repeat and PYD (pyrin domain) containing 1 | 4.292 |  |
| 210496_at | AF210651 | *nag18* | NAG18 protein |  | -3.322 |
| 1553534_at | NM_176821 | *nalp10* | NACHT, leucine rich repeat and PYD containing 10 |  | 3.311 |
| 1552405_at | NM_153447 | *nalp5* | NACHT, leucine rich repeat and PYD containing 5 | -2.787 |  |
| 233539_at | AK000801 | *nape-pld* | N-acyl-phosphatidylethanolamine-hydrolyzing phospholipase D | -2.907 | -2.972 |
| 242639_at | AW340004 | *narg2* | NMDA receptor regulated 2 | 4.386 | -3.964 |
| 242986_at | BF513384 | *nav1* | Neuron navigator 1 |  | 4.785 |
| 1556606_at | BU739339 | *nav2* | neuron navigator 2 | -2.502 |  |
| 205147_x_at | NM_000631 | *ncf4* | neutrophil cytosolic factor 4, 40kDa | 2.681 | 3.891 |
| 237076_at | AI634534 | *ncstn* | nicastrin | 6.897 |  |
| 1554719_at | BC031332 | *ndufa10* | NADH dehydrogenase (ubiquinone) 1 alpha subcomplex, 10, 42kDa |  | -4.180 |
| 211407_at | M33374 | *ndufb7* | NADH dehydrogenase (ubiquinone) 1 beta subcomplex, 7, 18kDa | -4.149 |  |
| 242676_at | AA401733 | *ndufv2* | NADH dehydrogenase (ubiquinone) flavoprotein 2, 24 kDa |  | 4.608 |
| 239450_at | AA846867 | *ndufv2* | NADH dehydrogenase (ubiquinone) flavoprotein 2, 24 kDa |  | -4.931 |
| 1559495_at | BC039122 | *necap1* | NECAP endocytosis associated 1 |  | -2.905 |
| 230283_at | AW298115 | *neurl2* | neuralized-like 2 (Drosophila) |  | 3.690 |
| 210271_at | AB021742 | *neurod2* | neurogenic differentiation 2 | -4.008 |  |
| 214295_at | AW129056 | *nfia* | Nuclear factor I/A | 8.130 |  |
| 230791_at | AU146924 | *nfib* | Nuclear factor I/B | -3.310 |  |
| 241797_at | AI904095 | *nfix* | Nuclear factor I/X (CCAAT-binding transcription factor) |  | 5.917 |
| 215228_at | AA166895 | *nhlh2* | nescient helix loop helix 2 | -4.202 |  |
| 223764_x_at | BC005202 | *nipsnap3b* | nipsnap homolog 3B (C. elegans) | -6.717 |  |
| 1557737_s_at | AI880383 | *nktr* | natural killer-tumor recognition sequence | -2.705 | 2.907 |
| 219726_at | NM_018977 | *nlgn3* | neuroligin 3 | -3.028 | -2.841 |
| 223484_at | AF228422 | *nmes1* | normal mucosa of esophagus specific 1 |  | -5.708 |
| 244531_at | BE501279 | *nnt* | Nicotinamide nucleotide transhydrogenase |  | -3.969 |
| 218591_s_at | NM_024894 | *nol10* | nucleolar protein 10 | -2.965 |  |
| 221566_s_at | AF043244 | *nol3* | nucleolar protein 3 (apoptosis repressor with CARD domain) |  | -4.805 |
| 206045_s_at | NM_003787 | *nol4* | nucleolar protein 4 |  | 14.327 |
| 207309_at | NM_000620 | *nos1* | nitric oxide synthase 1 (neuronal) | -4.295 |  |
| 1560974_s_at | BF447723 | *nos1* | nitric oxide synthase 1 (neuronal) | -3.283 |  |
| 204501_at | NM_002514 | *nov* | nephroblastoma overexpressed gene | -2.701 |  |
| 207437_at | NM_006491 | *nova1* | neuro-oncological ventral antigen 1 |  | 9.804 |
| 207400_at | NM_006174 | *npy5r* | neuropeptide Y receptor Y5 | 2.924 |  |
| 206645_s_at | NM_000475 | *nr0b1* | nuclear receptor subfamily 0, group B, member 1 | -2.839 |  |
| 240035_at | T26531 | *nr1i2* | Nuclear receptor subfamily 1, group I, member 2 |  | -3.702 |
| 243240_at | R37780 | *nrcam* | Neuronal cell adhesion molecule |  | -5.026 |
| 210683_at | AL161995 | *nrtn* | neurturin | -3.335 |  |
| *228547_at* | *AA776739* | *nrxn1* | *neurexin 1* | *-3.430* |  |
| 221606_s_at | BC005342 | *nsbp1* | nucleosomal binding protein 1 |  | 3.333 |
| 243612_at | AL526448 | *nsd1* | Nuclear receptor binding SET domain protein 1 | -4.917 |  |
| 1569181_x_at | BC017896 | *nsf* | N-ethylmaleimide-sensitive factor |  | -5.130 |
| 222128_at | U80764 | *nsun6* | NOL1/NOP2/Sun domain family, member 6 |  | -2.992 |
| 243100_at | AA974253 | *nt5c1b* | 5'-nucleotidase, cytosolic IB | -2.953 | 3.610 |
| 244863_at | AW779317 | *nup188* | Nucleoporin 188kDa |  | -3.150 |
| 218293_x_at | AW589982 | *nup50* | nucleoporin 50kDa |  | 2.817 |
| 241425_at | AA769986 | *nupl1* | Nucleoporin like 1 |  | -7.046 |
| 214945_at | AW514267 | *ny-ren-7* | NY-REN-7 antigen ; similar to KIAA0752 protein | 3.390 |  |
| 227379_at | AI734993 | *oact1* | O-acyltransferase (membrane bound) domain containing 1 |  | -2.926 |
| 202793_at | NM_005768 | *oact5* | O-acyltransferase (membrane bound) domain containing 5 | 3.623 |  |
| 205552_s_at | NM_002534 | *oas1* | 2',5'-oligoadenylate synthetase 1, 40/46kDa |  | 3.205 |
| 204972_at | NM_016817 | *oas2* | 2'-5'-oligoadenylate synthetase 2, 69/71kDa | 6.061 |  |
| 210797_s_at | AF063612 | *oasl* | 2'-5'-oligoadenylate synthetase-like | -2.641 |  |
| 214485_at | NM_024410 | *odf1* | outer dense fiber of sperm tails 1 | 2.538 |  |
| 1552408_at | NM_153007 | *odf4* | outer dense fiber of sperm tails 4 |  | -2.590 |
| 1564590_a_at | AF520805 | *ofcc1* | orofacial cleft 1 candidate 1 | -6.933 |  |
| 213825_at | AA757419 | *olig2* | oligodendrocyte lineage transcription factor 2 |  | -3.046 |
| 1556371_at | AK096362 | *olig3* | oligodendrocyte transcription factor 3 | -5.634 |  |
| 223884_at | AF161702 | *optc* | opticin |  | -4.244 |
| 221460_at | NM_012368 | *or2c1* | olfactory receptor, family 2, subfamily C, member 1 | -2.598 | -2.554 |
| 217302_at | AC004853 | *or2f2* | olfactory receptor, family 2, subfamily F, member 2 | -3.194 |  |
| 241881_at | N54813 | *or2w3* | olfactory receptor, family 2, subfamily W, member 3 |  | 3.145 |
| 1567246_at | X64988 | *or5h1* | olfactory receptor, family 5, subfamily H, member 1 | -8.915 |  |
| 1567287_at | X64984 | *or5k1* | olfactory receptor, family 5, subfamily K, member 1 | 5.000 |  |
| 216690_at | AC005255 | *or7c1* | olfactory receptor, family 7, subfamily C, member 1 | -4.441 |  |
| 227548_at | AI923278 | *ormdl1* | ORM1-like 1 (S. cerevisiae) |  | 2.625 |
| 223432_at | BE501253 | *osbp2* | oxysterol binding protein 2 | -4.420 |  |
| 231656_x_at | AW593467 | *osbpl10* | oxysterol binding protein-like 10 |  | -2.857 |
| 216755_at | AK024945 | *osbpl10* | oxysterol binding protein-like 10 |  | -2.600 |
| 243287_s_at | H04482 | *ostm1* | Osteopetrosis associated transmembrane protein 1 | 3.774 |  |
| 223879_s_at | AF309387 | *oxr1* | oxidation resistance 1 |  | 2.874 |
| 207455_at | NM_002563 | *p2ry1* | purinergic receptor P2Y, G-protein coupled, 1 |  | -4.864 |
| 220402_at | NM_022112 | *p53aip1* | p53-regulated apoptosis-inducing protein 1 | -7.432 |  |
| 208876_s_at | AI076186 | *pak2* | p21 (CDKN1A)-activated kinase 2 |  | -2.988 |
| 1569904_x_at | BC015161 | *palld* | Palladin |  | -2.970 |
| 240141_at | BF062399 | *papd4* | PAP associated domain containing 4 | 3.125 |  |
| 226435_at | AU145309 | *papln* | papilin, proteoglycan-like sulfated glycoprotein | -5.618 |  |
| 240295_at | AL045014 | *pappa* | Pregnancy-associated plasma protein A, pappalysin 1 |  | -5.224 |
| 213332_at | AL031290 | *pappa2* | Pappalysin 2 |  | 2.933 |
| 227216_at | AI560765 | *pard6a/rltpr* | RGD, leucine-rich repeat, tropomodulin and proline-rich containing protein |  | 5.291 |
| 233510_s_at | AL355092 | *parvg* | parvin, gamma |  | 2.915 |
| 228243_at | AI949772 | *paxip1l* | PAX interacting (with transcription-activation domain) protein 1 | -8.412 |  |
| 221212_x_at | NM_018313 | *pb1* | polybromo 1 |  | -4.228 |
| 203557_s_at | NM_000281 | *pcbd1* | 6-pyruvoyl-tetrahydropterin synthase/dimerization cofactor of hepatocyte nuclear factor 1 alpha (TCF1) | 2.857 |  |
| 223712_at | AL136721 | *pcbd2* | 6-pyruvoyl-tetrahydropterin synthase/dimerization cofactor of hepatocyte nuclear factor 1 alpha (TCF1) 2 |  | 3.165 |
| 228635_at | AI640307 | *pcdh10* | protocadherin 10 | 3.861 |  |
| 217049_x_at | AJ276803 | *pcdh11y* | protocadherin 11 Y-linked | -3.448 |  |
| 217049_x_at | AJ276803 | *pcdh11y* | protocadherin 11 Y-linked |  | 6.369 |
| 206935_at | NM_002590 | *pcdh8* | protocadherin 8 | 2.770 |  |
| 1559341_at | BC042366 | *pcdh9* | Protocadherin 9 |  | -5.332 |
| 203793_x_at | NM_007144 | *pcgf2* | polycomb group ring finger 2 | -2.828 |  |
| 235331_x_at | AI341142 | *pcgf5* | polycomb group ring finger 5 |  | 16.639 |
| 213558_at | AB011131 | *pclo* | piccolo (presynaptic cytomatrix protein) |  | -4.213 |
| 208591_s_at | NM_000922 | *pde3b* | phosphodiesterase 3B, cGMP-inhibited | -2.710 |  |
| 205463_s_at | NM_002607 | *pdgfa* | platelet-derived growth factor alpha polypeptide |  | -3.373 |
| 242171_at | AA693730 | *pdgfc* | platelet derived growth factor c | -3.430 |  |
| 222719_s_at | AB033831 | *pdgfc* | platelet derived growth factor C |  | 2.558 |
| 209493_at | AF338650 | *pdzk2* | PDZ domain containing 2 | 2.732 |  |
| 209242_at | AL042588 | *peg3* | paternally expressed 3 |  | 6.098 |
| 232304_at | AK026714 | *peli1* | Pellino homolog 1 (Drosophila) | 3.861 |  |
| 1561129_at | AF085976 | *peli2* | Pellino homolog 2 (Drosophila) | -3.309 |  |
| 1555131_a_at | BC026102 | *per3* | period homolog 3 (Drosophila) |  | -2.996 |
| 1566844_at | AF348410 | *per4* | period 4 pseudogene |  | -2.950 |
| 206351_s_at | NM_002617 | *pex10* | peroxisome biogenesis factor 10 |  | -2.968 |
| 1558163_at | BC040953 | *pex13* | Peroxisome biogenesis factor 13 |  | -8.071 |
| 244684_at | AI432340 | *pggt1b* | protein geranylgeranyltransferase type I, beta subunit |  | 3.049 |
| *1561642_at* | *AF085859* | *phactr1* | *Phosphatase and actin regulator 1* |  | *-1.820* |
| 1566030_at | AL832577 | *phactr3* | phosphatase and actin regulator 3 |  | 2.762 |
| 231967_at | AI913146 | *phf20l1* | PHD finger protein 20-like 1 |  | 4.444 |
| 239861_at | AW205964 | *phf20l1* | PHD finger protein 20-like 1 |  | -2.929 |
| 244791_at | AA835936 | *phgdhl1* | Phosphoglycerate dehydrogenase like 1 |  | -3.698 |
| 1556730_at | AK074510 | *phlda1* | Pleckstrin homology-like domain, family A, member 1 |  | -3.562 |
| 214609_at | AI469991 | *phox2a* | paired-like (aristaless) homeobox 2a | -2.984 |  |
| 215832_x_at | AV722190 | *picalm* | phosphatidylinositol binding clathrin assembly protein |  | 2.653 |
| 239102_s_at | AW293296 | *picalm* | Phosphatidylinositol binding clathrin assembly protein |  | 3.003 |
| 215969_at | AL079289 | *piga* | Phosphatidylinositol glycan, class A (paroxysmal nocturnal hemoglobinuria) | -3.064 |  |
| 1568986_x_at | AF305815 | *pigt* | Phosphatidylinositol glycan, class T | -2.843 |  |
| 234426_x_at | AF232217 | *pih2* | Pregnancy-induced hypertension syndrome-related protein (PIH2) | -17.150 |  |
| 211580_s_at | AF028785 | *pik3r3* | phosphoinositide-3-kinase, regulatory subunit 3 (p55, gamma) | -2.590 |  |
| 214225_at | BE674061 | *pin4* | protein (peptidyl-prolyl cis/trans isomerase) NIMA-interacting, 4 (parvulin) |  | -3.501 |
| 1558600_a_at | AA532745 | *pip5k2b* | Phosphatidylinositol-4-phosphate 5-kinase, type II, beta |  | -6.675 |
| 242926_at | AI798124 | *pip5k3* | Phosphatidylinositol-3-phosphate/phosphatidylinositol 5-kinase, type III | -6.141 | 3.279 |
| 221605_s_at | AF136970 | *pipox* | pipecolic acid oxidase | 5.236 |  |
| 242074_at | AA833902 | *pitpnb* | Phosphatidylinositol transfer protein, beta | -2.980 |  |
| 214868_at | AW189518 | *piwil1* | piwi-like 1 (Drosophila) | -4.666 |  |
| 226864_at | BF245954 | *pkia* | Protein kinase (cAMP-dependent, catalytic) inhibitor alpha |  | -2.805 |
| 216004_s_at | AP001748 | *pknox1* | PBX/knotted 1 homeobox 1 | 3.021 |  |
| 207717_s_at | NM_004572 | *pkp2* | plakophilin 2 | -3.417 |  |
| 236752_at | AA913146 | *pkp4* | Plakophilin 4 |  | -3.919 |
| 219584_at | NM_015900 | *pla1a* | phospholipase A1 member A | -4.318 |  |
| 215938_s_at | AK001290 | *pla2g6* | phospholipase A2, group VI (cytosolic, calcium-independent) | -3.415 |  |
| 216551_x_at | AL110247 | *plcg1* | phospholipase C, gamma 1 | -3.192 |  |
| 216218_s_at | AK023546 | *plcl2* | phospholipase C-like 2 | -6.918 |  |
| 216217_at | AK023546 | *plcl2* | phospholipase C-like 2 | -3.127 |  |
| 214745_at | AW665865 | *plcl3* | phospholipase C-like 3 | -5.205 |  |
| 222795_s_at | BE675241 | *plcxd1* | phosphatidylinositol-specific phospholipase C, X domain containing 1 | -3.588 |  |
| 228202_at | AI969945 | *pln* | Phospholamban |  | 3.086 |
| 240638_at | AI829674 | *plxnc1* | Plexin C1 |  | -8.411 |
| 217123_x_at | S64288 | *pmchl1* | pro-melanin-concentrating hormone-like 1 | -7.960 |  |
| 212705_x_at | BF570210 | *pnpla2* | patatin-like phospholipase domain containing 2 |  | -3.390 |
| 215281_x_at | AK000199 | *pogz* | pogo transposable element with ZNF domain |  | -4.726 |
| 241287_x_at | R07343 | *poln* | Polymerase (DNA directed) nu |  | -2.833 |
| 206344_at | U53784 | *pon1* | paraoxonase 1 |  | -3.689 |
| 213449_at | D31765 | *pop1* | processing of precursor 1, ribonuclease P/MRP subunit (S. cerevisiae) | 4.739 |  |
| 211341_at | L20433 | *pou4f1* | POU domain, class 4, transcription factor 1 |  | -3.196 |
| 244689_at | AA461080 | *ppara* | peroxisome proliferative activated receptor, alpha |  | 2.571 |
| 242218_at | AI201116 | *ppard* | peroxisome proliferative activated receptor, delta |  | -2.750 |
| 201490_s_at | NM_005729 | *ppif* | peptidylprolyl isomerase F (cyclophilin F) |  | 5.000 |
| 236302_at | R40892 | *ppm1e* | protein phosphatase 1E (PP2C domain containing) | -3.870 |  |
| 1555091_at | BC013798 | *ppm1f* | Protein phosphatase 1F (PP2C domain containing) |  | -2.895 |
| 244011_at | BF507342 | *ppm1k* | protein phosphatase 1K (PP2C domain containing) | -5.055 |  |
| 1566301_at | AV755778 | *ppp1r11* | Protein phosphatase 1, regulatory (inhibitor) subunit 11 |  | -4.554 |
| 225124_at | BF508705 | *ppp1r9b* | protein phosphatase 1, regulatory subunit 9B, spinophilin |  | -2.990 |
| 222351_at | AW009884 | *ppp2r1b* | protein phosphatase 2 (formerly 2A), regulatory subunit A (PR 65), beta isoform | -3.255 |  |
| 1561658_at | AF086066 | *ppp2r2b* | Protein phosphatase 2 (formerly 2A), regulatory subunit B (PR 52), beta isoform | -6.246 |  |
| 243991_at | R49315 | *ppp2r3a* | Protein phosphatase 2 (formerly 2A), regulatory subunit B'', alpha | -5.940 |  |
| 242796_x_at | AI052441 | *ppp2r3a* | Protein phosphatase 2 (formerly 2A), regulatory subunit B'', alpha | -4.899 |  |
| 1557637_at | BC038734 | *ppp3ca* | Protein phosphatase 3 (formerly 2B), catalytic subunit, alpha isoform (calcineurin A alpha) |  | -2.687 |
| 1560175_at | AK057583 | *ppp4r1l* | protein phosphatase 4, regulatory subunit 1-like | -3.297 |  |
| 240224_at | AW070207 | *prdm10* | PR domain containing 10 |  | -3.335 |
| 232424_at | AI623202 | *prdm16* | PR domain containing 16 | -3.692 |  |
| 239655_at | AA744843 | *prdm2* | PR domain containing 2, with ZNF domain |  | -5.039 |
| 203056_s_at | AI681013 | *prdm2* | PR domain containing 2, with ZNF domain |  | -3.699 |
| 242751_at | N55072 | *prdx6* | Peroxiredoxin 6 | -13.520 |  |
| 214617_at | AI445650 | *prf1* | perforin 1 (pore forming protein) ; perforin 1 (pore forming protein) |  | -4.540 |
| 230087_at | AI823645 | *prima1* | Proline rich membrane anchor 1 |  | -11.610 |
| 241669_x_at | AI251399 | *prkd2* | protein kinase D2 | 5.000 |  |
| 233365_at | AU147809 | *prkg1* | Protein kinase, cGMP-dependent, type I |  | -6.025 |
| 1559188_x_at | AL833178 | *prkxp1* | protein kinase, X-linked, pseudogene 1 | -3.940 |  |
| 223813_at | AF187844 | *prnd* | prion protein 2 (dublet) |  | -5.882 |
| 211218_at | AF118078 | *pro1848* | PRO1848 protein | -2.840 |  |
| 1560934_at | AA693612 | *prox1* | Prospero-related homeobox 1 | 2.538 |  |
| 232473_at | AU144329 | *prpf18* | PRP18 pre-mRNA processing factor 18 homolog (S. cerevisiae) | -2.791 |  |
| 219183_s_at | NM_013385 | *pscd4* | pleckstrin homology, Sec7 and coiled-coil domains 4 |  | -3.728 |
| 209606_at | L06633 | *pscdbp* | pleckstrin homology, Sec7 and coiled-coil domains, binding protein ; pleckstrin homology, Sec7 and coiled-coil domains, binding protein |  | -2.593 |
| 1555366_at | AY176665 | *psd3* | Pleckstrin and Sec7 domain containing 3 |  | -3.073 |
| 243997_x_at | AA234091 | *pspc1* | Paraspeckle component 1 |  | 5.263 |
| 244819_x_at | AI936197 | *psph* | phosphoserine phosphatase |  | -5.606 |
| 219658_at | NM_024754 | *ptcd2* | pentatricopeptide repeat domain 2 | 4.202 |  |
| 1554997_a_at | AY151286 | *ptgs2* | prostaglandin-endoperoxide synthase 2 (prostaglandin G/H synthase and cyclooxygenase) | -2.521 |  |
| 1555555_at | BC032715 | *pthb1* | parathyroid hormone-responsive B1 |  | -2.960 |
| 1556773_at | M31157 | *pthlh* | Parathyroid hormone-like hormone | 2.717 |  |
| 1559529_at | BC043202 | *ptk2* | PTK2 protein tyrosine kinase 2 |  | -4.916 |
| 1561889_at | AK094915 | *ptn* | Pleiotrophin (heparin binding growth factor 8, neurite growth-promoting factor 1) | -6.460 |  |
| 237669_at | AA053713 | *ptpdc1* | Protein tyrosine phosphatase domain containing 1 |  | -3.376 |
| 236539_at | AW665758 | *ptpn22* | protein tyrosine phosphatase, non-receptor type 22 (lymphoid) | -2.937 |  |
| 233776_at | AU147360 | *ptpra* | Protein tyrosine phosphatase, receptor type, A | -2.710 |  |
| 1560105_at | BC043182 | *ptprb* | Protein tyrosine phosphatase, receptor type, B | -7.257 | -2.697 |
| 1569830_at | BC031525 | *ptprc* | Protein tyrosine phosphatase, receptor type, C | -4.562 |  |
| 1561143_at | AF086283 | *ptprd* | Protein tyrosine phosphatase, receptor type, D |  | 4.386 |
| 233770_at | AU147115 | *ptprk* | Protein tyrosine phosphatase, receptor type, K | -2.612 |  |
| 1564552_at | BC022425 | *ptprm* | Protein tyrosine phosphatase, receptor type, M |  | 2.604 |
| 204469_at | NM_002851 | *ptprz1* | protein tyrosine phosphatase, receptor-type, Z polypeptide 1 | -13.260 |  |
| 241942_at | AA927870 | *pxdnl* | peroxidasin homolog-like (Drosophila) | -2.829 |  |
| 224210_s_at | BC001147 | *pxmp4* | peroxisomal membrane protein 4, 24kDa |  | -2.873 |
| 228540_at | BE218313 | *qki* | Quaking homolog, KH domain RNA binding (mouse) | 2.825 |  |
| 234489_at | AK021880 | *qki* | Quaking homolog, KH domain RNA binding (mouse) | 6.250 |  |
| 218948_at | AL136679 | *qrsl1* | glutaminyl-tRNA synthase (glutamine-hydrolyzing)-like 1 |  | 3.378 |
| 239164_at | BE674896 | *rab12* | RAB12, member RAS oncogene family |  | 4.545 |
| 215956_at | AK022065 | *rab5a* | RAB5A, member RAS oncogene family | -5.386 |  |
| 232565_at | AK025052 | *rab6ip2* | RAB6 interacting protein 2 |  | 2.809 |
| 239758_at | AI142126 | *rabep1* | rabaptin, RAB GTPas binding effector protein 1 | -4.041 |  |
| 1554725_at | BC041888 | *rabgap1l* | RAB GTPase activating protein 1-like | 5.051 | -4.082 |
| 213784_at | AL037167 | *rabl4* | RAB, member of RAS oncogene family-like 4 |  | -3.059 |
| 218785_s_at | NM_022777 | *rabl5* | RAB, member RAS oncogene family-like 5 |  | -2.928 |
| 209349_at | U63139 | *rad50* | RAD50 homolog (S. cerevisiae) | 4.255 |  |
| 215117_at | AW058148 | *rag2* | recombination activating gene 2 |  | 3.236 |
| 242689_at | AW468659 | *ralgps1* | Ral GEF with PH domain and SH3 binding motif 1 |  | 3.448 |
| 202639_s_at | AI689052 | *ranbp3* | RAN binding protein 3 | 2.857 |  |
| 208272_at | NM_007321 | *ranbp3* | RAN binding protein 3 | -3.318 |  |
| 243108_at | AW297762 | *ranbp9* | RAN binding protein 9 | -2.664 |  |
| 244408_at | AA927995 | *rapgef1* | Rap guanine nucleotide exchange factor (GEF) 1 | -4.356 |  |
| 239646_at | BF003148 | *rapgef6* | Rap guanin nucleotide exchange factor (GEF) 6 |  | 2.950 |
| 236748_at | AI828026 | *rasgef1c* | RasGEF domain family, member 1C | -5.198 |  |
| 214369_s_at | AI688812 | *rasgrp2* | RAS guanyl releasing protein 2 (calcium and DAG-regulated) | -3.781 |  |
| 205801_s_at | NM_015376 | *rasgrp3* | RAS guanyl releasing protein 3 (calcium and DAG-regulated) | 3.774 |  |
| 212332_at | BF110947 | *rbl2* | retinoblastoma-like 2 (p130) |  | -14.270 |
| 204178_s_at | NM_006328 | *rbm14* | RNA binding motif protein 14 | 3.401 |  |
| 229291_at | AI743545 | *rbm15* | RNA binding motif protein 15 | -4.147 |  |
| *225751_at* | *BF063156* | *rbm17* | *RNA binding motif protein 17* |  | *-2.351* |
| 1557081_at | AA580691 | *rbm25* | RNA binding motif protein 25 | -3.021 |  |
| 218379_at | NM_016090 | *rbm7* | RNA binding motif protein 7 |  | 2.710 |
| 229540_at | R45471 | *rbpsuh* | recombining binding protein suppressor of hairless (Drosophila) |  | -5.462 |
| 206036_s_at | NM_002908 | *rel* | v-rel reticuloendotheliosis viral oncogene homolog (avian) | -5.509 |  |
| 209878_s_at | M62399 | *rela* | v-rel reticuloendotheliosis viral oncogene homolog A, nuclear factor of kappa light polypeptide gene enhancer in B-cells 3, p65 (avian) | 3.509 |  |
| 220873_at | NM_018562 | *reps2* | RALBP1 associated Eps domain containing 2 |  | 2.841 |
| 213114_at | AI818736 | *rer1* | RER1 retention in endoplasmic reticulum 1 homolog (S. cerevisiae) |  | 2.710 |
| 242407_at | H71242 | *rere* | Arginine-glutamic acid dipeptide (RE) repeats | -3.575 |  |
| 1552649_a_at | NM_057178 | *rffl* | ring finger and FYVE-like domain containing 1 | -3.237 |  |
| 207936_x_at | NM_006604 | *rfpl3* | ret finger protein-like 3 | -2.791 |  |
| 207234_at | NM_002919 | *rfx3* | regulatory factor X, 3 (influences HLA class II expression) |  | 3.623 |
| 217671_at | BE466926 | *rfx3* | Regulatory factor X, 3 (influences HLA class II expression) |  | 8.475 |
| 1559706_at | AB082529 | *rgnef* | Rho-guanine nucleotide exchange factor | 3.165 |  |
| 1560348_at | AK094713 | *rgnef* | Rho-guanine nucleotide exchange factor | 4.367 |  |
| 1554500_a_at | AF493931 | *rgs7* | regulator of G-protein signalling 7 | 9.346 |  |
| 240111_at | H01893 | *rhobtb3* | Rho-related BTB domain containing 3 | -2.603 |  |
| 238905_at | BE218803 | *rhoj* | ras homolog gene family, member J | 3.571 |  |
| 241820_at | BF666241 | *rif1* | RAP1 interacting factor homolog (yeast) | 2.584 |  |
| 236759_at | AW295250 | *rims3* | Regulating synaptic membrane exocytosis 3 | -3.762 |  |
| 91816_f_at | C18318 | *rkhd1* | ring finger and KH domain containing 1 |  | 4.032 |
| 211753_s_at | BC005956 | *rln1* | relaxin 1 ; relaxin 1 | -5.439 |  |
| 201785_at | NM_002933 | *rnase1* | ribonuclease, RNase A family, 1 (pancreatic) |  | 2.591 |
| 1568977_at | BC019871 | *rnaset2* | ribonuclease T2 | -3.579 |  |
| 244517_x_at | AW070573 | *rnf146* | Ring finger protein 146 | -3.427 | -3.308 |
| 1569294_at | BC015435 | *rnf187* | ring finger protein 187 | -3.265 |  |
| 215011_at | AJ006835 | *rnu17d* | RNA, U17D small nucleolar | -3.019 |  |
| 227517_s_at | AI056992 | *rnu47* | RNA, U47 small nuclear | 2.801 |  |
| 244310_at | AW973410 | *rora* | RAR-related orphan receptor A | -2.986 |  |
| 1563379_at | AF085988 | *rora* | RAR-related orphan receptor A |  | 3.802 |
| 241890_at | BE673651 | *rora* | RAR-related orphan receptor A |  | -3.795 |
| 217559_at | AI001784 | *rpl10l* | ribosomal protein L10-like |  | -2.985 |
| 219138_at | BC000606 | *rpl14* | ribosomal protein L14 | -2.535 |  |
| 216479_at | AL356414 | *rpl21* | ribosomal protein L21 ; similar to 60S ribosomal protein L21 ; ribosomal protein L21 pseudogene | -5.129 |  |
| 215249_at | AK021571 | *rpl35a* | ribosomal protein L35a | 3.236 |  |
| 210115_at | L05096 | *rpl39l* | ribosomal protein L39-like | 5.319 |  |
| 1563792_at | AK092824 | *rplp0* | Ribosomal protein, large, P0 |  | -2.746 |
| 221523_s_at | AL138717 | *rragd* | Ras-related GTP binding D | -7.287 |  |
| 235354_s_at | BG398744 | *rsrc1* | Arginine/serine-rich coiled-coil 1 | -3.461 |  |
| 1561765_at | AJ431619 | *rtn4ip1* | Reticulon 4 interacting protein 1 | -4.477 |  |
| 238550_at | BF028405 | *rufy2* | RUN and FYVE domain containing 2 | -2.982 |  |
| 236114_at | AI798118 | *runx1* | Runt-related transcription factor 1 (acute myeloid leukemia 1; aml1 oncogene) |  | 3.717 |
| 213481_at | N92920 | *s100a13* | S100 calcium binding protein A13 | -3.851 |  |
| 1561521_at | BC041935 | *s100b* | S100 calcium binding protein, beta (neural) | 5.988 | 2.558 |
| 220793_at | NM_018666 | *sage1* | sarcoma antigen 1 | -4.860 |  |
| 225650_at | BF207100 | *samd1* | sterile alpha motif domain containing 1 |  | -3.976 |
| 213988_s_at | BE971383 | *sat* | spermidine/spermine N1-acetyltransferase | 2.500 |  |
| 210593_at | M55580 | *sat* | spermidine/spermine N1-acetyltransferase | 3.623 |  |
| 230333_at | BE326919 | *sat* | Spermidine/spermine N1-acetyltransferase |  | -2.966 |
| 203408_s_at | NM_002971 | *satb1* | special AT-rich sequence binding protein 1 (binds to nuclear matrix/scaffold-associating DNA's) | -4.210 |  |
| 215591_at | AK025127 | *satb2* | SATB family member 2 | -3.063 |  |
| 231795_at | BG289281 | *sblf* | stoned B-like factor |  | -2.737 |
| 206799_at | NM_006551 | *scgb1d2* | secretoglobin, family 1D, member 2 |  | -3.845 |
| 1566772_at | AL832567 | *schip1* | schwannomin interacting protein 1 |  | 3.086 |
| 232858_at | AK021989 | *scmh1* | Sex comb on midleg homolog 1 (Drosophila) |  | 17.331 |
| 220791_x_at | NM_014139 | *scn11a* | sodium channel, voltage-gated, type XI, alpha |  | -3.008 |
| 206381_at | NM_021007 | *scn2a2* | sodium channel, voltage-gated, type II, alpha 2 | -6.574 |  |
| 232512_at | AB037777 | *scn3a* | sodium channel, voltage-gated, type III, alpha | -3.869 |  |
| 223341_s_at | AF330205 | *scoc* | short coiled-coil protein |  | -2.902 |
| 232108_at | AL566069 | *scrn3* | secernin 3 |  | -3.152 |
| 219197_s_at | AI424243 | *scube2* | signal peptide, CUB domain, EGF-like 2 |  | -2.598 |
| 230253_at | BE674338 | *scube3* | Signal peptide, CUB domain, EGF-like 3 | 3.460 |  |
| 235594_at | AL542578 | *scye1* | Small inducible cytokine subfamily E, member 1 (endothelial monocyte-activating) |  | -7.381 |
| 243963_at | AI473707 | *sdccag8* | Serologically defined colon cancer antigen 8 |  | -2.988 |
| 232894_at | AB033012 | *sec14l2* | SEC14-like 2 (S. cerevisiae) | -3.206 | 3.077 |
| 1569201_a_at | BF847120 | *sec15l2* | SEC15-like 2 (S. cerevisiae) |  | 6.711 |
| 1563052_at | BC040832 | *sec22l1* | DEC22 vesicle traficking protein homolog B (S. cerevisiae) |  | 8.264 |
| 239056_at | AA096421 | *sec22l3* | SEC22 vesicle trafficking protein-like 3 (S. cerevisiae) | -2.729 |  |
| 243765_at | AW993582 | *sec31l1* | SEC31-like 1 (S. cerevisiae) | 3.448 |  |
| 223449_at | AF225425 | *sema6a* | sema domain, transmembrane domain (TM), and cytoplasmic domain, (semaphorin) 6A |  | 2.558 |
| 215114_at | AK000923 | *senp3* | SUMO1/sentrin/SMT3 specific peptidase 3 |  | -2.845 |
| 241093_at | T91323 | *sept6* | Septin 6 (SEPT6) |  | 3.125 |
| 1553057_at | NM_080474 | *serpinb12* | serpin peptidase inhibitor, clade B (ovalbumin), member 12 | -4.383 |  |
| 211362_s_at | AF169949 | *serpinb13* | serpin peptidase inhibitor, clade B (ovalbumin), member 13 | -3.296 |  |
| 211906_s_at | AB046400 | *serpinb4* | serpin peptidase inhibitor, clade B (ovalbumin), member 4 ; serpin peptidase inhibitor, clade B (ovalbumin), member 4 | -5.037 |  |
| 242814_at | AI986192 | *serpinb9* | serpin peptidase inhibitor, clade B (ovalbumin), member 9 |  | -2.955 |
| 236512_at | AA705429 | *sesn1* | Sestrin 1 | -3.012 |  |
| 228413_s_at | BF057567 | *sfrp1* | Secreted frizzled-related protein 1 |  | -4.704 |
| 229246_at | AI803504 | *sfrs1* | Splicing factor, arginine/serine-rich 1 (splicing factor 2, alternate splicing factor) | 4.255 |  |
| 1568783_at | BC017000 | *sfrs12* | Splicing factor, arginine/serine-rich 12 | -5.808 |  |
| 243759_at | AI041854 | *sfrs15* | Splicing factor, arginine/serine-rich 15 |  | 6.494 |
| 202773_s_at | AI023864 | *sfrs8* | splicing factor, arginine/serine-rich 8 (suppressor-of-white-apricot homolog, Drosophila) |  | -3.531 |
| 1559993_at | AK091504 | *sfxn3* | Sideroflexin 3 | -4.584 |  |
| 233903_s_at | AK022655 | *sgef* | Src homology 3 domain-containing guanine nucleotide exchange factor | -2.658 |  |
| 221268_s_at | NM_030791 | *sgpp1* | sphingosine-1-phosphate phosphatase 1 ; sphingosine-1-phosphate phosphatase 1 |  | 2.597 |
| 1553176_at | AF403479 | *sh2d1b* | SH2 domain containing 1B | 3.425 |  |
| 1567028_s_at | X99660 | *sh3glp2* | SH3-domain GRB2-like pseudogene 2 | -4.658 |  |
| 1555723_at | AB062480 | *sh3md2/sh3rf1* | SH3 multiple domains 2 |  | 4.651 |
| 217048_at | Y09846 | *shc1* | SHC (Src homology 2 domain containing) transforming protein 1 |  | 3.906 |
| 241745_at | BF671564 | *shprh* | SNF2 histone linker PHD RING helicase |  | 5.076 |
| 216328_at | AF287892 | *siglec8* | sialic acid binding Ig-like lectin 8 | -4.148 |  |
| 238006_at | AI760013 | *sin3a* | SIN3 homolog A, transcription regulator (yeast) | 4.566 |  |
| 244054_at | AI760298 | *skil* | SKI-like | -6.157 |  |
| 204230_s_at | NM_020309 | *slc17a7* | solute carrier family 17 (sodium-dependent inorganic phosphate cotransporter), member 7 | -3.721 |  |
| 243713_at | AI734054 | *slc1a1* | Solute carrier family 1 (neuronal/epithelial high affinity glutamate transporter, system Xag), member 1 |  | -3.436 |
| 1554692_at | BC013112 | *slc23a2* | solute carrier family 23 (nucleobase transporters), member 2 | -3.082 |  |
| 223698_at | AL136803 | *slc25a36* | solute carrier family 25, member 36 |  | -4.070 |
| 222217_s_at | BC003654 | *slc27a3* | solute carrier family 27 (fatty acid transporter), member 3 |  | -2.854 |
| 239596_at | AA521381 | *slc30a7* | solute carrier family 30 (zinc transporter), member 7 |  | -4.428 |
| 220123_at | NM_025181 | *slc35f5* | solute carrier family 35, member F5 |  | -6.509 |
| 1553126_a_at | NM_152725 | *slc39a12* | solute carrier family 39 (zinc transporter), member 12 | -5.545 |  |
| 205799_s_at | M95548 | *slc3a1* | solute carrier family 3 (cystine, dibasic and neutral amino acid transporters, activator of cystine, dibasic and neutral amino acid transport), member 1 | | -2.502 |
| 210738_s_at | AF011390 | *slc4a4* | solute carrier family 4, sodium bicarbonate cotransporter, member 4 | -4.704 |  |
| 206628_at | NM_000343 | *slc5a1* | solute carrier family 5 (sodium/glucose cotransporter), member 1 | -2.870 |  |
| 1565880_at | AF085911 | *slc5a11* | Solute carrier family 5 (sodium/glucose cotransporter), member 11 | -3.733 |  |
| 222967_at | AB043997 | *slc5a7* | solute carrier family 5 (choline transporter), member 7 | -4.101 |  |
| 222967_at | AB043997 | *slc5a7* | solute carrier family 5 (choline transporter), member 7 |  | 4.132 |
| 237058_x_at | AI802118 | *slc6a13* | solute carrier family 6 (neurotransmitter transporter, GABA), member 13 |  | -2.765 |
| 1569916_at | BC039443 | *slc6a15* | Solute carrier family 6, member 15 | -9.124 | 8.475 |
| 1569916_at | BC039443 | *slc6a15* | Solute carrier family 6, member 15 |  |  |
| 1554988_at | BC042592 | *slc9a11* | solute carrier family 9, member 11 |  | -3.558 |
| 208039_at | NM_003048 | *slc9a2* | Solute carrier family 9 (Sodium/hydrogen exchanger), member 2 | -3.198 |  |
| 216216_at | AL122074 | *slit3* | slit homolog 3 (Drosophila) |  | -3.508 |
| 1562095_at | BC029462 | *slmap* | Sarcolemma associated protein | -3.946 |  |
| 206320_s_at | NM_005905 | *smad9* | SMAD, mothers against DPP homolog 9 (Drosophila) | -4.428 |  |
| 243655_x_at | AA938663 | *smarca4* | SWI/SNF related, matrix associated, actin dependent regulator of chromatin, subfamily a, member 4 |  | 6.061 |
| 215623_x_at | AK002200 | *smc4l1* | SMC4 structural maintenance of chromosomes 4-like 1 (yeast) |  | -3.881 |
| 229869_at | AW170044 | *smcx* | smcy homolog, X-linked (mouse) |  | -2.783 |
| 244766_at | BG180003 | *smg1* | PI-3-kinase-related kinase SMG-1 |  | -4.437 |
| 236367_at | N33174 | *smg7* | Smg-7 homolog, nonsens mediated mRNA decay factor (C. elegans) |  | -3.398 |
| 216230_x_at | M59917 | *smpd1* | sphingomyelin phosphodiesterase 1, acid lysosomal (acid sphingomyelinase) |  | -5.729 |
| 243855_at | AA250831 | *snrpn* | Small nuclear ribonucleoprotein polypeptide N | -3.638 |  |
| 241608_at | AW296081 | *sntb2* | Syntrophin, beta 2 (dystrophin-associated protein A1, 59kDa, basic component 2) |  | -2.564 |
| 234919_s_at | AJ003030 | *sntg1* | syntrophin, gamma 1 | -2.574 |  |
| 234823_at | AL137277 | *sntg2* | Syntrophin, gamma 2 | 7.246 |  |
| 230066_at | AW134876 | *snx25* | sorting nexin 25 | 2.667 |  |
| 232425_at | AK026814 | *snx25* | sorting nexin 25 |  | -2.559 |
| 1552792_at | NM_080867 | *socs4* | suppressor of cytokine signaling 4 |  | -5.847 |
| 230295_at | BF433759 | *solh* | small optic lobes homolog (Drosophila) |  | -4.233 |
| 237472_at | BE504430 | *sox1* | SRY (sex determining region Y)-box 1 | -6.235 |  |
| 1569111_at | BC040649 | *sox13* | SRY (sex determining region Y)-box 13 |  | 2.778 |
| 214633_at | AI824954 | *sox3* | SRY (sex determining region Y)-box 3 |  | -8.625 |
| 228698_at | AI808807 | *sox7* | SRY (sex determining region Y)-box 7 | 4.049 |  |
| 1553579_a_at | NM_058206 | *spag11* | sperm associated antigen 11 | -7.417 |  |
| 1560174_at | AK095036 | *spag16* | sperm associated antigen 16 |  | -3.010 |
| 221057_at | NM_022354 | *spata1* | spermatogenesis associated 1 |  | -3.733 |
| 233297_s_at | AL139377 | *spg20* | spastic paraplegia 20, spartin (Troyer syndrome) | -4.166 |  |
| 206056_x_at | X52075 | *spn* | sialophorin (gpL115, leukosialin, CD43) | -2.541 |  |
| 1556839_s_at | AA515490 | *sptbn5* | Spectrin, beta, non-erythrocytic 5 |  | -7.343 |
| 218800_at | NM_024592 | *srd5a2l* | steroid 5 alpha-reductase 2-like |  | -3.459 |
| 1554473_at | BC029919 | *srgap1* | SLIT-ROBO Rho GTPase activating protein 1 | -7.516 |  |
| 242755_at | BE672140 | *srpk2* | SFRS protein kinase 2 |  | 3.636 |
| 216684_s_at | AF257501 | *ss18* | synovial sarcoma translocation, chromosome 18 |  | 4.425 |
| 237164_at | BE463757 | *ssh1* | Slingshot homolog 1 (Drosophila) | -3.946 |  |
| 1560306_at | AF086010 | *ssh2* | Slingshot homolog 2 (Drosophila) | -5.583 |  |
| 208482_at | NM_001049 | *sstr1* | somatostatin receptor 1 |  | 4.405 |
| 1554460_at | BC027866 | *st8sia4* | ST8 alpha-N-acetyl-neuraminide alpha-2,8-sialyltransferase 4 |  | -19.540 |
| 1555320_a_at | AB052957 | *stab1* | stabilin 1 |  | 5.495 |
| 243735_at | N58363 | *statip1* | Signal transducer and activator of transcription 3 interacting protein 1 |  | 3.676 |
| 220187_at | NM_024636 | *steap4* | STEAP family member 4 | -3.461 | 4.950 |
| 242161_at | AI458049 | *stk17a* | Serine/threonine kinase 17a (apoptosis-inducing) | -5.062 |  |
| 233779_x_at | AK022046 | *stk3* | Serine/threonine kinase 3 (STE20 homolog, yeast) |  | 2.591 |
| 205411_at | NM_006282 | *stk4* | serine/threonine kinase 4 | -4.085 |  |
| 203001_s_at | NM_007029 | *stmn2* | stathmin-like 2 | -2.769 |  |
| 1560639_at | BC013933 | *stmn3* | Stathmin-like 3 | -3.860 |  |
| 1553794_at | NM_145286 | *stoml3* | stomatin (EPB72)-like 3 | -5.494 |  |
| 229378_at | N53051 | *stox1* | storkhead box 1 | -4.310 |  |
| 229513_at | AK025613 | *strbp* | Spermatid perinuclear RNA binding protein | -2.818 |  |
| 215505_s_at | AF243424 | *strn3* | striatin, calmodulin binding protein 3 |  | -3.921 |
| 1565566_a_at | H21394 | *stx7* | Syntaxin 7 | -7.812 |  |
| 1560486_at | BC028028 | *stxbp3* | syntaxin binding protein 3 |  | -5.215 |
| 215518_at | AB023223 | *stxbp5l* | syntaxin binding protein 5-like |  | -4.373 |
| 215048_at | AW663885 | *suhw2* | suppressor of hairy wing homolog 2 (Drosophila) | -2.525 |  |
| 219262_at | NM_024670 | *suv39h2* | suppressor of variegation 3-9 homolog 2 (Drosophila) |  | 5.464 |
| 222566_at | AA056099 | *suv420h1* | suppressor of variegation 4-20 homolog 1 (Drosophila) | -3.382 |  |
| 242530_at | H22448 | *sv2c* | Synaptic vesicle glycoprotein 2C | 3.831 |  |
| 229818_at | AL359592 | *svop* | SV2 related protein | -2.891 |  |
| 1560101_at | AL834286 | *syde2* | synapse defective 1, Rho GTPase, homolog 2 (C. elegans) |  | -5.021 |
| 226068_at | BF593625 | *syk* | Spleen tyrosine kinase |  | -4.741 |
| 32402_s_at | Y10931 | *sympk* | symplekin |  | -2.648 |
| 242774_at | AI684761 | *syne2* | spectrin repeat containing, nuclear envelope 2 |  | -2.636 |
| 227662_at | AA541622 | *synpo2* | synaptopodin 2 | 4.566 |  |
| 232119_at | BF984227 | *synpo2* | synaptopodin 2 |  | -4.579 |
| 240267_at | AI686283 | *syt6* | Synaptotagmin VI |  | 11.696 |
| 1552927_at | NM_152787 | *tab3* | TAK1-binding protein 3 | -2.623 |  |
| 203937_s_at | AW015313 | *taf1c* | TATA box binding protein (TBP)-associated factor, RNA polymerase I, C, 110kDa | 3.077 |  |
| 213211_s_at | AI005317 | *taf6l* | TAF6-like RNA polymerase II, p300/CBP-associated factor (PCAF)-associated factor, 65kDa | 3.584 |  |
| 242388_x_at | AW576600 | *tagap* | T-cell activation GTPase activating protein | -4.329 |  |
| 234108_at | AF264628 | *tas2r45* | taste receptor, type 2, member 45 | 3.436 |  |
| 233528_s_at | AC004997 | *tbc1d10a* | TBC1 domain family, member 10A |  | 3.077 |
| 230438_at | AI039005 | *tbx15* | T-box 15 |  | -9.198 |
| 1566501_at | AK000794 | *tbxas1* | Thromboxane A synthase 1 (platelet, cytochrome P450, family 5, subfamily A) |  | -2.567 |
| 1570627_at | BC019949 | *tceb3* | transcription elongation factor B (SIII), polypeptide 3 (110kDa, elongin A) | 5.208 |  |
| 235925_at | AW057520 | *tcf12* | Transcription factor 12 (HTF4, helix-loop-helix transcription factors 4) |  | 3.448 |
| 210875_s_at | U12170 | *tcf8* | transcription factor 8 (represses interleukin 2 expression) | -3.781 |  |
| 239952_at | AI743662 | *tcf8* | transcription factor 8 (represses IL-2 expression) |  | 2.747 |
| 240627_x_at | R38676 | *tcra* | Transcribed locus | -31.180 |  |
| 223530_at | AF227192 | *tdrkh* | tudor and KH domain containing |  | -3.434 |
| 225840_at | AA779795 | *tef* | thyrotrophic embryonic factor |  | -3.460 |
| 231448_at | AW341495 | *tenr* | testis nuclear RNA-binding protein | -3.098 |  |
| 205688_at | NM_003223 | *tfap4* | transcription factor AP-4 (activating enhancer binding protein 4) |  | 2.695 |
| 236995_x_at | AI830469 | *tfec* | transcription factor EC | -7.558 |  |
| 214378_at | BF109662 | *tfpi* | tissue factor pathway inhibitor (lipoprotein-associated coagulation inhibitor) |  | 3.096 |
| 210215_at | AF067864 | *tfr2* | transferrin receptor 2 | -4.079 |  |
| 244492_at | BF357738 | *tg* | Thyroglobulin |  | 4.184 |
| 244858_at | BF507848 | *tgif* | TGFB-induced factor (TALE family homeobox) | -3.567 |  |
| 223588_at | AL136607 | *thap2* | THAP domain containing, apoptosis associated protein 2 | 3.460 |  |
| 244190_at | AI878997 | *thap5* | THAP domain containing 5 | -3.587 |  |
| 201107_s_at | AI812030 | *thbs1* | thrombospondin 1 |  | 2.591 |
| 1316_at | X55005 | *thra* | thyroid hormone receptor, alpha (erythroblastic leukemia viral (v-erb-a) oncogene homolog, avian) |  | -3.102 |
| 236978_at | N51961 | *thrap1* | Thyroid hormone receptor associated protein 1 | -4.201 |  |
| 229657_at | BF431989 | *thrb* | thyroid hormone receptor, beta (erythroblastic leukemia viral (v-erb-a) oncogene homolog 2, avian) |  | -2.595 |
| 1552523_a_at | NM_145720 | *tigd4* | tigger transposable element derived 4 |  | -3.213 |
| 224038_at | AF116628 | *timm23* | translocase of inner mitochondrial membrane 23 homolog (yeast) |  | -2.881 |
| 244476_at | R39769 | *tjp1* | Tight junction protein 1 (zona occludens 1) | -2.659 |  |
| 204277_s_at | BE895437 | *tk2* | thymidine kinase 2, mitochondrial | 2.874 |  |
| 216997_x_at | AL358975 | *tle4* | transducin-like enhancer of split 4 (E(sp1) homolog, Drosophila) | 2.577 |  |
| 216997_x_at | AL358975 | *tle4* | transducin-like enhancer of split 4 (E(sp1) homolog, Drosophila) |  | 2.941 |
| 223750_s_at | AW665250 | *tlr10* | toll-like receptor 10 | 3.534 |  |
| 220146_at | NM_016562 | *tlr7* | toll-like receptor 7 | 4.329 | -6.135 |
| 232632_at | AU145719 | *tm4sf8/tspan3* | Tetraspanin 3 | 2.747 |  |
| 235146_at | N51717 | *tmcc3* | Transmembrane and coiled-coil domain family 3 | -3.657 |  |
| 223595_at | AF247167 | *tmem133* | transmembrane protein 113 |  | 2.950 |
| 233480_at | AK026869 | *tmem43* | Transmembrane protein 43 | -3.736 |  |
| 1557064_s_at | AW328331 | *tmem76* | transmembrane protein 76 |  | 3.215 |
| 223949_at | AB038160 | *tmprss3* | transmembrane protease, serine 3 | -5.876 |  |
| 244748_at | AW139525 | *tmprss4* | Transmembrane protease, serine 4 |  | -3.879 |
| 231429_at | BE503430 | *tmsb10* | Thymosin, beta 10 |  | -3.017 |
| 1566709_at | AK024940 | *tnfaip8* | Tumor necrosis factor, alpha-induced protein 8 | -4.991 |  |
| 241371_at | AW451259 | *tnfrsf10a* | Tumor necrosis factor receptor superfamily, member 10a | -2.770 |  |
| 221085_at | NM_005118 | *tnfsf15* | tumor necrosis factor (ligand) superfamily, member 15 |  | -3.145 |
| 220415_at | NM_015978 | *tnni3k* | TNNI3 interacting kinase | 4.926 |  |
| 239404_at | BF840360 | *top1* | Topoisomerase (DNA) I |  | -3.833 |
| 233851_s_at | AJ299441 | *tor3a* | torsin family 3, member A |  | -3.099 |
| 241400_at | AI860360 | *tpm1* | tropomyoxin 1 (alpha) |  | 6.250 |
| 202871_at | NM_004295 | *traf4* | TNF receptor-associated factor 4 | 3.788 |  |
| 1569861_at | BC032830 | *traf5* | TNF receptor-associated factor 5 | -5.710 |  |
| 238395_at | AI254013 | *tram2* | translocation associated membrane protein 2 |  | 2.933 |
| 234456_at | AK025623 | *trappc4* | trafficking protein particle complex 4 |  | -4.041 |
| 239818_x_at | AA576947 | *trib1* | tribbles homolog 1 (Drosophila) |  | -12.070 |
| 215945_s_at | BC005016 | *trim2* | tripartite motif-containing 2 | -3.190 |  |
| 239716_at | AA908970 | *trim33* | Tripartite motif-containing 33 |  | 7.092 |
| 1564950_at | BC008387 | *trim35* | Tripartite motif-containing 35 |  | 5.128 |
| 1554001_at | BC036012 | *trim37* | tripartite motif-containing 37 |  | 2.950 |
| 1554182_at | BC033871 | *trim50* | tripartite motif-containing 50B ; tripartite motif-containing 50C |  | -4.144 |
| 236845_at | AI479391 | *trim62* | tripartite motif-containing 62 |  | 2.747 |
| 216700_at | AL161955 | *trio* | Triple functional domain (PTPRF interacting) | 6.536 |  |
| 209010_s_at | AI797657 | *trio* | triple functional domain (PTPRF interacting) | -5.815 |  |
| 240773_at | AW449903 | *trio* | Triple functional domain (PTPRF interacting) | -2.996 |  |
| 224219_s_at | AF063825 | *trpc4* | transient receptor potential cation channel, subfamily C, member 4 |  | 3.367 |
| 1565886_at | W04694 | *trpm7* | Transient receptor potential cation channel, subfamily M, member 7 | 2.740 |  |
| 1565887_at | AF086174 | *trpm7* | Transient receptor potential cation channel, subfamily M, member 7 |  | 4.329 |
| 234351_x_at | AK000948 | *trps1* | trichorhinophalangeal syndrome I |  | -2.926 |
| 237513_at | AI807285 | *try1* | trypsin X3 | -3.788 |  |
| 1560648_s_at | AI625538 | *tspyl1* | TSPY-like 1 | 2.924 |  |
| 229388_at | AW274240 | *ttc18* | Tetratricopeptide repeat domain 18 |  | -2.510 |
| 240716_at | AI014546 | *ttc23* | tetratricopeptide repeat domain 23 |  | -2.803 |
| 210614_at | U21938 | *ttpa* | tocopherol (alpha) transfer protein (ataxia (Friedreich-like) with vitamin E deficiency) |  | 5.128 |
| 217396_at | AL133075 | *tub* | Tubby homolog (mouse) |  | -5.129 |
| 230535_s_at | BF109387 | *tubb1* | Tubulin, beta 1 |  | -3.403 |
| 232372_at | AL157491 | *tulp4* | Tubby like protein 4 | -7.596 |  |
| 232372_at | AL157491 | *tulp4* | Tubby like protein 4 |  | 2.513 |
| 240118_at | AI401105 | *txndc11* | Thioredoxin domain containing 11 |  | -4.565 |
| 208958_at | AI827677 | *txndc4* | thioredoxin domain containing 4 (endoplasmic reticulum) | 2.667 |  |
| 1566935_at | X72887 | *tyro3p* | TYRO3P protein tyrosine kinase pseudogene |  | 3.906 |
| 238802_at | AA470128 | *tysnd1* | trypsin domain containing 1 | -2.574 |  |
| 214171_s_at | AI810156 | *u2af2* | U2 (RNU2) small nuclear RNA auxiliary factor 2 |  | -3.320 |
| 202151_s_at | NM_016172 | *ubadc1* | ubiquitin associated domain containing 1 | 3.448 |  |
| 1562878_at | AK098632 | *ube2d3* | Threonine synthase, chloroplast | -3.549 |  |
| 242403_at | AI459177 | *ube2d3* | Ubiquitin-conjugating enzyme E2D 3 (UBC4/5 homolog, yeast) |  | 3.135 |
| 243046_at | BF679700 | *ube2d3* | Ubiquitin-conjugating enzyme E2D 3 (UBC4/5 homolog, yeast) |  | 4.219 |
| 230171_at | AW662789 | *ube2d3* | Ubiquitin-conjugating enzyme E2D 3 (UBC4/5 homolog, yeast) |  | 5.988 |
| 240625_at | AW511032 | *ube2e3* | Ubiquitin-conjugating enzyme E2E 3 (UBC4/5 homolog yeast) | -5.478 |  |
| 229483_at | AA760738 | *ube2h* | Ubiquitin-conjugating enzyme E2H (UBC8 homolog, yeast) |  | -5.958 |
| 240240_at | R10087 | *ube2j2* | Ubiquitin-conjugating enzyme E2, J2 (UBC6 homolog, yeast) | -2.565 |  |
| 242673_at | AA931284 | *ube3c* | Ubiquitin portein ligase E3C | -4.912 |  |
| 215533_s_at | AF091093 | *ube4b* | ubiquitination factor E4B (UFD2 homolog, yeast) | 4.717 |  |
| 1569140_at | BC024217 | *ubr2* | ubiquitin protein ligase E3 component n-recognin 2 | -3.085 |  |
| 229248_at | AA020784 | *uchl5* | Ubiquitin carboxyl-terminal hydrolase L5 | -2.852 |  |
| 244814_at | AA232658 | *ugcgl1* | UDP-glucose ceramide glucosyltransferase-like 1 | 5.348 |  |
| 204532_x_at | NM_021027 | *ugt1a* | UDP glucuronosyltransferase 1 family, | 2.710 |  |
| *238248_at* | *AI935789* | *umod* | *Uromodulin (uromucoid, Tamm-Horsfall glycoprotein)* | *-2.140* |  |
| 203271_s_at | NM_005148 | *unc119* | unc-119 homolog (C. elegans) |  | 3.534 |
| 230210_at | AW054783 | *unc84a* | unc-84 homolog A (C. elegans) |  | -3.076 |
| 1555034_at | AF482697 | *ush3a* | Usher syndrome 3A | -3.671 |  |
| 237439_at | R45656 | *usp43* | ubiquitin specific peptidase 43 | -2.917 |  |
| 243175_at | BF696056 | *uts2d* | urotensin 2 domain containing |  | -3.076 |
| 1569808_at | BC036438 | *vdac2* | voltage-dependent anion channel 2 | -2.809 |  |
| 208844_at | BC002456 | *vdac3* | voltage-dependent anion channel 3 | -3.123 |  |
| 229759_s_at | AA988323 | *veph1* | ventricular zone expressed PH domain homolog 1 (zebrafish) |  | 5.848 |
| 204376_at | NM_014703 | *vprbp* | Vpr-binding protein |  | 4.878 |
| 227987_at | AW629014 | *vps13a* | vacuolar protein sorting 13A (yeast) |  | 2.874 |
| 243764_at | AW085312 | *vsig1* | V-set and immunoglobulin domain containing 1 |  | 3.817 |
| 219768_at | NM_024626 | *vtcn1* | V-set domain containing T cell activation inhibitor 1 | 3.067 |  |
| 239916_at | AI381496 | *wdr16* | WD repeat domain 16 |  | -4.249 |
| 223146_at | BE222527 | *wdr33* | WD repeat domain 33 | -2.566 |  |
| 1568840_at | BC035190 | *wdsof1* | WD repeats and SOF1 domain containing |  | 3.333 |
| 201295_s_at | BF111821 | *wsb1* | WD repeat and SOCS box-containing 1 |  | -5.130 |
| 241626_at | BE148503 | *wtap* | Wilms tumor 1 associated protein | -4.149 |  |
| 240168_at | AA679589 | *xpo7* | exportin 7 |  | -3.273 |
| 208459_s_at | NM_015024 | *xpo7* | exportin 7 |  | -2.949 |
| 1570394_at | BC039314 | *xrn1* | 5'-3' exoribonuclease 1 | -3.323 |  |
| 232216_at | AA828049 | *yme1l1* | YME1-like 1 (S. cerevisiae) |  | -2.695 |
| 1568680_s_at | BC019100 | *ythdc2* | YTH domain containing 2 |  | -3.101 |
| 237548_at | AW294215 | *zak* | Sterile alpho motif and leucine zipper containing kinase AZK | 4.065 |  |
| 235491_at | BF056208 | *zbtb10* | Zinc finger and BTB domain containing 10 |  | -4.596 |
| 1554037_a_at | BC036731 | *zbtb24* | zinc finger and BTB domain containing 24 | 2.571 |  |
| 231547_at | AI954694 | *zbtb37* | zinc finger and BTB domain containing 37 |  | -4.148 |
| 220104_at | NM_020119 | *zc3hav1* | zinc finger CCCH-type, antiviral 1 | -2.813 |  |
| 239644_at | AW338214 | *zc3hdc8* | Zinc finger CCCH-type containing 8 |  | -2.902 |
| 225595_at | AL110236 | *zf* | HCF-binding transcription factor Zhangfei |  | -5.734 |
| 234300_s_at | AF226995 | *zfp28* | zinc finger protein 28 homolog (mouse) | 3.676 |  |
| 234937_x_at | AC007228 | *zfp28* | zinc finger protein 28 homolog (mouse) |  | 2.985 |
| 211463_at | AF332509 | *zic4* | Zic family member 4 | -4.957 |  |
| 235366_at | BF970692 | *znf10* | zinc finger protein 10 |  | -2.752 |
| 1552946_at | NM_153608 | *znf114* | zinc finger protein 114 | -7.517 |  |
| 1562743_at | BC042873 | *znf11b* | Zinc finger protein 11B |  | -4.215 |
| 207402_at | NM_003433 | *znf132* | zinc finger protein 132 (clone pHZ-12) |  | 2.710 |
| 210282_at | AL136621 | *znf198* | zinc finger protein 198 |  | 7.576 |
| 240995_at | AW665316 | *znf211* | Zinc finger protein 211 | -3.868 |  |
| 217403_s_at | AC074331 | *znf227* | zinc finger protein 227 | -4.638 |  |
| 242210_at | AA749167 | *znf24* | Zinc finger protein 24 (KOX 17) |  | -2.553 |
| 219604_s_at | NM_017715 | *znf3* | zinc finger protein 3 (A8-51) | -3.219 |  |
| 1569251_a_at | BC040378 | *znf333* | zinc finger protein 333 |  | 5.051 |
| 226261_at | AI831561 | *znrf2* | zinc and ring finger 2 | 3.922 |  |
| 210659_at | U79526 |  | Human orphan G-protein | 2.857 |  |
| 1555711_x_at | AY039026 |  | Isolate Tree92 immunoglobulin heavy chain variable region (IGVH) | 2.564 |  |
| 211667_x_at | L34698 |  | T-cell receptor active alpha-chain V-region (V-J-C) mRNA, partial cds, clone AF110 | -8.670 |  |
| 1565073_at | AF420439 |  | Chimera 2 |  | 2.717 |
