# Supplementary material for: An approach to analyse the specific impact of rapamycin on mRNA-ribosome association
Source: BMC Med Genomics. 2008 Aug 1;1:33. doi: 10.1186/1755-8794-1-33 (PMC2533349; doi:10.1186/1755-8794-1-33)
Supplement: Additional File 3 — Comparison with the array of Gera and co-workers (7). Transcripts detected in both screens are listed with the fold change. Values are indicated in red when they differ between the two studies. [file 1755-8794-1-33-S3.doc]

**Additional Table 3**: Comparison with the array of Gera

| **TRANSCRIPT** | **CURRENT STUDY** | | |  | | **Gera ARRAY** | |
| --- | --- | --- | --- | --- | --- | --- | --- |
|  | light polysomes |  | heavy polysomes | |  | |  |
| Irf2 (interferon regulatory factor 2) | -4.2 |  |  | |  | | **-4.2** |
| Ube3a (ubiquitin ligase E3A) | -4.9 |  |  | |  | | **-4.1** |
| rpl21 (ribosomal protein L21) | -5.1 |  |  | |  | | **-2.2** |
| **rpl5 (ribosomal protein L5)** | **-1.5** |  |  | |  | | **-3.7** |
| nol1 (nucleolar protein 1) |  |  | -3 | |  | | **-3.1** |
| ptk2 (protein tyrosine kinase 2) |  |  | -4.9 | |  | | **-2.9** |
| **eif4G1 (translation initiation factor 4G)** |  |  | **-1.6** | |  | | **-2.6** |
| hdac2 (histone deacetylase 2) |  |  | -2.8 | |  | | **-2.6** |
| **scamp3 (secretory membrane protein 3)** |  |  | **-1.5** | |  | | **-2.5** |
| **sgcd (sarcoglycan delta)** | **-1.5** |  |  | |  | | **-2.5** |
| cacnb2 (calcium voltage channel) | -5.6 |  |  | |  | | **-2.3** |
| U2afirs (U2 RNP subunit 2) |  |  | -3.3 | |  | | **-2.3** |
| rnp1 (ribophorin I) | -5.1 |  |  | |  | | **-2.2** |
| fyb (FYN binding protein) |  |  | -4.2 | |  | | **-2.1** |
| mapk1 (mitogen activated kinase I) | -2.6 |  |  | |  | | **2.9** |
| **Camkk2 (Ca/calmodulin kinase kinase 2)** |  |  | **-1.6** | |  | | **2.8** |
| tfap4 (transcription factor AP-4) |  |  | 2.7 | |  | | **2.4** |
| ets2 (v-ets E26 oncogene homologue 2) |  |  | 3.9 | |  | | **2.4** |
| G6pc (glucose-6-phosphatase, catalytic) | -4 |  |  | |  | | **2.3** |
| Ndufb7 (NADH dehydogenase subcomplex 7) | -4.1 |  |  | |  | | **2.3** |
| folr1 (folate receptor 1) | -6.2 |  |  | |  | | **2.1** |
